# Agentic Finance: An Adaptive Inference Framework for Bounded-Rational Investing Agents

**DOI:** 10.3390/e28030321

**Published:** 2026-03-12

**Authors:** Samuel Montañez Jacquez, John H. Clippinger, Matthew Moroney

**Affiliations:** 1 Engineering Department, Universidad Panamericana, Augusto Rodin 498, Mexico City 03920, Mexico; 2 First Principles First, Jefferson, NH 03583, USA; john@fp1.ai; 3 Interlaken Associates LLC, Sarasota, FL 34123, USA; msm@aya.yale.edu

**Keywords:** Adaptive Inference, active inference, bounded rationality, portfolio management, information-theoretic finance, partially observable Markov decision processes, entropic Sharpe ratio, free energy principle, uncertainty quantification, confidence gating

## Abstract

We propose *Adaptive Inference*, a portfolio management framework extending Active Inference to non-stationary financial environments. The framework integrates inference, control, and execution under endogenous uncertainty, modeling investment decisions as coupled dynamics of belief updating, preference encoding, and action selection rather than optimization over fixed objectives. In this approach, portfolio behavior is governed by the expected free energy (EFE) minimization, showing that classical valuation models emerge as limiting cases when epistemic components vanish. Using train–test evaluation on the ARKK Innovation ETF (2015–2025), we identify a Passivity Paradox: frozen belief transfer outperforms naive adaptive learning. A Professional Agent achieves a Sharpe ratio of 0.39 while its adaptive counterpart degrades to −0.28, reflecting belief contamination when learning from policy-dependent signals. Crucially, the architecture is not designed to generate alpha but to perform endogenous risk management that mitigates overtrading under regime ambiguity and distributional shift. Adaptive Inference Agents maintain long exposure most of the time while tactically reducing positions during high-entropy periods, implementing uncertainty-aware passive investing. All agents reduce realized volatility relative to ARKK Buy-and-Hold (43.0% annualized). Cross-asset validation on the S&P 500 ETF (SPY) shows that inference-guided risk shaping achieves a positive Entropic Sharpe Ratio (ESR), defined as excess return per unit of informational work, thereby quantifying the economic value of information under thermodynamic constraints on inference.

## 1. Introduction

### 1.1. The Excess Volatility Puzzle

**Figure 1:**
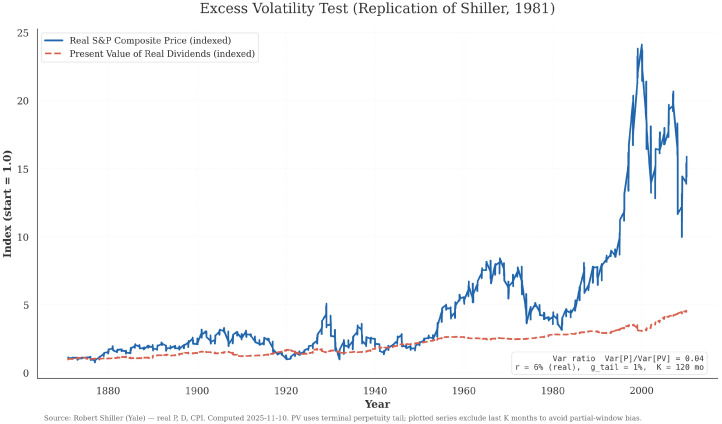
Excess Volatility Test (Replication of Shiller, 1981). Comparison of real S&P Composite Price Index (solid blue) with present value of real dividends (dashed red), indexed to 1.0 at sample start (1871–2020). Present-value series computed as discounted sum of future real dividends at a discount rate of r=6% with terminal perpetuity assuming g=1%. Final K=120 months excluded to avoid partial-horizon bias. The variance ratio $\sigma_{\mathrm{PV}}^{2}/\sigma_{P}^{2}\approx 0.04$ indicates that realized market prices are approximately 25 times more volatile than dividend-implied fundamentals. Source: Yale dataset [2] following Shiller [1] methodology.

Portfolio management confronts a foundational puzzle that has resisted resolution for over four decades. Shiller [1] demonstrated that equity prices fluctuate far more than can be justified by changes in underlying cash-flow fundamentals. Using U.S. equity data, he showed that observed prices exhibit volatility an order of magnitude larger than the present-value benchmark implied by rational expectations. Figure 1 replicates this excess volatility result with updated data, confirming that forces beyond discounted dividends systematically drive prices.

This systematic deviation reveals that traditional finance models which treat uncertainty as exogenous noise to be hedged or diversified fundamentally mischaracterize market dynamics. Asset prices reflect not just cash flow information but the *information-processing behavior* of market participants: how they form beliefs, resolve uncertainty, and adapt to regime shifts [3,4,5,6].

This discrepancy cannot be dismissed as measurement error or transient inefficiency. Instead, it points to a deeper mechanism in which asset prices reflect not only economic fundamentals but the evolving beliefs of market participants, that is, the *Bayesian mechanics* of valuation. Markets are largely shaped by how agents form expectations, resolve uncertainty, and adapt to changing regimes. Any portfolio management theory that treats uncertainty as exogenous noise rather than as an object of inference fundamentally creates structural model inefficiencies.

### 1.2. Belief Dynamics as a Mainstream Economic Problem

Modern economics increasingly recognizes that market behavior is governed by systematic dynamics of belief formation [7,8]. Behavioral finance initially framed departures from rational expectations as biases [9] such as loss aversion, overreaction, or myopia; however, this descriptive catalog leaves open the question of origin. More recent work in cognitive and information economics reframes these phenomena as optimal responses to constraints.

Rational inattention models [10] formalize limits on information processing, while sparse attention [11] derives apparent irrationality from tractable optimization under cognitive constraints. Diagnostic expectations [3,4,5] go further by specifying explicit *laws of motion for beliefs*, generating predictable cycles of overreaction and reversal. In parallel, the Adaptive Markets Hypothesis [12,13] recasts market efficiency as an evolutionary and non-stationary process in which strategies wax and wane with changing environments.

Across these approaches, a common theme emerges: beliefs are not static inputs but endogenous state variables governed by lawful dynamics; what remains missing is a unifying framework that simultaneously (i) treats belief updating and action selection as a single problem, (ii) accounts for information costs endogenously, and (iii) remains robust under policy adaptation.

### 1.3. The Lucas Critique in Portfolio Learning

The Lucas Critique [14] highlights a fundamental limitation of adaptive decision-making: relationships estimated under one policy regime generally fail when the policy itself changes. While originally formulated in macroeconomics for public policy evaluation, the critique applies with equal relevance to portfolio learning.

In portfolio management settings, realized profit and loss (P&L) is not a structural feature of the market; rather, it is mechanically generated by the agent’s own past actions interacting with exogenous returns. Therefore, learning directly from P&L conflates market information with policy feedback. When an agent adapts its strategy, the data-generating process shifts endogenously, rendering previous estimates invalid and inducing unstable belief–policy feedback loops.

This issue is particularly acute for reinforcement learning and adaptive trading systems, which update value functions or beliefs from realized rewards. Without explicitly separating exogenous market signals from endogenous outcomes, adaptive agents risk learning from their own behavior rather than from the market itself—precisely the failure mode highlighted by Lucas.

### 1.4. Active Inference as a Control-Theoretic Framework

Active Inference provides a principled response to these challenges. Originating in theoretical neuroscience [15,16], it formalizes decision-making as the minimization of expected free energy (EFE)—a single objective that unifies belief, behavior, and action. Actions are selected not only to achieve preferred outcomes (pragmatic value) but also to reduce uncertainty about hidden states (epistemic value) [17,18].

Crucially, Active Inference derives exploration endogenously without the ad hoc bonuses found in methods such as reinforcement learning, and embeds information-processing costs via variational and thermodynamic principles; recent work makes these costs explicit, grounding inference in physical limits on computation and energy dissipation [19,20]. In the limit where information costs vanish, classical expected-utility maximization is recovered, and models function similarly to conventional finance models (e.g., Black–Scholes–Merton).

Importantly, Active Inference is not a representationalist theory of belief storage. Generative models function as control systems governing action–perception loops, consistent with the enactive interpretation emphasized by Ramstead et al. [21]. This perspective generalizes Active Inference with general systems theory rather than neurofinance per se, positioning it as a physics-compatible framework for economic agency.

### 1.5. Adaptive Inference and Surgical Learning

We extend Active Inference to financial markets through *Adaptive Inference* (we use Active Inference to refer to the general theoretical framework originating in computational neuroscience [22], and Adaptive Inference to refer to its financial application as developed in this paper). Adaptive Inference inherits the inferential and control machinery of Active Inference—belief updating via variational free energy, action selection via expected free energy (EFE)—but incorporates domain-specific extensions: surgical learning to address the Lucas Critique, confidence gating for execution under uncertainty, and strict separation of exogenous and endogenous observation channels. The two terms are not interchangeable. The Adaptive Inference framework preserves the core inference–control loop of Active Inference while disciplining it with the causal structure specific to non-stationary economic environments. The key innovation is *surgical learning*, a form of modality-selective Bayesian updating that enforces the Lucas Critique from macroeconomics by construction.

Market returns and auxiliary signals (momentum, volatility, volume) are treated as exogenous observations and remain learnable. By contrast, portfolio P&L is endogenous to the agent’s policy, and is explicitly excluded from learning updates. This separation prevents belief contamination from self-generated outcomes while preserving adaptive capacity where it is structurally valid.

This design yields a sharp theoretical prediction, namely, the *Passivity Paradox*. Agents that adapt naively to endogenous feedback should underperform frozen learning and pretrained policies out of sample; however, when learning is restricted to exogenous modalities, adaptation becomes beneficial rather than destabilizing. Our empirical results confirm both sides of this prediction.

### 1.6. Contributions and Roadmap

This paper makes five main contributions. First, we show that mean–variance optimization, the Black–Scholes–Merton Real Options model, and Stochastic DCF valuation emerge as limiting cases of Adaptive Inference when epistemic value is suppressed, establishing a formal bridge between classical finance and expected free energy (EFE) minimization.

Second, we provide a principled resolution of the Lucas Critique [14] in portfolio learning via surgical learning, separating endogenous belief updating from exogenous information.

Third, we introduce the Entropic Sharpe Ratio (ESR), a performance measure that quantifies excess return per unit of informational work.

Fourth, we demonstrate empirically that adaptive learning is beneficial only within well-defined boundary conditions, validating the Passivity Paradox.

Fifth, we benchmark Adaptive Inference against a provably adaptive online learning algorithm: the multiplicative-weights (Hedge) expert mixture [23,24]. This comparison shows that inference-guided control achieves competitive risk-adjusted returns while uniquely providing belief–state transparency and ante hoc decision auditability, both properties that are absent from model-free adaptive methods.

The remainder of the paper proceeds as follows: Section 2 develops the theoretical foundations; Section 3 details the POMDP architecture and learning protocol; Section 4 presents empirical results; Section 5 discusses implications; and Section 6 concludes the paper.

## 2. Theoretical Framework

### 2.1. Economic Agency Under Partial Observability

We model portfolio management as sequential decision-making under partial observability, where an economic agent interacts with a stochastic environment characterized by latent market states, observable signals, and controllable portfolio actions. Hidden states encode persistent but unobserved market regimes, while observations consist of realized returns and auxiliary market signals. This formulation aligns with standard treatments of regime uncertainty in financial economics [25].

The agent is assumed to be a *price taker*, such that individual portfolio decisions do not influence market prices. This standard assumption in portfolio theory [26,27] allows for a sharp separation between exogenous market dynamics and endogenous portfolio outcomes. The agent’s objective is not static utility maximization but the regulation of exposure over time under uncertainty, aligning portfolio management with stochastic control rather than one-shot optimization [26,28].

### 2.2. Generative Processes and Generative Models

Following Active Inference, we distinguish between the *generative process*—the true, unknown market dynamics—and the *generative model*, which specifies how beliefs, actions, and observations are coupled for the purpose of policy selection [22,29]. The generative model is a normative control architecture, not a representational description of the market.

Formally, the generative model factorizes as

(1) $$P\left(o_{1:T},s_{1:T},\pi \right)=P\left(\pi \right)\;P\left(s_{1}\right)\;\prod_{t=1}^{T}P\left(o_{t}|s_{t}\right)\;\prod_{t=2}^{T}P\left(s_{t}|s_{t-1},\pi \right),$$

where $P(o_{t}|s_{t})$ is the likelihood (the *A*-matrix), $P(s_{t}|s_{t-1},\pi )$ is the policy-conditioned transition model (the *B*-matrices), $P(s_{1})$ is the initial-state prior (the *D*-vector), and P(π) is the prior over policies, parameterized by an *E*-vector with one entry per policy, encoding habitual or default policy preferences [30].

Consistent with enactive interpretations of Active Inference, the generative model functions as a control system governing action–perception loops rather than as an internal representation of external structure [21]. Internal states parameterize an approximate posterior (recognition density) that regulates control and adaptation.

### 2.3. Expected Free Energy and Policy Selection

Action selection is governed by the minimization of *expected free energy* (EFE) over policies π

(2) $$G\left(\pi \right)=E_{Q(o_{t:t+H},\;s_{t:t+H}|\pi )}\left[\;\ln Q\left(s_{t:t+H}|\pi \right)-\ln P\left(o_{t:t+H},\;s_{t:t+H}|\pi \right)\right],$$

where $s_{t}$ denotes latent (unobserved) market states, $o_{t}$ denotes observable market outcomes, and Q(·) is the agent’s variational (approximate posterior) belief over future state and observation trajectories under policy π. The generative model $P(o_{t:t+H},s_{t:t+H}|\pi )$ specifies how observations are expected to arise from hidden states given policy-dependent dynamics. The dependence on π reflects that future state transitions are controlled by the agent’s actions. Expectations are taken with respect to predicted trajectories rather than realized ones over a finite planning horizon *H* from time *t* to t+H.

EFE allows for a natural decomposition into epistemic and pragmatic components [17,31,32]:

(3) $$\begin{array}{cc} G(\pi )= & \underset{\mathrm{Epistemic}\;\mathrm{value}\;(\mathrm{expected}\;\mathrm{information}\;\mathrm{gain})}{\underset{︸}{-E_{Q(o_{t:t+H}|\pi )}\;\left[D_{\mathrm{KL}}\;\left(Q\left(s_{t:t+H}|o_{t:t+H},\pi \right)\;∥\;Q\left(s_{t:t+H}|\pi \right)\right)\right]}}\\ & +\;\underset{\mathrm{Pragmatic}\;\mathrm{value}\;(\mathrm{expected}\;\mathrm{value})}{\underset{︸}{E_{Q(o_{t:t+H}|\pi )}\;\left[-\ln P(o_{t:t+H}|C)\right]}}. \end{array}$$

Policies are selected according to a precision-weighted posterior

(4) Q(π)=σ(lnP(π)−γG(π)),

where σ(·) denotes a softmax operator and γ>0 is the policy precision (inverse temperature) controlling the determinism of policy selection. The vector *C* encodes prior preferences over observable outcomes, such that $P(o_{t:t+H}|C)$ assigns higher probability to preferred outcome trajectories. Higher γ yields more exploitative behavior (value-seeking) with lower-entropy policy posteriors, while lower γ induces stochastic exploration (information-seeking) [30].

### 2.4. Thermodynamic Interpretation and Information Costs

Expected free energy minimization has a thermodynamic interpretation. Belief updating and policy evaluation correspond to physical information processing, which incurs energetic costs [33,34]. Therefore, reductions in posterior entropy require work, implying that inference precision is fundamentally constrained.

This perspective formalizes bounded rationality in the sense of Simon [35,36] as a physical necessity rather than a behavioral anomaly. Instead of assuming global utility maximization, agents are constrained by limited information, finite computational capacity, and the costs of inference itself. Recent work has made these constraints explicit within Active Inference, demonstrating how belief updating and precision modulation entail measurable entropy production [19,20,37]. These explicit entropy considerations motivate evaluating portfolio performance relative to information cost, a principle operationalized later through the Entropic Sharpe Ratio to enable direct comparison on this basis.

### 2.5. Endogeneity, Controllability, and the Lucas Critique

#### Controllability Decomposition

Classical asset pricing frameworks decompose returns by *risk source*. For example, the Capital Asset Pricing Model (CAPM) [27] expresses excess returns as

$$R_{i,t}-R_{f}=\beta_{i}\left(R_{m,t}-R_{f}\right)+\varepsilon_{i,t},$$

where $R_{i,t}$ denotes the return on asset *i* at time *t*, $R_{f}$ is the risk-free rate, $R_{m,t}$ is the market portfolio return, $\beta_{i}$ measures the asset’s exposure to systematic market risk, and $\varepsilon_{i,t}$ captures idiosyncratic return variation orthogonal to the market. The CAPM decomposition separates systematic risk from asset-specific noise and prescribes maximization of expected return per unit of systematic risk exposure.

Adaptive Inference introduces a fundamentally different decomposition via *controllability*. At the portfolio level, realized P&L can be written as

(5) $$P\&L_{t}=\underset{\mathrm{controllable}\;\mathrm{position}}{\underset{︸}{w_{t-1}}}\times \underset{\mathrm{uncontrollable}\;\mathrm{environment}}{\underset{︸}{R_{m,t}}},$$

where $w_{t-1}$ denotes the portfolio weight selected by the agent and $R_{m,t}$ denotes the realized market return.

Market returns are uncontrollable environmental signals; the agent cannot influence them, yet they carry information about latent market regimes. By contrast, the portfolio P&L is controllable through position choice, that is, the agent determines its exposure to the market regime that predicts. This lens shifts the optimization problem from from “accept systematic risk and diversify idiosyncratic risk” to

*infer latent market regimes from uncontrollable observations, and select controllable positions that align exposure with inferred regimes.*

Under this refined problem statement, portfolio management becomes *inference-guided control*. Beliefs should be updated using uncontrollable signals that preserve causal invariance, while actions should operate solely on controllable variables. Violating this separation—by updating beliefs using policy-dependent outcomes such as portfolio P&L—introduces *endogenous* feedback, causing the agent to learn from the consequences of its own actions rather than exogenous market signals, thereby corrupting inference.

In plain terms, surgical learning means that the agent updates its beliefs only from market signals it cannot influence—such as returns, volatility, and momentum—while excluding policy-dependent outcomes such as portfolio P&L from belief updating. This prevents a circular feedback loop: if the agent were to update its market beliefs from its own profits and losses, any change in trading strategy would alter the data-generating process itself, invalidating previously learned relationships. By restricting learning to signals that remain invariant under policy changes, surgical learning preserves causal stability in belief formation while still allowing P&L to inform policy evaluation through preferences.

This controllability-based decomposition provides the economic basis for our learning restrictions. It clarifies why updating naïve adaptive belief violates Lucas’s critique [14] in portfolio learning: when policies change, the distribution of controllable outcomes necessarily changes with them. Learning from such policy-dependent signals conflates the environmental structure with the consequences of the agent’s own actions. By restricting belief updating to uncontrollable policy-invariant information, Adaptive Inference preserves the causal structure of the environment and ensures that inference remains valid under policy shifts.

**Figure 2:**
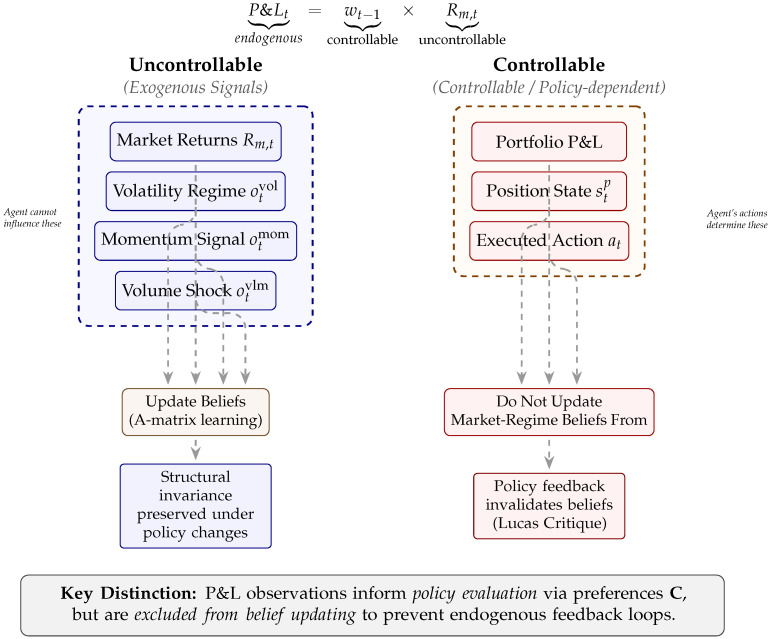
Controllability Decomposition for Surgical Learning. Information sources are classified by whether the agent can influence them. *Uncontrollable* (exogenous) signals—market returns, volatility, momentum, and volume—provide structural information about latent market regimes and are used for belief updating (A-matrix learning). *Controllable* (endogenous) outcomes—P&L, position state, and execution—depend on the agent’s own policy and are excluded from belief updating to prevent circular feedback. The restriction applies to belief updating (A-matrix learning), not to policy evaluation or preference encoding (C-matrix learning); P&L observations still inform action selection through the preference structure $C^{\mathrm{pnl}}$.

Figure 2 illustrates the controllability-based decomposition underlying surgical learning. This decomposition provides a principled response to the Lucas Critique: learning is confined to signals that remain structurally invariant under policy changes.

### 2.6. Controllability as a Structural Distinction

A key insight underlying Adaptive Inference is that endogeneity in portfolio learning arises from a failure to distinguish *controllable* from *uncontrollable* quantities. Market returns and derived price-based signals are environmental variables that the agent cannot influence, and as such constitute valid sources of information about latent market regimes. By contrast, portfolio positions are internal control variables selected by the agent. Portfolio outcomes combine both elements, and as a consequence are intrinsically policy-dependent. This distinction aligns portfolio decision-making with control-theoretic formulations of economic agency under partial observability [26,29].

Unlike the traditional decomposition of expected returns into systematic and idiosyncratic risk [27], Adaptive Inference classifies information by whether it remains invariant under changes in policy. Rather than organizing uncertainty by covariance structure, as in mean–variance portfolio optimization [38], Adaptive Inference distinguishes signals according to their controllability. Only uncontrollable signals preserve their informational meaning when the agent’s policy changes. This principle mirrors the structural invariance requirements emphasized in econometric policy evaluation [14] as well as in modern treatments of causal learning under intervention [39,40].

A practical consequence of controllability-based decomposition is that portfolio P&L cannot be treated as a primitive market signal. Instead, it is a mechanically constructed quantity that is jointly determined by exogenous returns and the agent’s own position choices. Using the notation from Equation (5), P&L can be written as

(6) $$P\&L_{t}=w_{t-1}\cdot R_{m,t},$$

where $w_{t-1}$ denotes the position selected by the agent at time t−1 and $R_{m,t}$ denotes the realized market return. Because $w_{t-1}$ is policy-dependent, the distribution of P&L necessarily changes when the agent’s investing policy changes, even if the underlying return process remains fixed. Therefore, treating P&L as a learning signal induces spurious feedback into belief updates. Empirically, this mechanism aligns with robust out-of-sample dominance of simple allocation rules such as the 1/N portfolio over optimized strategies, a pattern widely interpreted as a manifestation of the Lucas Critique in portfolio choice [14,41].

### 2.7. Classical Finance as Limiting Cases

Adaptive Inference subsumes several canonical models in financial economics as *edge cases* of expected free energy minimization, obtained by suppressing epistemic objectives and uncertainty-sensitive control.

**Proposition** **1** (Limiting Cases of expected free energy (EFE) Minimization)**.** *Under Expected free energy (EFE) minimization:*

*1.*  *Portfolio mean–variance optimization emerges when the planning horizon collapses to a single period and epistemic value is suppressed, yielding quadratic utility over returns [38].*
*2.*  *Black–Scholes–Merton (BSM) Real Options valuation arises when regime uncertainty is zeroed out, volatility is known with certainty, and epistemic value vanishes. In this limit, beliefs become risk-neutral and option values reduce to closed-form pricing under fixed diffusion dynamics [42,43].*
*3.*  *Stochastic Discounted Cash Flow (DCF) valuation corresponds to fixed beliefs over cash flow growth and discount rates, eliminating belief updating and epistemic control. Valuation reduces to the discounted expectation of future cash flows under passive dynamics [44].*
*4.*  *Rational expectations equilibria emerge in the limit of zero information cost and infinite precision, where beliefs instantaneously coincide with the data-generating process [45].*

Crucially, these limits establish mathematical correspondences rather than claims of empirical or normative optimality. As demonstrated empirically in Appendix G.1 and Appendix G.2, suppressing epistemic objectives yields valuation and control policies that are mathematically coherent but fragile under regime instability. In particular, the Black–Scholes–Merton Real Options and Stochastic DCF limits exhibit instability when regime uncertainty and growth persistence are incorrectly assumed to be fixed, known, or time-invariant, while Adaptive Inference retains robustness by explicitly incorporating and balancing pragmatic and epistemic value.

Therefore, Adaptive Inference does not compete with classical finance; it *completes it* by embedding standard valuation models within an information-theoretic control framework that makes uncertainty, learning, and adaptation explicit. From an information-theoretic perspective, classical finance corresponds to zero value limits of the expected free energy (EFE) functional, where uncertainty is treated as exogenous rather than actionable.

### 2.8. Relationship to Online Learning

The limiting-case hierarchy above concerns models that can be recovered from within the expected free energy (EFE) functional by suppressing specific epistemic terms. A complementary class of adaptive methods, most notably the multiplicative-weights (Hedge) algorithm [23,24], does not arise as a limit of expected free energy (EFE) minimization. Hedge is a model-free adversarial online learning procedure with worst-case regret guarantees. It adapts by reallocating weight across a fixed panel of strategies, but does not maintain or update a generative model of latent market states.

Accordingly, Hedge is adaptive in a purely performance-based sense; it learns which strategies perform well without representing why. It lacks belief-state decomposition, epistemic value, and information-seeking behavior. As shown empirically in Section 4.1.1, Hedge achieves competitive risk-adjusted returns under identical execution conventions, ensuring that the performance of Adaptive Inference is not attributable to mechanical advantages. The distinction is structural rather than parametric; inference-guided control embeds learning within a model of latent regimes, whereas adversarial online learning aggregates performance without such representation.

### 2.9. Summary

This section established Adaptive Inference as a control-theoretic framework for multi-period portfolio management under uncertainty. By unifying belief updating and action selection through expected free energy (EFE) and then enforcing a structural separation between exogenous and endogenous information, the framework remains robust to policy adaptation and non-stationarity. The next section operationalizes these principles within a Partially Observable Markov Decision Process (POMDP) model architecture.

## 3. Methodology

This section specifies the Adaptive (Active) Inference portfolio agent as a Partially Observable Markov Decision Process (POMDP) following [30], including: (i) the state, observation, and action spaces; (ii) the generative model primitives {A,B,C,D,E}; (iii) variational state inference and expected free energy (EFE) planning; (iv) surgical learning to enforce robust adaptation; (v) execution and risk controls that translate discrete actions into portfolio weights; and (vi) a train–test experimental protocol to estimate out-of-sample model error and performance.

### 3.1. POMDP Formulation and Problem Setup

**Figure 3:**
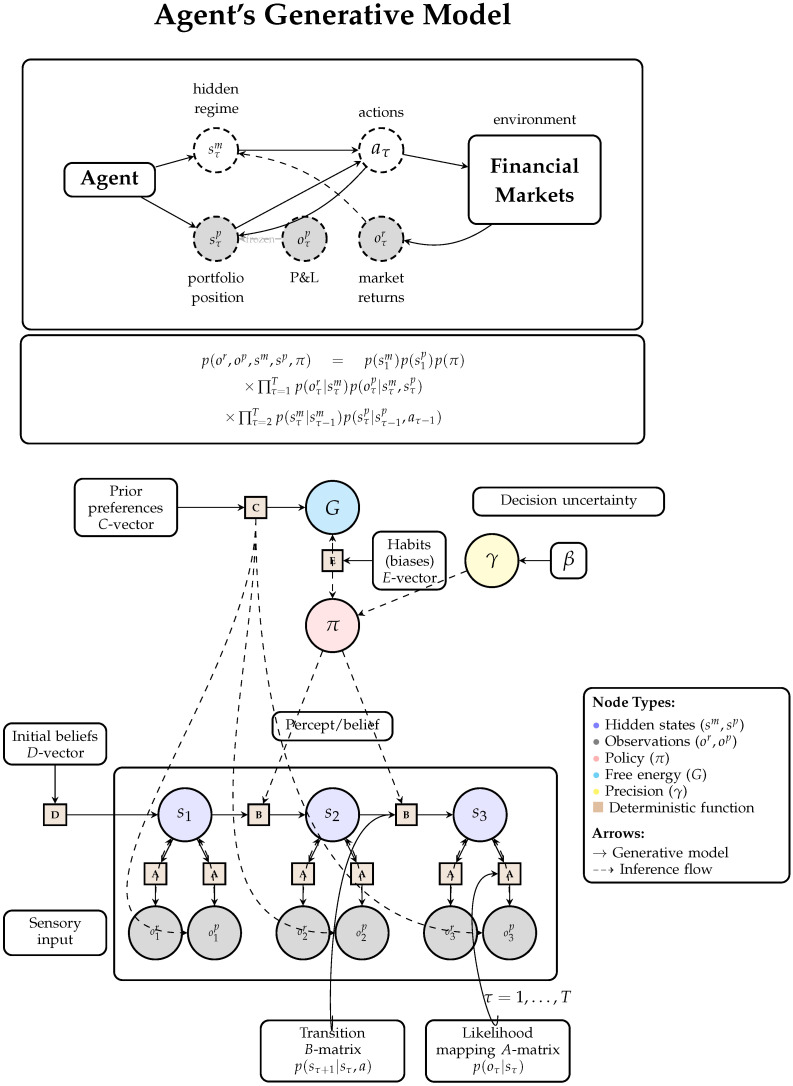
Mathematical specifications for a two-factor Active Inference POMDP (reduced to two-modalities for visual clarity). The generative model separates hidden market regimes ($s^{m}$) from observable positions ($s^{p}$), with dual observation modalities for returns ($o^{r}$) and P&L ($o^{p}$). Market regimes evolve independently via $B^{\mathrm{market}}$, while positions are controlled through actions via $B^{\mathrm{position}}$. In our implementation, the likelihood and transition models (*A* and *B*) are represented as tensors in order to accommodate multiple observation modalities and action-conditioned dynamics, respectively, with each tensor comprising a set of matrices indexed by modality or action. The expected free energy (EFE) G(π) decomposes into epistemic value (information gain about regimes) and pragmatic value (expected P&L utility). This formulation enables simultaneous inference about uncontrollable environmental dynamics and learning about action effectiveness.

We formalize portfolio management as a Partially Observable Markov Decision Process (POMDP; Figure 3), following the Active Inference formulation [30] of sequential decision-making [46,47]. At each decision time *t*, the agent:

1. Receives market observations;
2. Updates beliefs over latent market regimes;
3. Evaluates candidate policy sequences over a finite horizon; and
4. Executes (or abstains from) an action under uncertainty-modulated precision control.

Figure 3 represents the full generative model of the agent, including the factorization of hidden states into latent market regimes and observable portfolio positions, dual observation modalities for returns and P&L, and the policy-dependent transition dynamics that govern the control of position size. The key modeling decision constraint is *controllability*: market outcomes are exogenous to the agent, while portfolio positions are internal (endogenous) control variables. This distinction motivates the structural separation between exogenous learning channels and endogenous portfolio feedback, formalized in the surgical learning protocol (Section 3.9).

**Figure 4:**
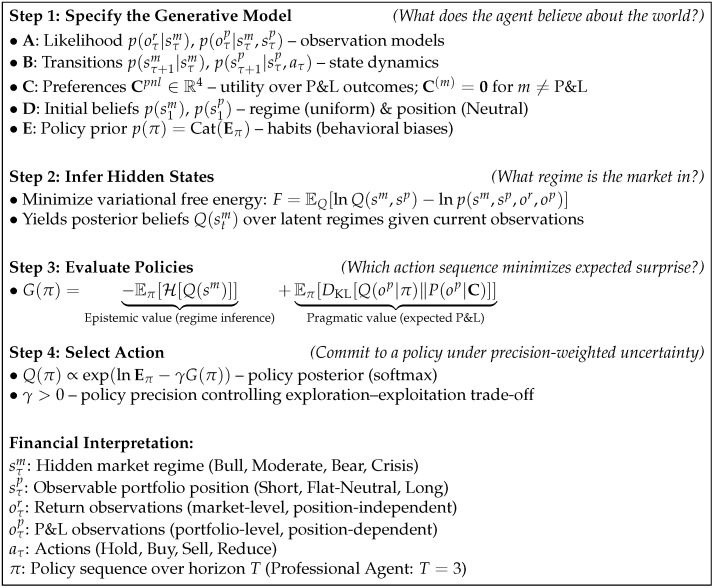
Step-by-step perception–action cycle for the Adaptive Inference agent. The agent first specifies its generative model (Step 1), then infers hidden market regimes from observations by minimizing variational free energy (Step 2), evaluates candidate policies by the expected free energy (EFE) decomposed into epistemic and pragmatic components (Step 3), and selects an action under precision-weighted softmax (Step 4). Steps 2–4 repeat at each decision time *t*.

A step-by-step formalization of this perception–action cycle, including the generative model specification and policy evaluation equations, is provided in Figure 4.

### 3.2. State Space

The latent state factorizes into (i) an uncontrollable *market* regime state and (ii) a controllable *position sizing* state:

(7) $$S\;=\;S^{m}\times S^{p},$$

where $S^{m}=\left\{\mathrm{Bull},\mathrm{Moderate},\mathrm{Bear},\mathrm{Crisis}\right\}$ and $S^{p}=\left\{\mathrm{Short},\mathrm{Flat},\mathrm{Long}\right\}$. The joint state space has cardinality (number of discrete states) |S|=12.

We maintain a mean-field posterior factorization

(8) $$Q\left(s_{t}\right)\;=\;Q\left(s_{t}^{m}\right)\;⊗\;Q\left(s_{t}^{p}\right),$$

which yields tractable inference while preserving the economic separation between exogenous market dynamics and controllable portfolio configuration [29,48].

### 3.3. Observability of Position

In the implementation, the current portfolio position is known to the agent (it is the executed portfolio weight), but we retain $s^{p}$ as a state factor because (i) it governs controllable transitions via the action-conditioned transition model for the position state factor $B^{p}\left(\cdot |a\right)$, and (ii) it interacts with the market regime in the P&L likelihood $A^{pnl}$. When position is observed, $Q(s_{t}^{p})$ is effectively degenerate (one-hot) at the realized position state.

#### Observation Space

The observation vector comprises five modalities

(9) $$o_{t}\;=\;\left(o_{t}^{ret},\;o_{t}^{pnl},\;o_{t}^{mom},\;o_{t}^{vol},\;o_{t}^{vlm}\right),$$

with discrete outcome spaces $|O^{ret}|\;=4$, $|O^{pnl}|\;=4$, $|O^{mom}|\;=3$, $|O^{vol}|\;=3$, $|O^{vlm}|\;=2$.

Primary modalities.
  - **Returns** $o_{t}^{ret}\in \left\{0,1,2,3\right\}$ (*unrealized market returns*): Discretized market returns via an adaptive rolling-window quantile scheme.
  - **P&L** $o_{t}^{pnl}\in \left\{0,1,2,3\right\}$ (*realized portfolio gains/losses*): Discretized portfolio outcomes capturing regime × position interactions.

Auxiliary modalities.
  - **Momentum** $o_{t}^{mom}\in \left\{0,1,2\right\}$: Tercile-coded momentum regime.
  - **Volatility** $o_{t}^{vol}\in \left\{0,1,2\right\}$: Tercile-coded realized volatility regime.
  - **Volume** $o_{t}^{vlm}\in \left\{0,1\right\}$: Binary abnormal-volume indicator.

### 3.4. Action Space and Policies

Actions are discrete portfolio adjustments:

(10) A={Hold,Buy,Sell,Reduce}.

A policy π is a finite action sequence over planning horizon *T*:

(11) $$\pi \;=\;(a_{t},\ldots ,a_{t+T-1}),\;\pi \in \Pi .$$

Different agent variants use different horizons *T* and policy set sizes (Section 3.10).

### 3.5. Generative Model

Following Active Inference [17,30,31,48], the agent specifies a generative model over states, observations, and policies:

(12) $$p\left(\tilde{o},\tilde{s},\pi \right)\;=\;p\left(s_{t}^{m}\right)\;p\left(s_{t}^{p}\right)\;p\left(\pi \right)\prod_{\tau =t}^{t+T-1}p\left(o_{\tau }|s_{\tau }\right)\prod_{\tau =t+1}^{t+T-1}p\left(s_{\tau }|s_{\tau -1},a_{\tau -1}\right).$$

The model is parameterized by the following, in which we distinguish between the real time *t* at which decisions are made and the dummy index τ, which enumerates future steps along counterfactual policy rollouts used for planning:

- A: Likelihood mappings $p(o_{t}^{\left(m\right)}|s_{t}^{m},s_{t}^{p})$ for each modality *m*;
- $B^{m}$: Market regime transitions $p(s_{t+1}^{m}|s_{t}^{m})$ (uncontrollable);
- $B^{p}\left(\cdot |a_{t}\right)$: Position transitions $p(s_{t+1}^{p}|s_{t}^{p},a_{t})$ (controllable);
- C: Preferences (utility) over outcomes, applied to the *endogenous* P&L modality;
- D: Initial state priors over $\left(s_{t}^{m},s_{t}^{p}\right)$ at episode start;
- E: A prior over policies p(π) encoding habits/biases.

A complete matrix-level specification of A,B,C,D,E for all agent types is provided in Appendix A and Appendix C.

### 3.6. Markov Blanket Market Definition

**Figure 5:**
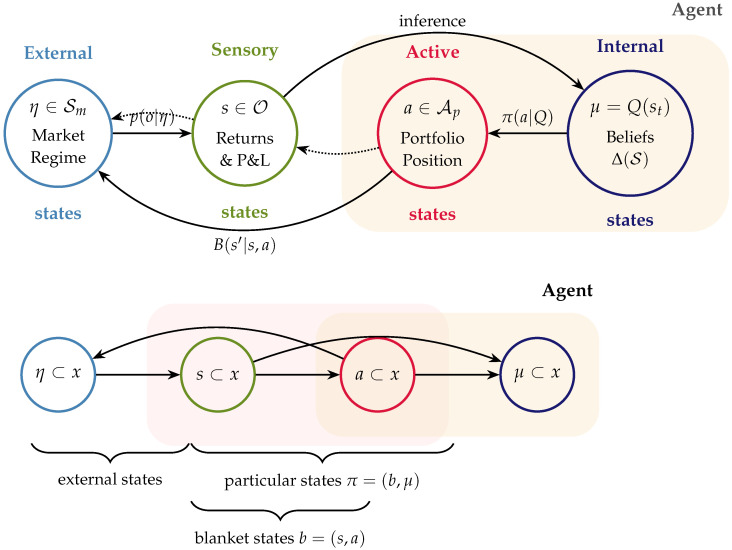
Markov blanket representation of portfolio management under Adaptive Inference. *Upper panel*: The market evolves through latent regimes η (external states), which generate observable signals *s* (returns, volatility, P&L). The agent forms internal beliefs μ about these regimes and selects portfolio positions *a* accordingly. Actions influence future market states, while observations update beliefs. This loop, consisting of regime → observations → beliefs → actions → regime, captures the feedback structure that motivates surgical learning. *Lower panel*: The system is partitioned into external states (η), internal states (μ), and blanket states b=(s,a). The blanket mediates all interactions; external states influence the agent only through sensory variables *s*, and the agent influences the market only through actions *a*. This conditional independence structure defines the Markov blanket and clarifies which signals are exogenous and which are policy-dependent.

We recast the portfolio problem using the formalism of Markov blankets (Figure 5; [21,32,49]). External states correspond to latent market regimes, sensory states to market observations, active states to portfolio positions, and internal states to posterior beliefs. This partition makes explicit the conditional independencies that define the agent–market boundary and clarifies which variables are controllable versus which are merely observable.

### 3.7. Inference and Planning by Free Energy Minimization

#### 3.7.1. State Inference

Given observations up to time *t*, the agent updates beliefs over latent states by minimizing the variational free energy:

(13) $$F_{t}\;=\;E_{Q(s_{t})}[\;\ln Q\left(s_{t}\right)-\ln p\left(s_{t},o_{t}\right)]$$

which upper-bounds the negative log model evidence and is minimized when the approximate posterior $Q(s_{t})$ matches the true Bayesian posterior over states. In practice, this optimization is implemented via iterative message passing under a mean-field factorization, yielding a computationally tractable approximation to Bayesian filtering [29,50]. The complete coordinate-ascent update procedure is given in Algorithm A2.

#### 3.7.2. Policy Evaluation by Expected Free Energy

Candidate policies are evaluated by the expected free energy (EFE) between policy options, which provides a single objective balancing epistemic value (uncertainty resolution) and pragmatic value (goal attainment):

(14) $$G\left(\pi \right)\;=\;\underset{\mathrm{epistemic}\;\mathrm{value}}{\underset{︸}{-E_{Q(s|\pi )}\left[H[p(o|s)]\right]}}\;+\;\underset{\mathrm{pragmatic}\;\mathrm{value}}{\underset{︸}{D_{\mathrm{KL}}\left[Q(o|\pi )\;∥\;p(o|C)\right]}}$$

following the standard Active Inference formulation [17,31,48]. Policy posteriors are obtained via softmax over the expected free energy (EFE):

(15) $$Q\left(\pi \right)\;\propto \;\exp\left(\ln E_{\pi }-\gamma \;G\left(\pi \right)\right)$$

where γ>0 denotes the policy precision parameter controlling the exploration–exploitation tradeoff. The specification-level implementation of this evaluation procedure is provided in Algorithm A3.

### 3.8. Epistemic–Pragmatic Tradeoff

The relative contribution of epistemic and pragmatic value is modulated by the weight parameter η∈[0,1]:

(16) $$G\left(\pi \right)\;=\;\left(1-\eta \right)\;\underset{\mathrm{pragmatic}\;\mathrm{value}}{\underset{︸}{E_{Q}\left[\;\ln Q\left(o|\pi \right)-\ln p\left(o|C\right)\right]}}\;+\;\eta \;\underset{\mathrm{epistemic}\;\mathrm{value}}{\underset{︸}{\left(-E_{Q}\left[H\left[p\left(o|s\right)\right]\right]\right)}},$$

where higher η favors information-seeking behavior. Importantly, η controls the *structural weighting* between epistemic and pragmatic objectives within expected free energy (EFE), whereas the policy precision parameter γ governs the stochasticity of policy selection given G(π) and does not alter the objective itself. The Professional Agent uses η=1.0 by default given pymdp’s implementation, reflecting active regime exploration balanced against return maximization. When η=0, the agent reduces to a purely exploitative portfolio optimizer (mean–variance, BSM Real Options, and Stochastic DCF limits). Here, the epistemic weight η shapes the composition of the expected free energy (EFE) objective, while the policy precision γ controls the sharpness of policy selection over G(π) without affecting its decomposition.

**Figure 6:**
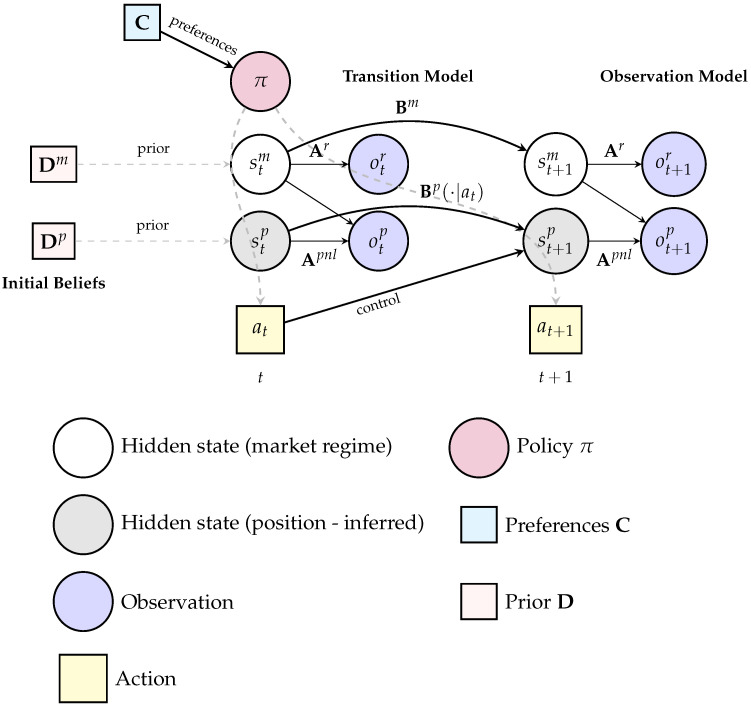
Partially Observable Markov Decision Process (POMDP) representation of the Adaptive Inference portfolio agent. The model distinguishes two hidden state factors. The *market regime* $s_{t}^{m}$ represents latent environmental dynamics (e.g., bull, bear, high-volatility states), and is not directly controllable. The *portfolio position* $s_{t}^{p}$ represents the agent’s internal allocation state, and is action-dependent. At each time *t*, the agent observes returns $o_{t}^{r}$ and realized P&L $o_{t}^{p}$. Return observations depend only on the market regime through the likelihood mapping $A^{r}$. P&L observations depend jointly on the market regime and the portfolio position via $A^{pnl}$. State transitions are factorized into uncontrollable and controllable components. Market regimes evolve autonomously according to $B^{m}$, while portfolio positions evolve according to an action-conditioned transition model $B^{p}\left(\cdot |a_{t}\right)$. Therefore, actions influence future position states but do not directly alter market dynamics. Policies π select actions to minimize the expected free energy (EFE), trading off preference satisfaction (encoded in C) against information gain about hidden states. Initial beliefs over regimes and positions are specified by priors $D^{m}$ and $D^{p}$ and updated online. The key structural feature is the separation between uncontrollable market evolution and controllable portfolio configuration, which enables inference-guided risk management without assuming that actions affect regime dynamics.

Figure 6 provides a temporal graphical representation of this policy evaluation mechanism, illustrating how beliefs over hidden market regimes and portfolio positions propagate from time *t* to t+1, how observations enter via the likelihood mappings, and how policies influence action-dependent position transitions while leaving market dynamics unaffected.

### 3.9. Online Learning and Surgical Learning

Likelihood parameters are updated using Dirichlet learning on the A matrices [30,51,52]. For each modality *m*, concentration parameters $\alpha^{\left(m\right)}$ are updated as follows:

(17) $$\alpha_{t+1}^{\left(m\right)}\;=\;\omega \;\alpha_{t}^{\left(m\right)}\;+\;\eta^{\left(m\right)}\;e_{t}^{\left(m\right)}$$

where ω∈(0,1) is a forgetting factor, $\eta^{\left(m\right)}\geq 0$ is a modality-specific learning rate, and $e_{t}^{\left(m\right)}$ is the sufficient-statistics (state-weighted) evidence tensor.

#### Surgical Learning (Lucas-Robust Adaptation)

To prevent endogenous policy feedback from contaminating belief updates, we set

(18) $$\eta^{\left(m\right)}=\left\{\begin{array}{cc} \eta^{\left(m\right)}>0, & m\in M_{\mathrm{learn}},\\ 0, & m\notin M_{\mathrm{learn}}, \end{array}\right.$$

where $M_{\mathrm{learn}}$ denotes the set of observation modalities permitted to adapt via Dirichlet learning. For all agent variants, the P&L likelihood $A^{pnl}$ is held fixed ($\eta^{pnl}=0$) to prevent policy-induced feedback and preserve structural invariance. The specific composition of $M_{\mathrm{learn}}$, ranging from return-only learning to selective multi-modality learning, is agent-dependent and summarized in Table 1.

This design preserves the invariance of endogenous signals under policy changes while retaining adaptive capacity in the presence of regime shifts. In the Professional Agent, learning is restricted to the return likelihood $A^{ret}$ (corresponding to the first observation modality) with learning rate $\eta^{ret}=0.04$ and forgetting factor ω=0.99. All remaining likelihood modalities, including P&L and auxiliary signals, are frozen (η=0) to ensure that belief updating does not conflate investing policy execution with environmental inference (see Appendix A).

### 3.10. Agent Variants (Cognitive Profiles)

We implement three agent variants, denoted *Bearish*, *Neutral*, and *Professional*, respectively corresponding to a highly risk-averse retail investor, a balanced investor with moderate risk tolerance, and an institutional-style investor capable of sustained inference and exploration. These variants share the same generative model structure but differ in (i) preference intensity and loss aversion encoded in C [9], (ii) planning horizon *T* and policy set size |Π|, (iii) epistemic weighting and precision parameters, and (iv) scope of surgical learning (single-modality vs. multi-modality). A step-by-step formalization of this perception--action cycle, including the generative model specification and policy evaluation equations, is provided in Figure 4; the full algorithmic implementation is specified in Algorithm A1.

These profiles are intended to capture qualitatively distinct and economically interpretable decision-making regimes ranging from conservative loss-averse behavior to balanced and professional-grade adaptive inference, rather than to exhaustively span the parameter space. Together, they provide a parsimonious basis for comparing how differing cognitive configurations affect learning, exploration, and performance under identical market dynamics.

#### 3.10.1. Risk-Sensitive Control and Preferences (C-Vector)

Unlike canonical Active Inference, where preferences are encoded as log-prior beliefs over desired observations ($C=\ln P(o^{*})$; [29]), we adopt a *risk-sensitive* formulation in which C represents explicit utilities over outcomes. This modification is essential in financial settings, where gains and losses are valued asymmetrically and relative to a reference point, yielding cardinal utilities that cannot be represented as log-probability ratios without violating normalization or symmetry constraints. Therefore, explicit utility encoding allows coherent treatment of loss aversion, tail risk, and downside sensitivity within the expected free energy (EFE) framework.

Formally, this utility-based preference specification renders policy selection equivalent to KL-regularized control, in which optimal policies are obtained by reweighting uncontrolled dynamics with an exponential utility term. This correspondence was first derived in path-integral control formulations [53] and later formalized explicitly as KL-control and entropy-regularized optimal control [54] while remaining fully consistent with the expected free energy (EFE) objective.

Preferences are applied exclusively to P&L observations, while return observations carry zero utility since the investor is a price taker. This asymmetry enforces the *controllability principle*: preferences attach only to outcomes the agent can influence through action selection, whereas market returns remain exogenous signals used solely for inference. Differences across cognitive profiles arise from the intensity, scaling, and asymmetry of C as well as their interaction with the policy precision γ and planning horizon *T* (Table 1). For all agents, the P&L utility vector $C^{\mathrm{pnl}}$ is constructed from discretized P&L outcome bins, with agent-specific scaling and loss-aversion parameters. Additional detail on the exact constructions for all agents is provided in Appendix A and Appendix C.

Table 1 summarizes the complete architectural specification across cognitive profiles. For each agent, $C^{pnl}$ is constructed by discretizing realized P&L into four outcome bins and applying agent-specific scaling and loss-aversion parameters (see Appendix A and Appendix C for exact construction).

#### 3.10.2. Loss Aversion as a Stability Parameter

Loss aversion λ governs how sharply negative outcomes influence belief-driven action selection [9]. Both extreme and minimal loss asymmetry exacerbate learning instabilities under adaptation, but through distinct mechanisms. In the Bearish Agent, instability arises through excessive defensive belief reinforcement, whereas in the Professional Agent it arises through overconfident regime switching driven by aggressive utility gradients.

The Bearish Agent (λ=1.75) responds to adverse outcomes with amplified defensive updates, reinforcing pessimistic regime beliefs and suppressing exposure even as conditions improve. By contrast, the Professional Agent (λ=1.0) exhibits low asymmetry and higher aggressiveness, which adaptive learning destabilizes through overreaction to short-term fluctuations.

The Neutral Agent (λ=1.50) occupies an intermediate regime in which belief updates remain bounded. This produces a stability basin in which adaptation corrects misalignment between training and deployment regimes rather than amplifying noise. These results indicate that neither extreme caution nor extreme confidence is compatible with adaptive (online) belief updating under endogenous feedback.

#### 3.10.3. Epistemic Weighting and Information Flow

Epistemic weighting controls the relative importance of uncertainty reduction versus expected utility, thereby scaling the sensitivity of belief updates to informational surprise. Higher epistemic weight increases the marginal value of information and accelerates belief revision, consistent with classical results in information economics [55,56].

However, the effect of intensified information-seeking depends critically on the structural origin of the observed signals. When learning is driven by observation channels that are partially policy-dependent, increasing epistemic weight amplifies endogenous feedback, raising posterior variance and accelerating belief drift. This produces a non-monotonic relationship between epistemic drive and out-of-sample performance, consistent with the U-shaped sensitivity observed in the parameter sweeps.

In contrast, when learning is restricted to uncontrollable (structurally invariant) signals, epistemic weighting facilitates rapid correction of regime beliefs by aggregating independent information sources. In this regime, increased epistemic drive improves performance up to a stability boundary.

Thus, epistemic drive enhances performance only when applied to structurally invariant observation channels; beyond a critical threshold, or when applied to policy-contaminated signals, intensified information acquisition accelerates divergence rather than convergence.

#### 3.10.4. Confidence Gating: Uncertainty-Modulated Action Execution

A central challenge in sequential decision-making is determining *when* to act versus when to defer action under uncertainty. In portfolio management contexts, premature execution under diffuse beliefs can induce unnecessary turnover, excess drawdowns, and endogenous feedback loops. We address this problem through *confidence gating*, a mechanism that conditions action execution on posterior certainty.

Actions are executed only when the policy posterior Q(π) satisfies dual thresholds

(19) $$\mathrm{execute}\;⟺\;\left(\max_{\pi }Q\left(\pi \right)\geq p_{\min}\right)\;\wedge \;\left(H\left[Q\left(\pi \right)\right]\leq \phi \cdot H_{\max}\right),$$

where $H\left[Q\left(\pi \right)\right]=-\sum_{\pi }Q\left(\pi \right)\;\ln Q\left(\pi \right)$ is the policy entropy (in nats) and $H_{\max}=\ln\left|\Pi \right|$ is the maximum entropy over the policy space.

The execution gate enforces two complementary requirements. The *confidence threshold* $\max_{\pi }Q\left(\pi \right)\geq p_{\min}$ requires that the most probable investment policy attain sufficient posterior mass, while the *entropy threshold* $H\left[Q\left(\pi \right)\right]\leq \phi \cdot H_{\max}$ ensures that overall uncertainty across the policy space remains bounded. When either condition fails, the agent defaults to holding the current position, effectively abstaining until beliefs consolidate. The Professional Agent uses $p_{\min}=0.22$ and ϕ=0.96, permitting execution only when policy entropy falls below 96% of its theoretical maximum.

This design reflects a core principle of Active Inference, namely, that action precision should scale with posterior confidence [29,57]. Rather than hardcoding risk aversion into preferences, confidence gating allows the agent to endogenously modulate trading intensity based on epistemic state, reducing turnover during regime ambiguity while preserving responsiveness when beliefs are precise.

#### 3.10.5. Theoretical Foundation

Confidence gating follows directly from precision-weighted control in Active Inference, where uncertainty regulates the influence of beliefs on action selection [17,57]. Policy precision acts as a gain control mechanism; when beliefs about future outcomes are uncertain, the impact of expected free energy (EFE) on action selection is attenuated, suppressing commitment. From an information-theoretic perspective, abstention under high uncertainty is rational when actions incur control costs or irreversible consequences [20].

#### 3.10.6. Operationalization

Let Q(π) denote the posterior distribution over candidate policies. Action execution is permitted only when posterior concentration exceeds a minimum confidence threshold, enforced through two complementary criteria:

1. Maximum probability criterion:
  (20) $$\max_{\pi }Q\left(\pi \right)\;\geq \;p_{\min},$$
  ensuring that at least one policy dominates the posterior mass.
2. Entropy criterion:
  (21) $$H\left[Q\left(\pi \right)\right]\;=\;-\sum_{\pi }Q\left(\pi \right)\;\ln Q\left(\pi \right)\;\leq \;H_{\mathrm{gate}},$$
  ensuring global posterior concentration by penalizing diffuse probability mass across the policy space even in cases where a single policy attains high posterior probability.

Execution proceeds only when the following conditions are satisfied:

(22) $${\mathrm{Execute}}_{t}=I\;\left[\max_{\pi }Q\left(\pi \right)\geq p_{\min}\right]\cdot I\;\left[H\left[Q\left(\pi \right)\right]\leq H_{\mathrm{gate}}\right].$$

When the gate is closed, the agent maintains its current position by selecting the Hold action, which is an explicit element of the action space and induces a self-transition in the position state. The complete gating and bounded-execution procedure is formalized in Algorithm A4.

#### 3.10.7. Adaptive Gating

To accommodate time-varying market uncertainty, we implement an adaptive entropy threshold for adaptive agent variants:

(23) $$H_{\mathrm{gate}}\left(t\right)=H_{\mathrm{base}}+\Delta H\cdot H\;\left[A_{t}^{\mathrm{ret}}\right]$$

where $H[A_{t}^{\mathrm{ret}}]$ is the entropy of the return likelihood model at time *t*. When the agent’s observation model is uncertain, the gate tightens, requiring higher confidence before execution. As learning reduces observational uncertainty, the gate relaxes, enabling more frequent action. This creates a principled coupling between epistemic state and behavioral caution. Adaptive gating (Equation (23)) represents an extension for non-stationary environments.

The three agent profiles evaluated in this paper (Table 1) employ fixed entropy thresholds $H_{\mathrm{gate}}=\phi \cdot H_{\max}$ with ϕ∈{0.85,0.90,0.96} to ensure stable and comparable execution behavior across training and out-of-sample evaluation. These values span increasingly permissive execution regimes, from conservative (requiring near-global posterior concentration) to professional-grade deployment that tolerates moderate residual uncertainty while still preventing diffuse and indecisive policies.

#### 3.10.8. Interpretation

Confidence gating implements uncertainty-aware abstention: the agent acts only when it knows enough to justify investing. Unlike heuristic trading filters [58], this mechanism emerges naturally from Active Inference [59] and bounded-rational control theory [60]. It complements volatility-based position scaling by regulating *decision frequency* rather than position size, yielding a robust form of endogenous risk management that mitigates overtrading under regime ambiguity and distributional shift.

### 3.11. Execution Layer and Risk Controls

The POMDP agent selects discrete actions/policies; an execution layer maps these decisions to tradeable portfolio weights and enforces realistic constraints.

#### 3.11.1. Position Mapping

Latent position states map to target weights:

(24) $$w\left(s^{p}\right)\in \left\{-w_{\max},\;0,\;+w_{\max}\right\},$$

where $w_{\max}\in \left\{0.5,1.0\right\}$ depends on the agent variant.

#### 3.11.2. Volatility Scaling and Execution Constraints

Position adjustments are scaled by realized volatility to maintain approximately constant risk exposure across regimes, a standard practice in volatility targeting and risk parity strategies [61,62,63]. Specifically, effective position changes are computed as

(25) $$\Delta w_{\mathrm{eff}}\;=\;\Delta w\cdot \mathrm{clip}\;\left(\frac{\sigma_{\mathrm{target}}}{{\hat{\sigma }}_{t}},\;0,\;1.0\right),$$

where ${\hat{\sigma }}_{t}$ is the 20-day realized volatility estimate and $\sigma_{\mathrm{target}}$ is the agent-specific risk target. The 20-day window corresponds approximately to one trading month, balancing responsiveness to regime shifts against robustness to transient noise, and is standard in empirical volatility targeting and portfolio risk management practice.

To ensure execution realism and avoid excessive trading behavior, we impose the following constraints:

1. Minimum-change threshold ($\left|\Delta w\right|\geq \delta_{\min}$), which suppresses micro-trading under noisy belief fluctuations [64].
2. Maximum step size ($|\Delta w_{\mathrm{eff}}|\leq \delta_{\max}$), which limits abrupt exposure shifts that can amplify financial system-level risk through nonlinear feedback (i.e., contagion effects) in interconnected financial systems [65].
3. Flat-on-flip constraint, requiring sign reversals to pass through zero exposure, thereby simulating institutional risk controls, margin constraints, and position unwinding practices [66].

These constraints operationalize risk-aware execution while preserving the agent’s ability to adapt positions in response to inferred regime changes.

#### 3.11.3. Transaction Costs and Strategy Returns

We model trading frictions using proportional transaction costs

(26) $${\mathrm{TC}}_{t}=c\cdot \left|w_{t}-w_{t-1}\right|,\;c=5\;\mathrm{bps},$$

consistent with empirical estimates for liquid U.S. equity ETFs under moderate turnover [67,68]. This specification captures bid–ask spreads, market impact, and execution slippage in reduced form.

Our baseline transaction cost assumption of five basis points per trade is directly supported by empirical evidence. In [68], the report realized ETF trading costs of 4.72 bps for S&P 500 index funds using live fund data. Given ARKK’s substantially higher turnover, volatility, and exposure to less liquid securities, our assumption is conservative.

Strategy returns are computed as

(27) $$r_{t}^{\mathrm{strat}}=w_{t-1}\;r_{t}^{\mathrm{mkt}}-{\mathrm{TC}}_{t},$$

ensuring that costs are charged on position changes rather than notional exposure. Using lagged portfolio weights avoids look-ahead bias and reflects execution at the beginning of the return interval. This convention aligns with standard backtesting practice in portfolio management and avoids artificially inflating performance under frequent rebalancing [69].

#### 3.11.4. Decision Cadence

Belief updating and potential execution occur every k=5 trading days, with portfolio weights held constant between decision points. This weekly cadence reflects institutional rebalancing frequencies commonly observed in discretionary and systematic asset management [70,71]. From a modeling perspective, a five-day cadence also reduces sensitivity to microstructure noise and short-horizon return reversals, allowing belief updates to reflect persistent regime information rather than transient price fluctuations.

From a theoretical perspective, infrequent action mitigates endogenous feedback between policy adaptation and observations, reducing overfitting and excessive turnover under model uncertainty [14,72]. Within Active Inference, coarser decision intervals also reduce control costs associated with frequent action under uncertain beliefs, aligning with bounded rationality and information-processing constraints [20].

### 3.12. Experimental Protocol and Evaluation

#### 3.12.1. Asset Choice and Stress-Test Logic

We evaluate Adaptive Inference on the ARK Innovation ETF (ARKK) because it provides an economically meaningful stress test for sequential decision-making under non-stationarity. ARKK is an actively managed thematic equity ETF focused on disruptive innovation, with a mandate that concentrates exposure in high-growth, high-duration firms and consequently induces large swings in volatility, drawdowns, and recovery dynamics over a single decade of liquid daily data [73]. These features create (i) pronounced regime transitions, (ii) persistent distributional change between training and deployment, and (iii) realistic endogenous feedback between allocation decisions and portfolio outcomes.

From a modeling standpoint, ARKK constitutes an informative *stress test case*, since it is a high-volatility asset characterized by frequent regime shifts, time-varying signal-to-noise ratios, and pronounced drawdown–recovery cycles. If an inference-guided controller cannot maintain calibration and stability under such conditions, it is unlikely to be robust in more benign market environments. Conversely, any stability advantage of frozen transfer (the Passivity Paradox) is most visible precisely when markets exhibit large state transitions and rapidly changing informational conditions. Daily adjusted close prices for the ARKK Innovation ETF (ARKK), along with auxiliary market variables (e.g., volume), were obtained from Yahoo Finance via the [74] API.

#### 3.12.2. Risk-Free Rate Construction

All reported Sharpe/Sortino ratios and statistical tests are computed on *risk-free-adjusted* daily excess returns. As the risk-free proxy, we use the three-month U.S. Treasury Bill rate ($r_{t}^{f}$; T-Bill), a standard short-horizon benchmark in empirical asset pricing and performance evaluation. The risk-free rate is proxied by the daily three-month U.S. Treasury Bill yield [75] and aligned exactly to ARKK trading days.

Let $y_{t}$ denote the annualized T-Bill yield (in decimals). We convert $y_{t}$ to a daily simple risk-free return $r_{t}^{f}$ using a frequency-consistent mapping (e.g., $r_{t}^{f}=y_{t}/252$ as a transparent approximation, or alternatively $r_{t}^{f}={\left(1+y_{t}\right)}^{1/252}-1$ for compounding). Daily strategy excess returns are then defined as

$$x_{t}\;=\;r_{t}^{\mathrm{strategy}}-r_{t}^{f},$$

and all mean-dominance tests and HAC/DM procedures operate on ${\left\{x_{t}\right\}}_{t=1}^{T}$.

#### 3.12.3. Trading-Day Alignment (No Calendar Assumptions)

Because the strategies trade on the ETF’s realized trading calendar, we align the risk-free series to the exact ARKK trading-day index (train and test separately) via reindexing and forward-filling between quoted observations. This follows best practices in empirical asset pricing and backtesting, where returns and discount rates must be matched on identical observation dates to avoid spurious excess-return calculations induced by calendar mismatches [69,76]. By avoiding business-day heuristics and assumed calendars, this procedure ensures a one-to-one correspondence between each realized portfolio return and the contemporaneous risk-free rate used to compute excess returns. The same alignment convention is applied to both the agent P&L series and the Buy-and-Hold benchmark, guaranteeing strict comparability across all reported performance metrics.

#### 3.12.4. Interpretation

Using a short-maturity Treasury benchmark matters economically in our sample because the post-2022 test window features a major historical bull market compared to the training window. Excess-return accounting prevents mechanical overstatement of performance during high-rate periods and makes frozen vs. adaptive comparisons invariant to changes in the outside option of holding cash.

#### 3.12.5. Train–Test Split

We train all model parameters on 2015–2022 and evaluate out-of-sample on 2022–2025 under identical data preprocessing.

##### Learning Modes

We compare:

- Frozen (inference-only) transfer: All learning disabled out-of-sample ($\eta^{\left(m\right)}=0$ for all *m*), no-adaptation/no-update regime.
- Adaptive (online learning): Surgical learning enabled out-of-sample with agent-specific modality masks, following Equation (18) and Table 1.

#### 3.12.6. Metrics and Inference

We report standard risk-adjusted performance measures including the Sharpe ratio, Sortino ratio, maximum drawdown (MaxDD), and compound annual growth rate (CAGR), all computed on daily risk-free, rate-aware excess returns. Statistical uncertainty is quantified using bootstrap confidence intervals and robust Sharpe comparisons [77,78].

Standard performance metrics evaluate return per unit of financial risk, but are agnostic to the informational effort required to generate those returns. In adaptive systems, however, returns are produced through belief updating and regime inference, processes that consume informational resources and may amplify noise when overactive.

To capture this dimension, we introduce the *Entropic Sharpe Ratio (ESR)*, defined as the excess return per unit of entropy reduction induced by belief updates. Therefore, the ESR measures how efficiently the agent converts informational work into financial performance.

This metric operationalizes the information–performance tradeoff central to rational inattention and information-constrained control [10,20,34]. A strategy that achieves high returns through excessive belief volatility or overupdating will exhibit a lower ESR, whereas one that achieves comparable returns with parsimonious and stable inference will exhibit a higher ESR. As such, ESR distinguishes financial efficiency from cognitive efficiency and provides a diagnostic for overactive learning dynamics.

#### 3.12.7. Cross-Asset Robustness Check

To distinguish asset-specific effects from methodological artifacts, we evaluate the robustness of the inference architecture under a change in market environment by applying the same Professional Agent design to the S&P 500 ETF (SPY). SPY provides a complementary test case, whereas ARKK exhibits high volatility and pronounced regime structure; SPY features lower volatility, weaker regime separation, and more stable return dynamics.

For this experiment, we hold the cognitive architecture fixed across assets, including the policy space, transition dynamics, preferences, priors, execution rules, and risk-aware accounting. The only asset-specific component is the return observation likelihood $A^{\left(0\right)}$, which is re-instantiated for SPY solely to express the same latent return states in the natural scale of SPY returns (i.e., to remap state bins to the SPY return distribution) without altering the number of states, their semantics, or any learning or decision mechanism. All remaining model components are identical to the ARKK configuration. We then compare frozen versus adaptive learning modes out-of-sample on SPY using the same evaluation window and performance metrics.

This design isolates the effect of diminished regime informativeness on the value of endogenous learning, without conflating architectural robustness with asset-specific return scaling or discretization artifacts. Results are reported in Section 4.3, with full validation diagnostics provided in Appendix B.7.

### 3.13. Benchmark Models

To contextualize the performance of Adaptive Inference, we evaluate two classical valuation-based benchmarks that represent standard approaches in financial economics: a Black–Scholes–Merton (BSM) Real Options strategy, and a Stochastic Discounted Cash Flow (DCF) model with Bayesian state estimation. Both benchmarks are implemented under identical execution constraints, risk-free conventions, transaction costs, and train–test separation as the Adaptive Inference agents, ensuring a fair, auditable, and economically meaningful comparison.

#### 3.13.1. Black–Scholes–Merton (BSM) Real Options

The BSM Real Options benchmark interprets portfolio allocation as a sequence of real-option decisions [42,43,79,80]. At each decision date, the model computes the risk-neutral probability of a positive payoff using the BSM framework, with the underlying asset price as the spot variable, an exponentially weighted moving average as the strike anchor, and realized volatility estimated from rolling returns. Portfolio positions are determined by thresholding this probability around indifference, yielding long, short, or flat exposures.

The BSM Real Options model benchmark represents a classical valuation paradigm for contingent claims in which volatility is treated as known, regime uncertainty is suppressed, and epistemic value is absent. In the language of Active Inference, it corresponds to a limiting case where beliefs are fixed and the expected free energy (EFE) reduces to the expected utility under risk-neutral valuation (see Appendix G for full details).

#### 3.13.2. Stochastic Discounted Cash Flow (DCF)

The Stochastic DCF benchmark estimates fair asset value as the discounted expectation of future cash flows over a finite horizon [44,81]. Cash-flow growth is modeled as a latent stochastic process inferred via a Bayesian dynamic linear model (Kalman filter) [82,83], with parameters estimated exclusively on the training window.

Future cash flows are simulated using Monte Carlo methods, and the terminal value is computed via a Gordon growth approximation [84]. Portfolio signals are generated from deviations between the estimated fair value and the observed market price. This benchmark captures a passive expectations paradigm in which beliefs are formed during training and held fixed at evaluation time, with no endogenous interaction between actions and belief updating. In Active Inference terms, it represents a limiting case where epistemic control is suppressed and valuation reduces to discounted expected cash flows under fixed beliefs (see Appendix G for full details).

#### 3.13.3. Hedge (Multiplicative Weights) Algorithm

To complement valuation-based benchmarks with a modern adaptive baseline, we introduce a third comparator drawn from online learning theory: the Hedge algorithm [23], also known as the multiplicative weights update method [24]. Unlike the BSM and Stochastic DCF, which encode specific valuation paradigms, Hedge is a model-free, adaptive decision algorithm equipped with worst-case performance guarantees.

Hedge maintains a panel of *K* trading strategies (“experts”) that each propose a target position at every decision date. The algorithm keeps a probability distribution—a simplex of weights—over these experts, which is initially uniform. After each decision window, it observes the realized net payoff of every expert (including those not followed) and multiplicatively reweights them in proportion to performance: stronger experts receive exponentially higher weight, while weaker experts are downweighted. The executed portfolio is the blended target under the same friction and risk layer as the Adaptive Inference agent. The cumulative regret relative to the best expert in hindsight is bounded by $O(\sqrt{T\ln K})$, a guarantee that holds under adversarial payoff sequences without distributional assumptions [85].

This adversarial no-regret property distinguishes Hedge from heuristic strategy selection rules. The guarantee is nonparametric and does not require stationarity, ergodicity, or a specific return-generating process, making it well-suited to nonstationary financial environments [86]. Applications of multiplicative updates to portfolio selection were formalized by Helmbold et al. [87], who showed that such algorithms achieve no-regret performance relative to individual assets and compete favorably with universal portfolio constructions [88] on long-horizon equity data.

We implement Hedge over a panel of eight classical strategies spanning passive, momentum, mean-reversion, trend-following, volatility-responsive, and short-momentum styles, ensuring broad coverage of the strategy space. All experts are executed through an identical friction and risk layer as the Professional Agent, with the same transaction costs, decision cadence, volatility scaling, position bounds, and flat-on-flip constraints, ensuring that performance differences reflect decision architecture rather than execution asymmetry. The learning rate is set by the minimax formula $\eta^{*}=\sqrt{8\ln K/(c^{2}T)}$, with no tuning to the test data. Full construction details, a post hoc invariance audit, sensitivity analysis, and diagnostics are provided in Appendix H.

Deep reinforcement learning approaches (e.g., PPO, SAC) represent another class of modern adaptive methods. However, they typically require extensive hyperparameter selection, architectural design choices, and stochastic training procedures that complicate reproducibility and theoretical interpretability in non-stationary financial settings [89]. By contrast, Hedge provides a transparent, theoretically grounded, and model-free adaptive benchmark with explicit finite-sample regret guarantees under execution parity.

Thus, the Hedge benchmark serves as a limiting-case test of whether Adaptive Inference’s structural properties of explicit belief states, epistemic exploration via expected free energy (EFE), and ante hoc decision decomposability can provide advantages beyond those achievable by any no-regret convex combination of classical strategies operating without a generative model of market regimes.

### 3.14. Execution and Evaluation Parity

All benchmark strategies are evaluated using the same execution layer as the Adaptive Inference agents, including weekly decision cadence, volatility-based position scaling, transaction costs, through-flat constraints on sign changes, and risk-free–adjusted excess returns. This ensures that all observed performance differences arise from inference and valuation structure rather than execution asymmetries.

## 4. Results

We organize the results around four questions:

1. How do Adaptive Inference agents perform relative to passive benchmarks? (Section 4.1)
2. Does the Passivity Paradox hold, and how does cognitive architecture moderate it? (Section 4.2 and Section 4.3)
3. Does the framework generalize across assets? (Section 4.3)
4. What mechanisms explain performance through the Entropic Sharpe Ratio and statistical validation? (Section 4.4 and Section 4.5)

Throughout, we emphasize the Professional Agent as our primary specification while reporting comparative results across all cognitive profiles.

This section reports out-of-sample evidence from our Adaptive Inference portfolio framework on ARKK Innovation ETF and establishes two important findings. First, we document the *Passivity Paradox*: for single-modality agents, freezing trained parameters dominates adaptive online learning at deployment. In our primary specification (Professional; λ=1.0), the Frozen Agent achieves a positive Sharpe ratio of +0.39, whereas its Adaptive counterpart deteriorates to −0.28—a gap of +0.67 Sharpe units, consistent with maladaptive belief drift under endogenous feedback. Second, we identify a sharp boundary condition: the paradox *reverses* under multi-modality surgical learning. The Neutral Agent (λ=1.5) improves by +1.69 Sharpe points under adaptation and achieves the lowest drawdown in the study (MaxDD=−8.8%), indicating that learning is beneficial when restricted to exogenous information channels.

### 4.1. Aggregate Performance

We begin by evaluating aggregate out-of-sample performance across all cognitive profiles and learning modes (Frozen transfer vs. Adaptive online updating). Table 2 reports standard risk-adjusted metrics for each Adaptive Inference agent relative to the Buy-and-Hold benchmark over the strictly out-of-sample test period (25 August 2022–5 December 2025; n=824 trading days). This window follows immediately after the training endpoint and encompasses a volatile post-drawdown recovery with multiple shifts in market conditions, including a significant bull market, thereby providing a stringent assessment of belief transfer, learning stability, and regime adaptation under distributional change.

**Proposition** **2** (The Passivity Paradox)**.** *In multi-period portfolio management problems with endogenous feedback between actions and observations, freezing learned belief parameters at deployment can dominate online adaptive updating when learning is driven by a single observation modality that is partially policy-dependent. In such settings, adaptive learning introduces belief drift. This drift degrades regime inference and reduces risk-adjusted performance. Conversely, when learning is surgically restricted to observation modalities that are exogenous to the agent’s control, adaptive updating can improve out-of-sample performance. In this case, learning corrects the misalignment between training and deployment regimes. Thus, the performance impact of adaptation depends critically on what the agent learns. Adaptation is beneficial only when learning targets uncontrollable environmental signals rather than policy-contingent outcomes.*

The proposition identifies a boundary condition for successful adaptation in financial decision-making. Learning is beneficial only when it is confined to observation channels that preserve structural invariance under policy changes. This condition is violated by naive online learning but satisfied by multi-modality surgical learning.

#### 4.1.1. Benchmark Comparison

To situate Adaptive Inference against both canonical valuation-based finance and modern adaptive methods, we evaluate three benchmarks under identical execution, transaction cost, and risk-free-adjusted evaluation conventions. Specifically, all benchmark returns are computed as daily risk-free, rate-aware excess returns, net of 5 bps transaction costs [67,68], and subject to the same rebalancing cadence and execution constraints used throughout the paper (cf. Appendix A and Appendix D). This alignment ensures that performance differences reflect model structure rather than trading mechanics.

Table 3 reports out-of-sample performance for the test period (25 August 2022–5 December 2025) under these unified conventions. The BSM benchmark serves as a limiting-case proxy for valuation under known volatility and collapsed uncertainty, the Stochastic DCF benchmark serves as a limiting-case proxy for fixed beliefs and passive expectations, and the Hedge algorithm benchmark [23,24] tests whether provably adaptive no-regret aggregation of classical strategies without an explicit generative model of latent regimes can match inference-guided control. Full specifications are provided in Appendix G.1, Appendix G.2 and Appendix H.

Three empirical conclusions follow. First, under identical execution and risk-free-excess accounting, both valuation-driven benchmarks underperform materially out-of-sample on ARKK: the BSM Real Options strategy yields a negative Sharpe ratio of (−0.16) with deeper drawdowns than Buy-and-Hold, and the Stochastic DCF benchmark deteriorates even further (Sharpe ratio =−0.52, MaxDD =−70.7%), consistent with valuation instability on growth assets and regime nonstationarity (Appendix G.2).

Second, the Hedge benchmark—a provably adaptive online learning algorithm with worst-case regret guarantees—achieves a Sharpe ratio of  +0.40 with the lowest maximum drawdown in the study (−26.0%), confirming that model-free adaptive strategy combination can achieve competitive risk-adjusted returns, particularly through drawdown mitigation.

Importantly, Hedge’s performance is stable across learning-rate choices and degrades monotonically under higher transaction costs (Appendix H), indicating that results are not driven by hyperparameter tuning.

Third, the Professional Frozen Agent achieves comparable risk-adjusted performance to Hedge (Sharpe ratio of +0.39 vs. +0.40) while dominating both classical benchmarks, but does so through an explicit generative model that provides belief-state decomposition, epistemic uncertainty quantification, and ante hoc decision auditability, which are structural properties absent from all three benchmarks.

Notably, the drawdown gap between Hedge and Professional Frozen Agent (−26.0% vs. −35.8%) reflects differing risk allocation structures, whereas the comparable Sharpe ratios indicate that belief-guided control does not sacrifice performance relative to adversarially robust online learning.

This extended panel supports the central claim that Adaptive Inference yields not only competitive performance but qualitatively superior interpretability and regulatory alignment in regime-rich non-stationary environments.

##### Core Finding: Frozen Agent Deployment Delivers Economically Meaningful Risk Control

Across profiles, Adaptive Inference agents systematically reduce volatility and drawdowns relative to Buy-and-Hold, even when underperforming on raw returns. The Professional Frozen Agent (λ=1.0) achieves a Sharpe ratio of +0.39 with a maximum drawdown of −35.8%, improving drawdown protection by 3.8 percentage points relative to Buy-and-Hold (−39.6%) while capturing approximately 60% of benchmark CAGR (12.3% vs. 20.5%). This establishes Adaptive Inference as a *risk-shaping* rather than return-maximizing framework. Value arises through controlled exposure aligned with inferred regimes, not through aggressive return chasing.

**Figure 7:**
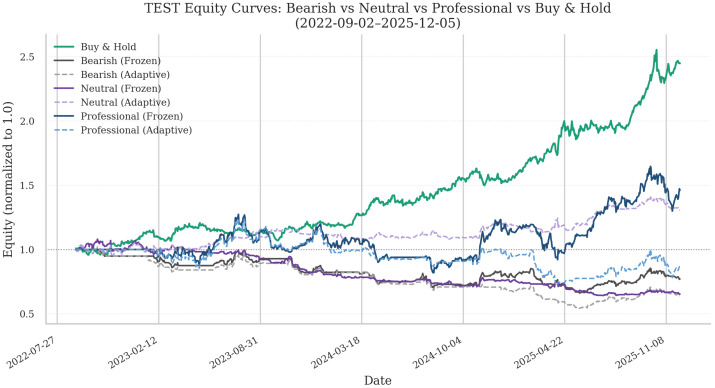
Out-of-Sample Equity Curves on ARKK: Cognitive Profiles and Learning Modes. Shown are cumulative equity curves (normalized to 1.0 at test start) over the out-of-sample period (2 September 2022–5 December 2025). Buy-and-Hold ARKK (green) achieves the highest terminal return but exhibits large volatility and deep drawdowns. For single-modality agents, adaptive learning (dashed lines) systematically underperforms frozen deployment (solid lines), most notably for the Professional profile (dark blue). The Professional Frozen Agent tracks the benchmark during rallies with reduced amplitude, delivering materially lower drawdowns at the cost of foregone upside. In contrast, the Neutral Agent (purple) reverses the paradox: adaptive learning improves performance and stabilizes equity relative to its frozen counterpart, consistent with successful surgical learning on exogenous modalities. Bearish Agents (gray) underperform across modes, reflecting persistent defensive positioning (higher loss aversion).

Figure 7 displays cumulative equity paths for the three agents (Bearish, Neutral, and Professional), illustrating their divergence patterns versus the Buy-and-Hold strategy.

**Figure 8:**
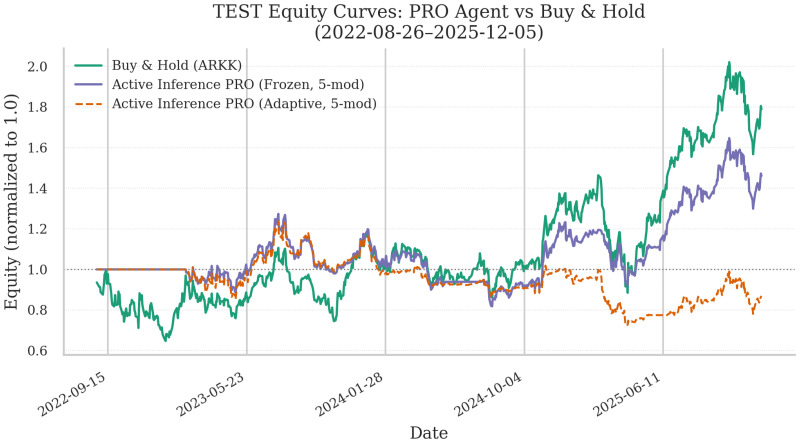
Cumulative equity curves for the Professional Agent on ARKK (out-of-sample). Cumulative equity (normalized to 1.0 at test start) for Buy-and-Hold ARKK (green), Professional Frozen Agent (solid purple ), and Professional Adaptive Agent (orange dashed) over the out-of-sample period (26 August 2022–5 December 2025). The Frozen Agent tracks the benchmark during market recoveries while exhibiting reduced drawdown amplitude and lower volatility, achieving a positive Sharpe ratio. In contrast, the Adaptive Agent diverges negatively from mid-2024 onward, entering a persistent drawdown despite increased trading activity, illustrating the Passivity Paradox for single-modality agents.

Figure 8 reports the out-of-sample equity curve for the Professional Agent on ARKK, contrasting Frozen and Adaptive deployment against Buy-and-Hold.

**Figure 9:**
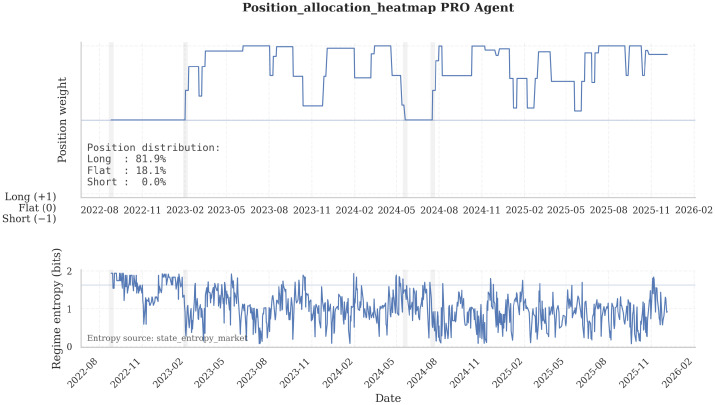
Position allocation and regime-entropy dynamics for the Professional Frozen Agent (ARKK, out-of-sample). The top panel displays the discrete portfolio position selected by the Professional Frozen Agent (Long = +1, Flat = 0, Short = −1). Grey shaded bands mark episodes where posterior regime entropy exceeds the high-uncertainty threshold (horizontal grey line, bottom panel), triggering the confidence-gating mechanism that reduces exposure. The agent maintains long exposure for 81.9% of evaluation days but intermittently shifts to a flat allocation during specific episodes. The bottom panel reports the posterior entropy (in bits) of the inferred market regime. Periods of elevated entropy coincide with flat positioning, while sustained long exposure occurs predominantly during lower-entropy regimes. The figure illustrates the agent’s regime-conditioned exposure adjustments: portfolio risk is reduced during high-uncertainty intervals and maintained when regime beliefs are more concentrated. These diagnostics provide a behavioral explanation for the agent’s risk-shaping relative to Buy-and-Hold, linking exposure modulation to inferred regime uncertainty rather than static allocation.

Figure 9 complements this with the corresponding exposure dynamics, which clarify how deployment learning changes trading behavior.

#### 4.1.2. Adaptive Learning Degrades Aggregate Performance Except Under Surgical Conditions

For both the Bearish and Professional profiles, adaptive learning uniformly worsens performance. The Professional Adaptive Agent exhibits a Sharpe ratio of −0.28 and a negative CAGR of −4.4% despite the higher execution frequency, confirming that online belief updates introduce instability rather than adaptability in single-modal architectures. In contrast, the Neutral Agent exhibits a qualitatively different pattern: adaptive learning improves Sharpe by +1.69 and reduces maximum drawdown to −8.8%, the lowest in all configurations.

#### 4.1.3. Volatility Reduction Is Universal, While Upside Capture Is Profile-Dependent

All Adaptive Inference agents reduce realized volatility relative to ARKK Buy-and-Hold (43.0% annualized). The Professional Frozen Agent reduces volatility to 28.4%, while the Neutral Adaptive Agent achieves the most defensive profile with volatility of 13.2%. Upside participation varies sharply by cognitive architecture, however: the Professional Frozen Agent maintains 65.0% long exposure in the latent belief state, aligning with the recovery regime, whereas the Neutral Frozen Agent remains underexposed (21.2%) until adaptive learning corrects this misalignment.

### 4.2. Empirical Evidence of the Passivity Paradox

Aggregate performance statistics establish the existence of the Passivity Paradox, but do not by themselves explain its origin. In this subsection, we isolate the mechanism underlying the paradox by examining how adaptive learning alters agents’ internal generative models during deployment. We show that for single-modality agents, online updating induces systematic belief drift; as the agent continuously re-fits its model to short-horizon observations, its latent regime beliefs become progressively misaligned with the true market structure, leading to degraded state inference, unstable action selection, and inferior risk-adjusted performance. By contrast, multi-modality surgical learning constrains belief updates to exogenous information channels, preventing such drift and enabling productive adaptation.

#### 4.2.1. Belief Drift as the Central Mechanism

Adaptive Inference Agents update parameters of the likelihood mapping A-matrix during deployment. For Frozen Agents, these parameters remain fixed at their training terminal values. To quantify the magnitude of belief change induced by adaptive (online) learning, we compute the Frobenius norm of the difference between the post-deployment and training likelihood matrices:

$$∥\Delta A^{\left(r\right)}{∥}_{F}\;=\;∥A_{\mathrm{test},\mathrm{end}}^{\left(r\right)}-A_{\mathrm{train},\mathrm{end}}^{\left(r\right)}{∥}_{F},$$

where $A^{\left(r\right)}$ denotes the return-observation likelihood governing inference of regime detection.

Table 4 reports belief drift alongside performance differentials for each cognitive profile.

Two patterns are immediate. First, belief drift is largest for the Professional and Bearish Agents, precisely the profiles for which adaptive learning degrades performance. Second, the Neutral Agent exhibits substantially smaller drift and simultaneously reverses the paradox, achieving both higher Sharpe ratios and materially reduced drawdowns under adaptation.

#### 4.2.2. Maladaptive Learning in Single-Modality Agents

For the Professional Agent, adaptive learning produces the largest likelihood distortion ($∥\Delta A^{\left(r\right)}{∥}_{F}=1.139$). Inspection of the learned return-likelihood matrices $A^{\left(r\right)}$ reveals that adaptive updates systematically reassign policy-induced P&L variation to latent regime transitions. This constitutes a direct violation of structural invariance: the agent learns from observations for which the distribution depends on its policy. As a result, regime beliefs become increasingly misaligned with the true environmental dynamics, leading to unstable positioning and persistent drawdowns. Structural invariance here refers to the assumption that the likelihood mapping A-matrix remains invariant under changes in the chosen policy, an assumption that is violated when observations depend on actions.

The Bearish Agent exhibits the same mechanism, though at slightly lower magnitude. Elevated loss aversion amplifies defensive reactions to belief updates, reinforcing pessimistic regime inference and locking the agent into persistently conservative position sizing (i.e., underexposed position states). In both cases, adaptive learning creates a self-reinforcing feedback loop in which belief updates respond to endogenous outcomes rather than exogenous signals, a portfolio-level manifestation of the Lucas Critique.

#### 4.2.3. Why the Neutral Agent Escapes the Paradox

The Neutral Agent represents a sharp boundary case. Although it also adapts online, its learning protocol is surgically restricted to multiple observation modalities—returns, momentum, volatility, and volume—that are exogenous to portfolio actions. As a result, belief updates remain structurally invariant to changes in the agent’s own policy and executed positions, that is, the likelihood mappings being updated do not change when the agent intervenes through its portfolio choices. The observed likelihood drift is modest ($∥\Delta A^{\left(r\right)}{∥}_{F}=0.240$) despite ongoing adaptation, reflecting that learning occurs in observation channels orthogonal to portfolio control.

This contrast supports that the Passivity Paradox does not arise from learning per se, but from *where* learning is applied. When belief updates target policy-dependent channels, adaptation degrades performance; when restricted to uncontrollable environmental signals, adaptation improves it.

#### 4.2.4. Persistent Drawdown Under Belief Corruption

**Figure 10:**
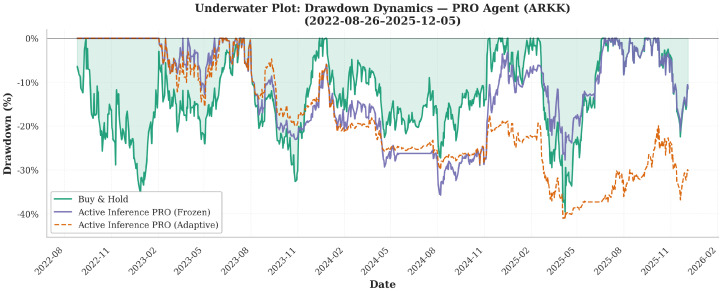
Professional Agent Drawdown Dynamics. Percentage below cumulative peak equity during out-of-sample period (26 August 2022–5 December 2025). Buy-and-Hold (green) reaches maximum drawdown of −39.6%. The Professional Frozen Agent (purple) maintains shallower drawdowns throughout, with a maximum −35.8%. The Professional Adaptive Agent (orange dashed) diverges negatively from mid-2024, entering a persistent deep drawdown (−41.3%) that fails to recover, providing visual evidence of the maladaptive learning spiral in which endogenous feedback corrupts regime beliefs. The Professional Adaptive Agent shows an extended period of underperformance relative to the Professional Frozen Agent and Buy-and-Hold.

Figure 10 illustrates the cumulative impact of belief drift for the Professional Agent. While the Frozen Agent experiences episodic drawdowns followed by recovery, the Adaptive Agent enters a prolonged underwater period beginning in mid-2024 and fails to regain prior equity peaks. This persistence is consistent with corrupted regime beliefs, meaning that the agent’s inferred market regime states become systematically misaligned with the true underlying return dynamics due to learning from policy-dependent signals. As a result, portfolio decisions are conditioned on distorted state estimates rather than exogenous market information, producing sustained mispositioning rather than transient losses. This pattern reinforces the interpretation of adaptive learning rather than adverse market shocks as the causal driver of underperformance.

Together, these results establish policy-induced belief drift as the operative mechanism behind the Passivity Paradox, with initial evidence that the mechanism persists across distinct equity volatility regimes as well as across risk-adjusted metrics.

#### 4.2.5. Behavioral Robustness of the Passivity Paradox

To evaluate whether the Passivity Paradox reflects a fragile calibration artifact or a structural property of adaptive inference, we conduct a behavioral parameter sweep across three orthogonal dimensions of the agent’s cognitive architecture: policy precision (γ), epistemic weight (η), and loss aversion (λ). Across 18 total configurations, the paradox holds in 15 cases (83.3%), with the three violations occurring only at economically interpretable boundary conditions (decision viability, performance convergence, and preference destruction).

Policy precision (γ): Decision viability and non-monotonicity. The paradox holds strongly within an operationally relevant regime of policy precision. For γ∈[5,8], the Frozen Professional Agent consistently outperforms its Adaptive counterpart, with ΔSharpe ranging from +0.22 to +0.67. At γ=3, the performance ordering reverses (ΔSharpe =−0.42), not due to adaptive superiority but to decision paralysis in the Frozen Agent, which executes fewer than 4% of decision opportunities. This regime lies below a minimal viability threshold for policy precision, and as such defines a structural boundary condition rather than a counterexample to the paradox. At higher precisions, performance exhibits a non-monotonic structure: at γ=10, both agents converge near zero Sharpe (a narrow neutrality notch), while at γ=15 the paradox re-emerges at its strongest (ΔSharpe =+0.75), consistent with the hypothesis that excessive precision amplifies the costs of endogenous belief corruption under adaptive learning.

Epistemic weight (η): Epistemic value stabilizes frozen beliefs. The epistemic weight sweep further strengthens the mechanism interpretation. Across all tested values η∈[0,1.0], the Frozen Agent strictly dominates the Adaptive Agent, with uniformly positive ΔSharpe (six out of six cases), including η=0, where epistemic value is entirely absent. Moreover, the Sharpe ratio of the Frozen Agent improves broadly with η, increasing from +0.01 at η=0 to +0.39 at η=1.0, indicating that the epistemic value acts as a stabilizing *regularizer* when beliefs remain fixed. In contrast, the Adaptive Agent’s Sharpe ratio remains negative throughout the sweep, confirming that the paradox does not arise from epistemic objectives per se but from updating likelihood mappings that are endogenously entangled with the agent’s own actions.

Loss aversion (λ): Preference robustness and a preference-destruction boundary. The loss-aversion sweep completes the robustness assessment by varying the parameter that shapes *what the agent wants*, rather than how decisively it acts or how strongly it values information. Loss aversion λ scales the penalty assigned to loss outcomes in the PnL preference vector $C^{\left(\mathrm{PnL}\right)}$, directly modulating the agent’s asymmetric sensitivity to gains versus losses in the spirit of Kahneman–Tversky prospect theory [9]. The paradox holds across six of seven tested configurations (λ∈{0.75,1.00,1.25,1.50,1.75,2.25}), with ΔSharpe ratios ranging from +0.06 to +0.72. At low loss aversion (λ=0.75), the Frozen Agent maintains 73% long exposure and trades only 37 times over the out-of-sample period, capturing the equity risk premium through disciplined inaction. As λ increases, both agents progressively retreat toward flatter allocations and trade more frequently, narrowing the performance gap. The single reversal at λ=2.00 (ΔSharpe =−0.54) occurs at a preference-destruction boundary where loss penalties exceed twice the gain rewards ($C^{\left(\mathrm{PnL}\right)}=\left[+4.0,+1.2,-2.4,-8.0\right]$), inducing reactive thrashing under frozen beliefs while the Adaptive Agent’s belief drift partially dampens the extreme preference signal. Consistent with the Lucas Critique mechanism, adaptive belief drift increases with loss aversion, with $∥\Delta A^{\left(0\right)}∥$ rising from 1.01 at λ=0.75 to 1.40 at λ=1.75, indicating that stronger loss preferences amplify endogenous belief corruption.

Taken together, these sweeps span orthogonal dimensions of the agent’s cognitive architecture—how decisively it acts (γ), how much it values information (η), and how asymmetrically it penalizes losses (λ). Thus, the Passivity Paradox emerges not as a universal rule but as a conditional structural property of adaptive inference systems: whenever likelihood updating operates on policy-dependent signals within a viable decision regime, frozen belief deployment systematically dominates endogenous adaptation. The paradox holds across 15 of 18 tested configurations (83.3%), with the three violations occurring only at the interpretable boundary conditions of decision paralysis (γ=3), performance convergence (γ=10), and preference destruction (λ=2.00).

#### 4.2.6. Planning Depth and Policy Entropy

Planning horizon and policy space size further moderate learning stability by shaping the entropy of the policy posterior and the effective control complexity faced by the agent [20,31]. The Professional Agent employs the deepest planning horizon and the largest policy set, resulting in high policy entropy and strong sensitivity of expected free energy (EFE) gradients to belief changes. High policy entropy amplifies the sensitivity of the policy posterior to small belief perturbations, meaning that minor inference errors induce large shifts in posterior mass across competing policies. When translated into execution, these posterior shifts manifest as large changes in selected actions. Under adaptive learning, small distortions in regime beliefs propagate rapidly through the policy posterior, producing unstable action selection.

By contrast, the Neutral Agent’s shorter planning horizon limits compounding effects across future steps, reducing the amplification of belief noise. This architectural constraint dampens feedback loops between inference and control, allowing adaptive learning to operate without destabilizing downstream decisions.

### 4.3. Cross-Asset Robustness: Validation on Low-Volatility Markets

To assess whether the Passivity Paradox reflects a structural property of Adaptive Inference rather than an artifact of a single high-volatility asset, we conduct parallel validation on the S&P 500 ETF (SPY). We train a Professional Agent on SPY using identical hyperparameters, architectural choices, and learning protocols as the ARKK Professional Agent, with the return observation likelihood instantiated separately for SPY, enabling direct comparison of how regime informativeness affects the frozen–adaptive performance gap.

SPY exhibits substantially lower volatility (annualized 16.8% versus 43.0% for ARKK), weaker regime separation, and more stable return dynamics. This constitutes an out-of-domain stress test for the Adaptive Inference framework: if the Passivity Paradox persists under diminished epistemic opportunity, then it reflects a structural property of belief deployment rather than an artifact of high-volatility environments.

Table A15 summarizes out-of-sample performance across assets and learning modes.

#### 4.3.1. Passivity Paradox Persists Across Volatility Regimes

The Passivity Paradox manifests on both assets, though with different magnitudes. On SPY, the Professional Frozen Agent achieves a Sharpe ratio of 0.91, substantially outperforming its adaptive counterpart (Sharpe 0.67)—a gap of +0.24 Sharpe units attributable solely to belief freezing. On ARKK, the paradox is more pronounced. The Professional Frozen Agent achieves a Sharpe ratio of +0.39 while the Adaptive Agent deteriorates to a Sharpe ratio of −0.28, a gap of +0.67 units.

This pattern provides evidence that the Passivity Paradox generalizes beyond a particular equity instrument; frozen parameters systematically outperform adaptive learning regardless of underlying volatility structure.

#### 4.3.2. Risk-Adjusted Performance and Tail-Risk Reduction on SPY

Notably, the SPY Professional Frozen Agent achieves comparable risk-adjusted returns relative to Buy-and-Hold (Sharpe ratio of 0.91 versus 0.89) while dramatically reducing tail risk. Maximum drawdown contracts from −18.8% (Buy-and-Hold) to −9.0% (Frozen Agent), a 52% reduction in worst-case losses. The position allocation heatmap (Figure A4) reveals the mechanism: the agent maintains a latent long state 69.7% of the time (Table A9), while realized exposure remains positive 92.2% of days due to volatility scaling, confidence gating, and execution friction. The agent tactically reduces positions during high-entropy periods, implementing what we term “uncertainty-aware passive investing”. Because position reductions are triggered by epistemic uncertainty (entropy) rather than predicted negative returns, the strategy functions as an uncertainty-sensitive exposure controller rather than a directional forecaster.

This contrasts with ARKK, where the Frozen Agent underperforms Buy-and-Hold on a Sharpe ratio basis (0.39 versus 0.54) but provides better drawdown protection (−35.8% versus −39.6%). The divergence reflects differences in regime informativeness: SPY’s lower volatility paradoxically makes entropy-triggered risk management more valuable, as the agent avoids the few severe drawdowns without sacrificing much upside.

#### 4.3.3. Adaptive Learning Degrades More Severely Under High Volatility

While the Adaptive Learning Agent underperforms the Frozen Agent’s transfer on both assets, the degradation is asymmetric. On SPY, the Professional Adaptive Agent achieves positive absolute returns (CAGR +12.4%, Sharpe +0.67), merely underperforming the Professional Frozen variant. On ARKK, the Professional Adaptive Agent produces *negative* absolute returns (CAGR −4.4%, Sharpe −0.28) with deeper drawdowns (−41.3%).

This asymmetry reflects differing opportunities for belief corruption. ARKK’s volatile regime structure provides more “noise surface” for adaptive learning to chase spurious patterns, amplifying policy-contingent feedback loops. SPY’s more stable dynamics limit the damage adaptive learning can inflict, allowing positive absolute performance. Nevertheless, frozen belief transfer continues to dominate in terms of risk-adjusted returns and drawdown control, indicating superior robustness even in benign market conditions.

#### 4.3.4. Execution Efficiency Scales with Paradox Magnitude

Consistent with what we term a friction-as-buffer mechanism, execution rates correlate inversely with the magnitude of the Passivity Paradox, that is, with the performance gap between adaptive learning and frozen transfer. By limiting how frequently belief updates are translated into trades, execution frictions (confidence gating, entropy thresholds, and volatility scaling) act as a stabilizing buffer against policy-contingent belief distortions.

On ARKK, the Professional Frozen Agent executes 26.0% of opportunities with turnover of 14.4 units (cumulative absolute changes in portfolio weight), while the Adaptive Agent executes 29.8% with turnover of 17.7 units. On SPY, the Frozen Agent executes only 10.7% with turnover 8.2 units, compared to 16.1% and 12.2 units for the Adaptive Agent.

The SPY Professional Frozen Agent’s lower execution rate reflects higher confidence in maintaining positions, as entropy spikes are rarer in low-volatility environments. This parsimony contributes to its superior performance; fewer trades mean lower transaction costs and reduced exposure to execution timing errors.

#### 4.3.5. Implications for Deployment

These findings establish that the Passivity Paradox is volatility-robust, with the Frozen Agent’s belief transfer outperforming adaptive learning across market regimes. However, the economic value of Adaptive Inference is asset-contingent. On high-volatility assets such as ARKK, the framework provides meaningful drawdown protection but may sacrifice absolute returns; information certainty and risk management come at a cost. On moderate-volatility assets like SPY, the framework can achieve both comparable risk-adjusted returns *and* dramatic tail risk reduction.

Full SPY diagnostics, position allocation heatmaps, and underwater plots are reported in Appendix B.7.

### 4.4. Entropic Sharpe Ratio: Informational Efficiency

Standard risk-adjusted metrics evaluate returns per unit of financial risk, but remain silent on the *informational work* required to generate those returns. On the other hand, Adaptive Inference agents incur explicit cognitive costs through belief updating and uncertainty resolution. To quantify performance along this additional dimension, we introduce the *Entropic Sharpe Ratio* (ESR), a metric measuring excess return per unit of informational change.

**Definition** **1.** *Let $H[Q\left(\pi_{t}\right)]$ denote the Shannon entropy of the posterior belief over policies (action sequences) at time t:*

(28) $$\Delta H_{t}\;=\;H\left[Q\left(\pi_{t-1}\right)\right]-H\left[Q\left(\pi_{t}\right)\right].$$

*In the empirical implementation, policy entropy is measured in bits and captures the agent’s decisional uncertainty rather than its uncertainty over latent market states. The Entropic Sharpe Ratio (ESR) is then defined as*

(29) $$\mathrm{ESR}\;=\;\frac{R_{\mathrm{ann}}-R_{f}}{E\;\left[|\Delta H_{t}|\right]},$$

*where $R_{\mathrm{ann}}$ denotes annualized excess return and $R_{f}$ is the risk-free rate. ESR is expressed in units of percentage excess return per bit of policy-entropy change.*

High ESR indicates that returns are generated with minimal belief revision, corresponding to informationally efficient decision-making. Negative ESR indicates that informational effort destroys value, with belief updates contributing to poorer outcomes.

#### 4.4.1. Training Versus Deployment

During training, the Professional Agent exhibits strongly positive ESR, particularly on decision days when active rebalancing occurs. This reflects the value of epistemic exploration when decisional uncertainty is resolved in a manner aligned with exogenous market dynamics.

However, in out-of-sample deployment the relationship reverses; For the Professional Frozen Agent, ESR remains positive when averaged across all days, reflecting value generated through passive exposure calibrated during training; yet when conditioning on decision days only, that is, when restricting the calculation to periods in which the execution gate is open, ESR becomes sharply negative. Explicit trading decisions destroy informational value even though beliefs remain well calibrated on average.

#### 4.4.2. The Decision-Day Paradox

The empirical results reveal a sign reversal in the marginal productivity of belief revision. During training, larger policy-entropy reductions coincide with positive excess returns, yielding positive ESR on decision days. In out-of-sample deployment, however, the relationship inverts: days with substantial entropy reduction (active rebalancing) are associated with lower subsequent excess returns.

This does not follow mechanically from the definition of ESR; entropy reductions could in principle correlate positively with returns. Therefore, the inversion reflects an empirical change in the marginal economic value of belief updates.

We refer to this sign reversal as the *Decision-Day Paradox*: belief revisions that were economically productive during training become value-destroying when acted upon in non-stationary deployment.

#### 4.4.3. Execution Friction as an Informational Buffer

The Decision-Day Paradox explains why dampened execution ($\eta_{\mathrm{exec}}=0.40$ in testing versus 0.60 in training) improves out-of-sample performance. When decision-day ESR is negative, slower execution acts as a decision-quality filter that limits the economic impact of informationally-destructive belief updates.

When decision-day ESR is negative, reducing execution frequency shifts weight toward value-preserving passive exposure. Thus, execution friction functions as an *uncertainty buffer*, attenuating the impact of informationally destructive decisions without altering underlying beliefs.

#### 4.4.4. Informational Efficiency and the Meaning of ESR

The ESR reframes portfolio performance in thermodynamic terms, with value arising not merely from returns but from returns generated with minimal informational dissipation. Frozen transfer maximizes informational efficiency by preserving calibrated beliefs while limiting destructive intervention. Adaptive learning increases decisional informational throughput but violates structural invariance. This leads to negative informational efficiency despite greater cognitive effort. Importantly, ESR is not an optimization target but a diagnostic quantity, used here to reveal when additional belief updating ceases to be economically productive.

By making the informational cost of decision-making explicit, the ESR provides a principled diagnostic linking belief dynamics, execution behavior, and economic performance, thereby completing the empirical characterization of the Passivity Paradox.

Formally, let $\eta_{\mathrm{exec}}\in \left[0,1\right]$ denote the fraction of decision signals that are executed. Effective informational efficiency can be expressed as a convex combination:

(30) $${\mathrm{ESR}}_{\mathrm{eff}}=\left(1-\eta_{\mathrm{exec}}\right)\;{\mathrm{ESR}}_{\mathrm{passive}}+\eta_{\mathrm{exec}}\;{\mathrm{ESR}}_{\mathrm{decision}}$$

where ${\mathrm{ESR}}_{\mathrm{passive}}$ measures the excess return per unit entropy change accrued on non-decision days (beliefs held fixed) and ${\mathrm{ESR}}_{\mathrm{decision}}$ measures informational efficiency on active rebalancing days.

In the Professional Frozen Agent, ${\mathrm{ESR}}_{\mathrm{passive}}=+23.2\%$/bit, while ${\mathrm{ESR}}_{\mathrm{decision}}=-20.8\%$/bit. This implies that:

- At $\eta_{\mathrm{exec}}=0.60$: ${\mathrm{ESR}}_{\mathrm{eff}}=-3.2\%$/bit.
- At $\eta_{\mathrm{exec}}=0.40$: ${\mathrm{ESR}}_{\mathrm{eff}}=+5.6\%$/bit.

Reducing execution frequency shifts weight away from value-destroying decision-day updates towards value-generating passive exposure, formalizing execution friction as an optimal uncertainty buffer that limits action when belief updates are informationally destructive.

#### 4.4.5. Information-Efficiency Diagnostic

To complement the standard risk-free–aware risk-adjusted metrics, Appendix F reports an information-efficiency diagnostic based on the market-state posterior entropy and the ESR, computed on the same daily excess return series used in this section. These results help to interpret *why* Frozen vs. Adaptive Agents differ out-of-sample by measuring excess return per unit of belief updating (bits).

#### 4.4.6. From Mechanism to Statistical Validation

The ESR analysis provides a mechanistic explanation for why Frozen and Adaptive Agents diverge out-of-sample by making the informational cost of decision-making explicit. In the next subsection, we subject these findings to formal statistical validation. Using HAC-robust tests and entropy-conditioned regime splits, we assess whether the observed performance differentials are statistically significant and robust to temporal dependence and regime heterogeneity in terms of both returns and informational efficiency.

### 4.5. Statistical Validation

We validate the empirical findings using complementary statistical procedures designed to assess economic dominance, variance efficiency, and distribution-free robustness. All tests are conducted on daily risk-free-adjusted excess returns over the out-of-sample period (25 August 2022–5 December 2025; n=824 trading days) for ARKK in the Professional Agent implementation. Standard errors account for serial correlation and heteroskedasticity.

#### 4.5.1. Test Suite

We employ three classes of tests: (i) HAC-robust *t*-tests on mean excess returns to assess economic dominance; (ii) Diebold–Mariano (DM) tests on squared excess returns to compare variance efficiency; (iii) Block bootstrap inference to provide nonparametric confirmation under minimal distributional assumptions [77,78].

#### 4.5.2. Unconditional Performance Tests

Table 5 reports pairwise comparisons for the Professional Agent across learning modes.

##### Mean Return Dominance

Frozen transfer for the Professional Agent significantly outperforms adaptive learning in terms of mean excess return:

$$\Delta \mu_{\mathrm{Frozen}-\mathrm{Adaptive}}\;=\;+17.5\%\;\left(\mathrm{annualized}\right),\;t_{\mathrm{HAC}}=2.59,\;p=0.010.$$

Block bootstrap inference confirms robustness (p=0.008), establishing economically and statistically significant return dominance of frozen transfer.

#### 4.5.3. Variance–Return Tradeoff

The Diebold–Mariano statistic comparing squared excess returns is positive (DM =+3.99, p<0.001), indicating that the Professional Frozen Agent exhibits larger squared excess-return realizations on average relative to the Adaptive Agent. This result is not contradictory; HAC tests evaluate mean performance, while DM tests evaluate variance efficiency. Frozen transfer accepts higher variance in exchange for superior mean returns, yielding higher Sharpe ratios.

#### 4.5.4. Regime-Conditional Validation

The Passivity Paradox predicts that frozen transfer should dominate adaptive learning when regime signals are informative, but not when regime uncertainty is maximal. To test this mechanism directly, we condition excess-return differences on inferred regime uncertainty, measured by posterior entropy of the market-state belief.

We partition the sample into entropy terciles (low, mid, high), corresponding to confident, intermediate, and ambiguous regime inference. If the paradox reflects overupdating in environments where signals are policy-contaminated, then mean-return dominance of the Frozen Agent should concentrate in low- and mid-entropy regimes and attenuate under high entropy.

Table 6 reports regime-conditional HAC *t*-tests for Frozen versus Adaptive learning. Each subsample contains approximately one-third of the observations, ensuring comparable statistical power across entropy regimes.

#### 4.5.5. Statistical Summary

Three conclusions emerge:

1. Frozen transfer significantly outperforms Adaptive learning in mean excess returns.
2. This dominance is concentrated in the low- and mid-entropy regimes, where regime inference is informative.
3. Adaptive learning reduces variance under maximal uncertainty but does not generate superior mean performance.

These results support the interpretation that the Passivity Paradox reflects a regime-contingent interaction between belief updating and endogenous feedback, rather than stemming from sampling noise.

## 5. Discussion

### 5.1. From Active Inference to Economic Decision Theory

Active Inference originated as a unifying framework for perception, learning, and action in biological systems [22]. This paper demonstrates that its core principles extend naturally to financial decision-making, where agents must infer latent market regimes from noisy observations, select actions under uncertainty, and adapt to structural nonstationarity.

Translating Active Inference into finance as Adaptive Inference requires explicit treatment of endogeneity and controllability, features that are largely absent in neuroscience but fundamental in markets. Portfolio actions influence future observations through P&L and realized risk, giving rise to policy-dependent feedback loops formalized by the Lucas Critique [14]. Our framework resolves this tension through a controllability-based decomposition that separates uncontrollable environmental signals from controllable outcomes, thereby ensuring that belief updating preserves structural invariance.

The benchmark results sharpen this distinction along two axes. First, classical valuation models—BSM Real Options and Stochastic DCF—operate under implicit assumptions that uncertainty is either resolved ex ante or irrelevant for control. When evaluated under identical execution and risk-free conventions, both underperform out-of-sample on ARKK, exhibiting negative Sharpe ratios and substantial drawdowns (Section 4.1.1; Appendix G.1 and Appendix G.2). These results reflect structural limitations: valuation rules that do not incorporate uncertainty-sensitive control are fragile in regime-rich nonstationary environments.

Second, the Hedge benchmark [23] isolates adaptivity itself. As a provably adaptive no-regret online learning algorithm, Hedge achieves competitive risk-adjusted returns (Sharpe ratio of +0.40) without maintaining a generative model of latent market states. This demonstrates that performance adaptivity does not require inference. However, Hedge reallocates weight purely on realized performance and does not represent beliefs, posterior uncertainty, or epistemic value. Therefore, the contrast is structural: Adaptive Inference embeds learning within a generative model of regimes, enabling belief-state decomposition and explicit information-seeking motives which model-free adaptivity cannot provide.

Thus, finance does not merely adopt Active Inference; rather, it *disciplines* it. The market setting reveals necessary causal constraints on learning and control that are less visible in biological systems. Successful inference-guided decision-making requires not only Bayesian updating but principled restrictions on *what* is learned and *when* action should be taken.

### 5.2. Toward Multi-Agent Market Ecologies

We acknowledge that the framework developed in this paper is single-agent: one Adaptive Inference agent interacts with market dynamics treated as exogenous, abstracting from endogenous price impact and strategic interaction. However, Active Inference provides natural machinery for multi-agent extension through hierarchical generative models in which one agent’s policy becomes part of another’s environment [90]. In financial markets, this nesting formalizes *reflexivity* [91]: each agent’s Markov blanket separates beliefs from external states, but those external states include the actions of other agents, creating recursive inference about inference.

The heterogeneous cognitive profiles developed here—Bearish, Neutral, and Professional—provide a natural basis for agent populations, echoing Arthur et al. [92] and subsequent heterogeneous-agent models [93,94]. However, interacting Adaptive Inference agents would differ fundamentally from classical agent-based models: agents would not follow fixed heuristic rules but continuously update generative models of latent regimes through expected free energy (EFE) minimization, implying co-evolution of beliefs—not merely strategies—with market conditions.

Two theoretical implications follow. First, endogeneity compounds; each agent’s actions alter the signals others use for inference, potentially amplifying volatility through recursive belief–action feedback loops and generating population-level variants of the Passivity Paradox. Second, because each agent maintains an explicit and auditable generative model, a regulator could in principle inspect the cross-sectional distribution of regime beliefs and detect belief clustering—a measurable precursor to crowded trades and systemic fragility—before correlated positions fully materialize.

Formal multi-agent simulation and equilibrium characterization remain important directions for future research.

Beyond explicit market simulation, recent advances in multi-agent scientific discovery suggest a complementary research direction. Agentic graph reasoning frameworks in which autonomous agents iteratively expand and refine knowledge graphs through reasoning-driven feedback loops have demonstrated the capacity to uncover non-obvious cross-domain relationships in complex systems [95,96]. Representing the financial market as an evolving knowledge graph in which nodes encode risk factors, asset classes, macro regimes, and regulatory states while edges encode inferred causal and correlational dependencies, could enable swarms of Adaptive Inference agents to autonomously discover latent risk factors and structural vulnerabilities that no single-agent architecture would identify. This knowledge graph extension would move from equilibrium modeling toward distributed risk discovery within complex financial ecologies.

### 5.3. Benchmark Failure as a Limiting-Case Diagnostic

The empirical underperformance of BSM Real Options and Stochastic DCF provides critical insight into the limits of classical finance when deployed as trading rules rather than valuation heuristics.

The BSM Real Options strategy corresponds to an edge case of Adaptive Inference in which regime uncertainty collapses and volatility is treated as known. In this regime, decision-making reduces to static option-implied exposure. Out-of-sample, this approach fails to adapt to regime shifts in volatility and trend persistence, producing inferior risk-adjusted performance and drawdowns exceeding those of Buy-and-Hold.

The Stochastic DCF benchmark highlights a different failure mode. While it incorporates Bayesian updating of growth expectations, its valuation mechanism amplifies persistent growth estimates over long horizons, producing extreme raw fair values on a substantial fraction of days. While signal-side clamps—hard bounds applied to inferred valuation signals before they are mapped into portfolio actions—can prevent these instabilities from translating into catastrophic trades, the resulting strategy still performs poorly out-of-sample. This behavior reflects a deeper structural issue: when current cash flows are negligible, Stochastic DCF valuation becomes dominated by terminal assumptions, rendering it ill-suited for growth assets with unstable regimes.

These failures strengthen rather than weaken the theoretical contribution of this paper. They empirically validate the proposition that neoclassical finance models are not wrong, but are *representationally limited*. They emerge as limiting cases of Adaptive Inference precisely when epistemic terms vanish. When uncertainty matters, and especially when actions influence future observations, valuation without explicit inference and control is insufficient.

### 5.4. The Passivity Paradox as an Endogeneity Result

The central empirical finding of the *Passivity Paradox* is best understood as a consequence of endogenous learning under policy-dependent observations. When agents update beliefs using signals that depend on their own actions, adaptive learning contaminates inference with self-induced noise. In our setting, this manifests as belief drift that degrades regime identification and risk-adjusted performance.

Importantly, the paradox does not imply that learning is inherently harmful; rather, it identifies a sharp boundary condition: learning is beneficial only when restricted to observation channels that remain invariant under policy changes. Multi-modality surgical learning satisfies this condition by aggregating exogenous signals—returns, volatility, momentum, and volume—that preserve informational meaning regardless of the agent’s actions. The Neutral Agent’s reversal of the paradox provides empirical confirmation of this mechanism.

This result generalizes classical insights from information economics. Grossman and Stiglitz [55] showed that information is valuable only when it conveys structure not already reflected in prices. Our results extend this logic to Adaptive Agents: epistemic effort improves performance only when it targets uncontaminated information sources. Otherwise, additional learning accelerates divergence rather than convergence.

**Figure A1:**
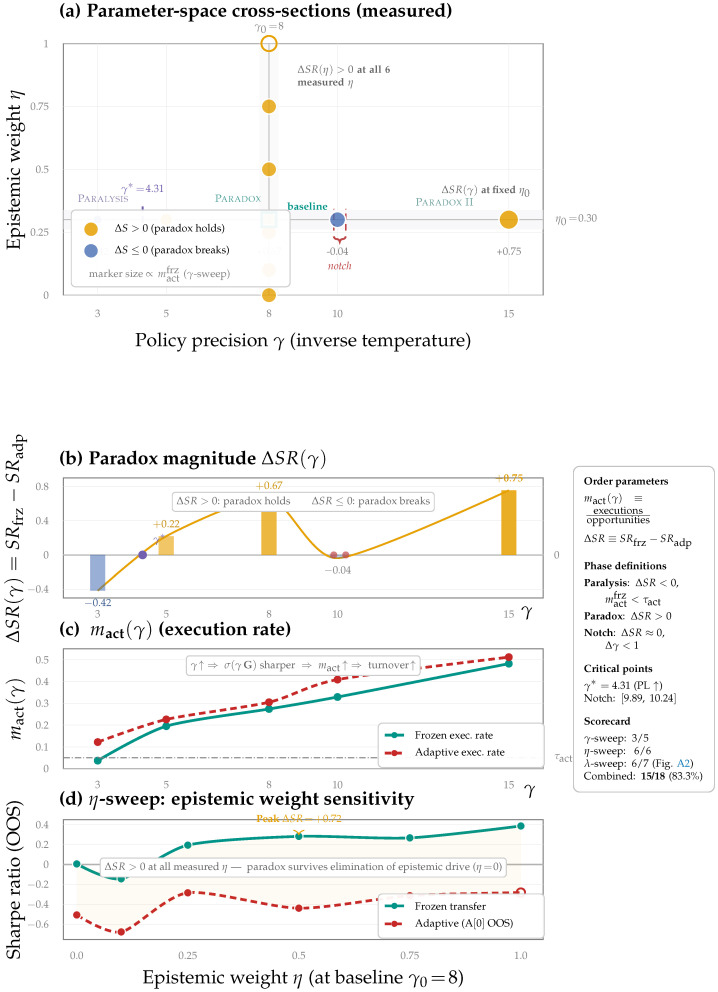
Phase diagram of the Passivity Paradox: policy precision (γ) and epistemic weight (η) cross-sections. (**a**) Cross-section 1 (horizontal): ΔSR(γ) at fixed $\eta_{0}\;=\;0.30$. Marker color encodes ΔSR sign (amber: paradox holds; blue: paradox breaks); marker size scales with the execution-rate order parameter $m_{\mathrm{act}}^{\mathrm{frz}}$. Phase boundary $\gamma^{*}\;=\;4.31$ and notch [9.89,10.24] computed via piecewise-linear zero-crossings. Cross-section 2 (vertical): ΔSR(η)>0 at all six tested η values (η∈{0,0.10,0.25,0.50,0.75,1.00}). (**b**) Non-monotonic ΔS(γ): peak paradox ΔSR = +0.75 at γ = 15. (**c**) Monotonic execution rate $m_{\mathrm{act}}\left(\gamma \right)$; $\tau_{\mathrm{act}}\;=\;0.05$ marks the decisional viability floor. (**d**) η-sweep: The Frozen Agent outperforms the Adaptive Agent at every epistemic weight, including η = 0. Loss aversion (λ) sweep reported separately in Figure A2. ARKK ETF, OOS 25 August 2022 to 5 December 2025, N = 824 days, 164 decision opportunities.

**Figure A4:**
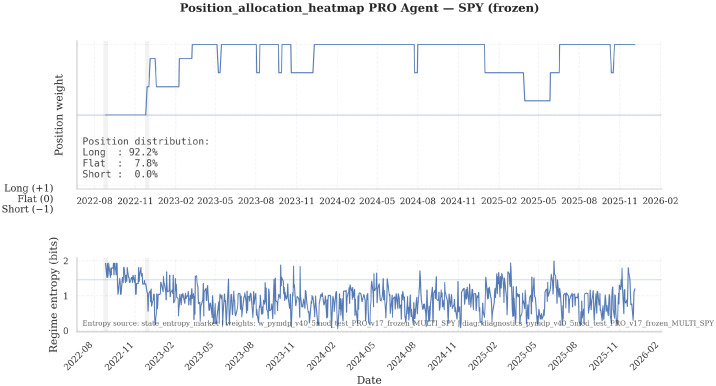
Position allocation and regime entropy over time for the Professional Agent on SPY (Frozen Agent). The upper panel shows executed position weights: the agent maintains positive exposure 92.2% of the time, with tactical flat periods (7.8%) and no short positions (0.0%). Gray shaded regions indicate periods where the agent reduces exposure to zero. The lower panel displays regime entropy in bits; entropy spikes above the confidence threshold (horizontal line at ≈1.5 bits) correlate with position reductions, implementing “uncertainty-aware passive investing”. Notable flat periods occur during October–November 2022 (coinciding with Fed tightening uncertainty) and March–April 2025 (market correction), where elevated entropy triggers precautionary de-risking.

The Passivity Paradox also connects to classical results in statistical learning theory. The Frozen Agent’s advantage over the Adaptive Agent is structurally analogous to the bias–variance tradeoff in supervised learning: frozen beliefs carry higher bias (they cannot track distributional drift) but exhibit zero variance from estimation noise, while adaptive beliefs reduce bias at the cost of injecting policy-contingent variance into posterior updates. In nonstationary environments with short evaluation horizons, this variance cost dominates the bias reduction, a finding that is consistent with the well-documented tendency of complex models to overfit in-sample while degrading out-of-sample [97,98]. The behavioral parameter sweeps in Appendix B provide empirical boundary conditions: policy precision (γ) exhibits a non-monotonic relationship with paradox magnitude (Figure A1b), revealing a phase transition from decision paralysis (low γ, where adaptive learning rescues an otherwise inert agent) through the paradox regime (moderate-to-high γ, where frozen beliefs dominate) to extreme over-commitment (very high γ, where both agents degrade but the Adaptive Agent degrades faster). This non-monotonic structure mirrors the classical U-shaped generalization curve, with γ playing the role of model complexity. The cross-asset attenuation on SPY further supports this interpretation: in lower-volatility environments, the “noise surface” available for overfitting shrinks, reducing but not eliminating the variance penalty of adaptation.

### 5.5. Risk Shaping Versus Return Maximization

In high-volatility regime-rich environments, Adaptive Inference consistently reshapes risk rather than maximizing raw returns. On ARKK, Frozen Agents deliver economically meaningful reductions in volatility and drawdowns relative to both Buy-and-Hold and classical valuation strategies even when they underperform on terminal wealth. The Professional Frozen Agent achieves a maximum drawdown of −35.8% compared to −39.6% for Buy-and-Hold, a reduction of nearly 4 percentage points, while maintaining positive risk-adjusted returns.

Cross-asset validation on SPY reveals that risk shaping extends to low-volatility environments, though with a qualitatively different value proposition. On SPY, the Professional Frozen Agent achieves comparable risk-adjusted returns (Sharpe ratio of 0.91 versus 0.89 for Buy-and-Hold) while dramatically reducing tail risk; maximum drawdown contracts from −18.8% to −9.0%, a 52% reduction in worst-case losses. Annualized volatility falls from 16.8% to 10.8%, reflecting the agent’s uncertainty-aware position management. Unlike ARKK, where inference sacrifices absolute returns for drawdown protection, SPY demonstrates that inference-driven risk shaping can deliver substantial tail-risk reduction while preserving comparable risk-adjusted performance in stable market environments.

### 5.6. Interpretability, Auditability, and Regulatory Alignment

A defining advantage of Adaptive Inference is that interpretability is guaranteed by construction. Beliefs, preferences, and objectives are explicit components of the generative model, allowing every action to be traced to posterior regime probabilities (state beliefs) along with a transparent decomposition of expected free energy (EFE) into epistemic and pragmatic terms [17,30].

This contrasts with deep reinforcement learning, classical valuation-based strategies, and model-free online learning methods such as Hedge, none of which provide an internal causal model from which decisions can be derived and audited. In regulated financial environments, post hoc explanations are insufficient. Without explicit belief states and causal structure, adaptive systems cannot distinguish learning from self-induced noise. The benchmark failures underscore this point; neither BSM Real Options nor Stochastic DCF provides an internal representation of uncertainty that is suitable for audit or control.

The Entropic Sharpe Ratio further strengthens this alignment by quantifying information efficiency in the form of excess return per unit of belief update. Strategies that generate returns with minimal informational dissipation exhibit lower turnover, greater stability, and improved auditability. Appendix F Equation (A1) provides the complete explanation and detail of this novel metric.

### 5.7. Limitations and Scope

Several limitations merit discussion. First, our empirical analysis focuses on two-asset (ARKK and SPY) time series. Extending Adaptive Inference to multi-asset portfolios would require hierarchical generative models capable of representing cross-sectional dependencies. Second, discretized regime representations approximate continuous dynamics; richer state spaces may improve inference at the cost of computational complexity. Third, we abstract from market impact and liquidity constraints, which would introduce additional endogeneity and likely amplify the Passivity Paradox.

Fourth, the surgical learning protocol rests on a pre-specified classification of observation modalities as exogenous (learnable) or endogenous (frozen). In the present implementation, returns, momentum, volatility regimes, and volume shocks are treated as exogenous, while portfolio P&L is treated as endogenous. This classification is defensible for the single-agent, single-asset setting studied here, where the agent’s trades are too small to move prices; however, in higher-capacity or multi-agent settings, variables such as trading volume and realized volatility may acquire endogenous components through market impact and strategic interaction, blurring the exogenous–endogenous boundary. Future work should explore dynamic signal attribution methods such as time-varying Granger causality tests or information-theoretic measures of causal influence [99] in order to adaptively reclassify observation channels as their endogeneity properties shift. The framework’s robustness to moderate misclassification is partially supported by the Passivity Paradox itself; if all channels were substantially contaminated, freezing *all* learning (not just endogenous channels) would be expected to dominate, which is precisely the Frozen Agent’s operating mode. Thus, the persistence of the paradox provides indirect evidence that the exogenous channels retain sufficient signal quality to justify selective updating. Finally, multi-agent settings in which agents form beliefs about others’ beliefs remain an open direction. Active Inference provides natural machinery for this extension through hierarchical generative models in which one agent’s policy becomes part of another’s environment [17]. In financial markets, such “inference about inference” would formalize phenomena such as crowded trades, reflexivity [91], and strategic liquidity withdrawal, where the relevant nonstationarity is itself endogenous to collective belief dynamics. Extending surgical learning to distinguish not only agent generated from market generated signals but also signals generated by *other* adaptive agents is a necessary step towards deploying Adaptive Inference in realistic market ecologies.

## 6. Conclusions

This paper develops *Adaptive Inference* as a unified framework for multi-period portfolio management that integrates inference, control, and execution under uncertainty. Adaptive Inference provides a theory of the Bayesian mechanics of economic choice in which investment decisions arise from the coupled dynamics of belief updating, preference encoding, and action selection under endogenous uncertainty rather than from fixed objectives or converged expectations.

Theoretically, we show that BSM Real Options, Stochastic DCF, and portfolio mean–variance optimization arise as edge cases of expected free energy (EFE) minimization when epistemic components vanish. Therefore, classical finance models are not incorrect but rather structurally restricted: they assume that inference has already converged and that belief updating plays no active role in control. The controllability decomposition clarifies why valuation rules fail when actions influence future observations.

Methodologically, we introduce surgical learning, confidence gating, and the Entropic Sharpe Ratio (based on policy-posterior entropy) to address endogeneity, execution under uncertainty, and informational efficiency. These tools provide a principled response to the Lucas Critique in adaptive investing systems.

Empirically, we document the Passivity Paradox and its boundary conditions. Cross-asset validation confirms that frozen inference dominates adaptive learning across both tested market environments: in ARKK, the Professional Frozen Agent outperforms adaptive by +0.67 Sharpe ratio units, while in SPY the gap is +0.24 Sharpe ratio units. Notably, the Professional Frozen Agent achieves comparable risk-adjusted returns on the SPY ETF (Sharpe ratio of 0.91 versus 0.89 for Buy-and-Hold) while reducing maximum drawdown by 52%, demonstrating that inference-driven risk shaping provides value across volatility regimes. The classical finance benchmarks (BSM Real Options and Stochastic DCF) show poor out-of-sample performance under identical execution conventions, reinforcing the necessity of explicit uncertainty-sensitive control.

Taken together, these results support a reframing of sequential portfolio management as *inference-guided risk shaping* rather than return maximization. Adaptive Inference offers a transparent and theoretically-grounded alternative to black-box reinforcement learning, static valuation rules, and model-free online learning, revealing when learning helps, when it harms, and why restraint can be optimal.

## 7. Appendix Roadmap

The appendices provide a complete and auditable extension of the main text. They are organized to progress from the canonical specification of the Professional Adaptive Inference Agent, through robustness analysis and comprehensive empirical reporting, to algorithmic auditability, information-theoretic diagnostics, formal statistical validation, comparative benchmark models, interpretability, and theoretical proofs.

This ordering ensures clarity, reproducibility, and theoretical coherence while keeping the main body focused on the core empirical findings and conceptual contributions.

- Appendix A (Parameterization of the Professional Agent). Canonical specification of the Professional Adaptive Inference Agent used throughout the paper. This appendix documents the full state space, observation modalities, preference structure, precision parameters, confidence gating, execution constraints, and learning configuration. All subsequent results are derived from this fixed parameterization.
- Appendix B (Robustness and Sensitivity Analysis). Robustness checks for the Professional Agent, including execution speed, transaction cost sensitivity, volatility targeting variants, and auxiliary transfer experiments on SPY. These results demonstrate that the main conclusions are not driven by fragile execution or cost assumptions.
- Appendix C (Complete Results by Cognitive Profile). Full in-sample and out-of-sample performance tables, equity curves, and diagnostic figures for all cognitive profiles (Bearish, Neutral, and Professional) reported for both Frozen and Adaptive variants.
- Appendix D (Algorithmic Details and Auditability). A complete algorithmic specification of the Adaptive Inference portfolio agent, including fixed-placement pseudocode, belief updates, policy evaluation, confidence gating, surgical learning rules, execution logic, logging conventions, and reproducibility safeguards. This appendix enables full reconstruction and independent verification of every reported decision.
- Appendix E (Complete Cross-Profile Summary). A consolidated reference appendix summarizing out-of-sample performance across all cognitive profiles, learning modes, and benchmark models is provided for completeness and direct comparison.
- Appendix F (Entropy Diagnostics and Statistical Validation). Information-theoretic diagnostics reporting market-state entropy, belief uncertainty, regime-conditioned return differences, and the Entropic Sharpe Ratio (ESR) for all agents and benchmarks. Formal statistical validation of the main results, including HAC-robust mean-difference tests, Diebold–Mariano variance-efficiency tests, stationary and block bootstrap inference, and regime-conditional performance evaluation based on belief entropy.
- Appendix G (Benchmark Models). Complete methodological specification of the benchmark models used for comparison: the BSM Real Options framework, the Stochastic DCF model with Bayesian Kalman filtering and Monte Carlo valuation, and the Hedge multiplicative-weights expert mixture with post hoc validity audit. All benchmarks are aligned to the Professional Agent’s execution, friction, and risk-free conventions.
- Appendix H (Hedge (Multiplicative Weights Update) Benchmark). Full specification and out-of-sample validation of the Hedge expert-mixture benchmark, implementing multiplicative-weights updates over eight classical trading strategies under friction- and risk-layer conditions identical to the Professional Agent. This appendix documents the expert panel construction, minimax learning-rate derivation, a three-part invariance audit confirming the absence of look-ahead bias, diagnostics including equity curves and simplex evolution, and sensitivity analysis across learning rates and transaction costs. The benchmark serves as a credible adversarial reference point that isolates the structural advantages of the Adaptive Inference framework.

## Figures and Tables

**Table 1:** Complete specification of three agent cognitive profiles.

Parameter	Bearish	Neutral	Professional
*Planning Architecture*			
Policy horizon *T*	2	1	3
Policy space |Π|	16	4	64
$H_{\max}$ (nats)	2.77	1.39	4.16
*Behavioral Finance*			
Loss aversion $\lambda_{\mathrm{eff}}$	1.75	1.50	1.00
Scaling κ	2.5	3.0	2.0
$C^{\mathrm{pnl}}-vector$ ^a^	[2.5,0.75,−2.63,−4.38]	[3.0,0.9,−2.7,−4.5]	[4.0,1.2,−1.2,−4.0]
*Inference Parameters*			
Policy precision γ	5.5	10.0	8.0
Action precision α	3.0	3.0	4.0
Epistemic weight $\eta_{\mathrm{epi}}$	1.0	1.0	1.0
Precision weighting ^b^	No	Yes	No
*Learning Protocol*			
$\eta^{\mathrm{ret}}$	0.08	0.08	0.04
$\eta^{\mathrm{pnl}}$	0.00	0.00	0.00
$\eta^{\mathrm{aux}}$	0.00	0.05	0.00
Forgetting ω	0.992	0.992	0.99
Learning scope	$A^{\mathrm{ret}}$ only	$A^{\mathrm{ret},\mathrm{mom},\mathrm{vol},\mathrm{vlm}}$	$A^{\mathrm{ret}}$ only
*Confidence Gating*			
$p_{\min}$	0.20	0.22	0.22
$H_{\mathrm{gate}}/H_{\max}$	0.85	0.90	0.96
*Risk Management*			
Target volatility	20%	20%	40%
Max step size (train)	0.40	0.40	0.60
Max step size (test)	0.40	0.40	0.40
Position bounds	[−1,+1]	[−0.5,+0.5]	[−1,+1]

^a^ The P&L utility vector $C^{\mathrm{pnl}}$ for the Professional Agent is constructed by applying a symmetric scaling factor κ=2.0 to discretized P&L outcome bins with unit loss aversion (λ=1), yielding a moderate curvature that penalizes large losses without introducing asymmetric behavioral bias. ^b^ Precision weighting refers to entropy-normalized policy precision rather than hierarchical precision learning.

**Table 2:** Out-of-sample performance, showing Adaptive Inference agents vs. the Buy-and-Hold benchmark.

		Performance				Behavior		
Profile	Mode	Sharpe	Sortino	MaxDD	CAGR	Long ^†^	Exec	ΔSR
Bearish (λ = 1.75)	Frozen	−0.71	−0.90	−33.9	−7.7	54.2	29.1	+0.43
	Adaptive	−1.13	−1.41	−45.8	−12.8	50.4	35.4	
Neutral (λ = 1.50)	Frozen	−1.44	−1.76	−40.6	−13.2	21.2	67.3	−1.69
	Adaptive	+0.25	+0.31	−8.8	+7.4	40.0	49.7	
Professional (λ = 1.00)	Frozen	+0.39	+0.53	−35.8	+12.3	65.0	26.0	+0.67
	Adaptive	−0.28	−0.35	−41.3	−4.4	65.6	29.8	
Buy-and-Hold	—	+0.54	+0.80	−39.6	+20.5	100	—	—

*Notes*: Test period: 25 August 2022–5 December 2025 (824 trading days). λ = loss aversion parameter; λ=1.0 indicates symmetric preferences, λ>1.0 indicates asymmetric loss aversion [9]. MaxDD and CAGR in %. Long/Exec in %. ΔSharpe Ratio (SR) = Frozen − Adaptive; positive indicates Passivity Paradox (Frozen dominates), negative indicates reversal (Adaptive dominates). All returns net of 5 bps transaction costs [67,68]. Bold = best-in-class. ^†^ Long % reflects the latent position state distribution $s_{t}^{\left(p\right)}=\mathrm{Long}$; realized portfolio weights exhibit higher long exposure due to execution friction (see Figure 9).

**Table 3:** Out-of-sample performance (test period) under identical execution and risk-free conventions.

Model	Sharpe	Sortino	MaxDD	CAGR
Buy & Hold (ARKK)	+0.54	+0.80	−39.6%	+20.5%
BSM Real Options	−0.16	−0.20	−53.7%	−10.0%
Stochastic DCF	−0.52	−0.62	−70.7%	−21.5%
**Hedge Algorithm**	+0.40	+0.55	−26.0%	+11.2%
**Professional Frozen**	+0.39	+0.53	−35.8%	+12.3%

*Notes*: Test period: 25 August 2022–05 December 2025. Sharpe/Sortino ratios are computed on daily risk-free-adjusted excess returns aligned to the ARKK trading calendar. Transaction costs are 5 bps per executed change in exposure. All benchmark execution and risk controls are matched to the Professional Agent’s conventions to ensure strict comparability. The Hedge benchmark uses minimax-optimal $\eta^{*}$ with no hyperparameters tuned to test data. Bold rows denote adaptive methods.

**Table 4:** Passivity paradox mechanism: belief drift and performance degradation.

Profile	$∥\Delta A^{\left(r\right)}{∥}_{F}$	ΔSharpe Ratio	ΔMaxDD (pp)	Effect
Bearish	0.888	−0.42	−11.9	Standard
Neutral	0.240	+1.69	+31.8	Inverted
Professional	1.139	−0.66	−5.5	Standard

**Table 5:** Unconditional statistical tests for the Professional Agent on ARKK (Out-of-Sample).

Comparison	$\Delta \mu_{\mathrm{ann}}$	$t_{\mathrm{HAC}}$	$p_{\mathrm{HAC}}$	DM	$p_{\mathrm{DM}}$	$p_{\mathrm{boot}}$
Frozen vs. Adaptive	+17.5%	+2.59	0.0097 ***	+3.99	<0.001 ***	0.008
Frozen vs. Buy-&-Hold	−11.4%	−0.85	0.393	−5.06	<0.001 ***	0.398
Adaptive vs. Buy-&-Hold	−28.8%	−1.87	0.062 ^†^	−6.02	<0.001 ***	0.057 ^†^

*Notes*: Daily risk-free-adjusted excess returns, out-of-sample period (26 August 2022–05 December 2025; T=823). $\Delta \mu_{\mathrm{ann}}$ denotes the annualized difference in mean excess returns between strategies. $t_{\mathrm{HAC}}$ is the heteroskedasticity- and autocorrelation-consistent *t*-statistic testing equality of mean returns (Newey–West, 20 lags). $p_{\mathrm{HAC}}$ reports the corresponding two-sided significance level. Diebold–Mariano (DM) statistics test differences in predictive accuracy using squared excess returns as the loss function. $p_{\mathrm{boot}}$ denotes block bootstrap significance under temporal dependence (block length = 20, B=10,000). *** p<0.01, ^†^ p<0.10.

**Table 6:** Regime-conditional mean excess return tests: Frozen vs. Adaptive (Professional Agent, ARKK).

Entropy Regime	*T*	$\Delta \mu_{\mathrm{ann}}$	$t_{\mathrm{HAC}}$	$p_{\mathrm{HAC}}$
Unconditional	823	+17.5%	+2.59	0.0097 ***
Low Entropy	274	+43.4%	+2.17	0.030 *
Mid Entropy	274	+22.6%	+2.56	0.011 *
High Entropy	275	−13.5%	−1.50	0.132

*Notes*: $\Delta \mu_{\mathrm{ann}}$ denotes the annualized mean excess return difference (Frozen minus Adaptive). Entropy regimes are defined by terciles of the market-state posterior entropy. HAC standard errors use 20 lags; minimum observations per regime = 30. *** p<0.01, * p<0.05.

**Figure A2:**
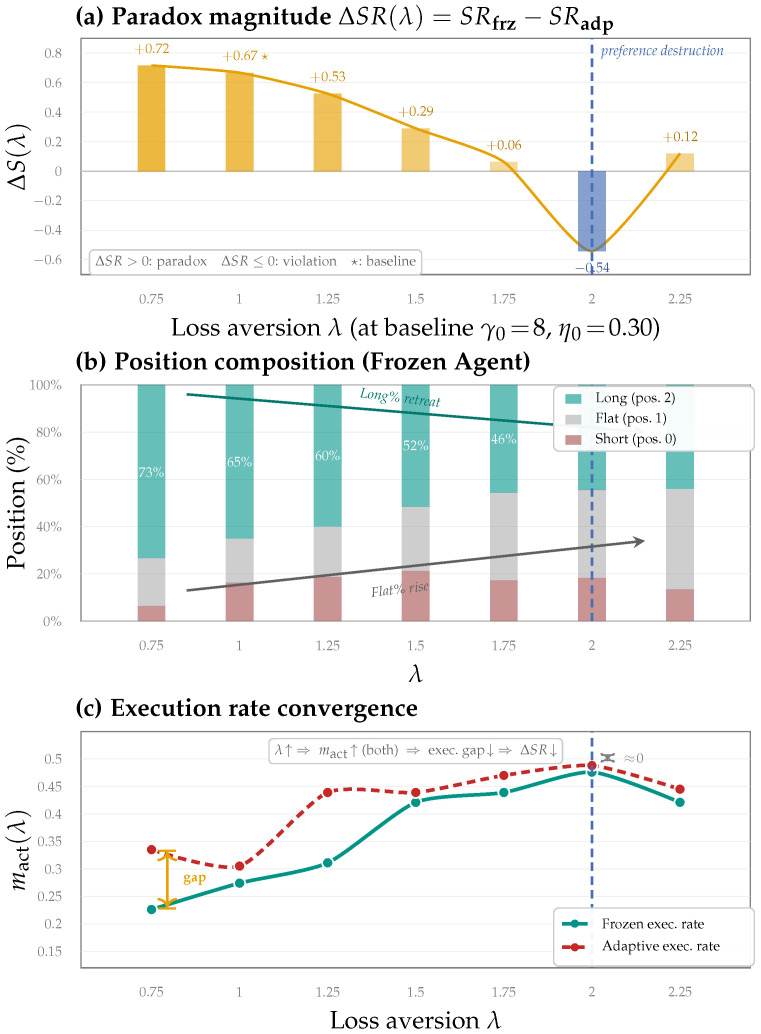
Loss aversion (λ) sweep: behavioral decomposition of the Passivity Paradox. All sweeps at fixed $\gamma_{0}\;=\;8$, $\eta_{0}\;=\;0.30$ (baseline cognitive architecture). (**a**) Paradox magnitude $\Delta SR\left(\lambda \right)=SR_{\mathrm{frz}}-SR_{\mathrm{adp}}$. The paradox holds at six of seven configurations (85.7%). The single reversal at λ = 2.00 (ΔSR=−0.54) marks a preference-destruction boundary where loss penalties exceed twice the gain rewards ($C^{\left(\mathrm{PnL}\right)}=\left[+4.0,+1.2,-2.4,-8.0\right]$). Star (^★^) marks baseline λ = 1.00. (**b**) Frozen Agent position allocation. Long exposure declines monotonically from 73% (λ = 0.75) to 44% (λ = 2.25) as loss aversion erodes equity risk premium capture; flat allocation rises correspondingly. (**c**) Execution rates for both learning modes. At low λ, frozen trades at roughly two-thirds the adaptive rate (22.6% vs. 33.5%); at high λ, both converge near 47–49%. The paradox is strongest where the execution gap is widest. ARKK ETF, OOS 2022-08 to 2025-12, N = 824 days, 164 decision opportunities.

**Figure A3:**
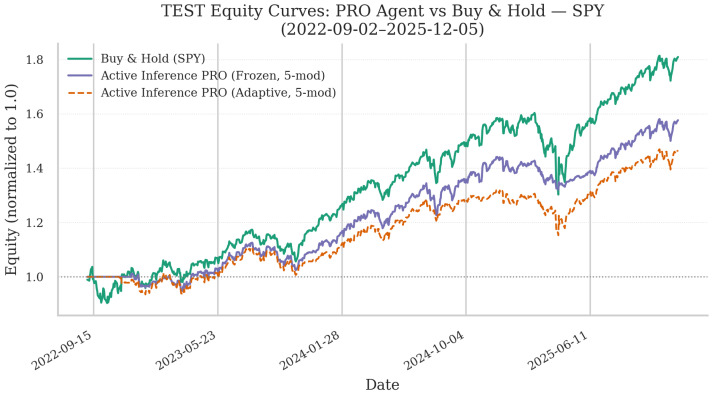
Out-of-sample equity curves for the Professional Agent on SPY (Frozen and Adaptive Agents) compared to Buy-and-Hold. Results are normalized to 1.0 at the start of the test period (2 September 2022, to 5 December 2025). The Frozen Agent (purple) achieves terminal wealth of approximately 1.58× initial capital with a Sharpe ratio of 0.91, achieving comparable risk-adjusted performance to Buy-and-Hold (green, 1.85×, Sharpe ratio of 0.89) on a risk-adjusted basis despite lower absolute returns. The Adaptive Agent (orange) reaches 1.45× with a Sharpe ratio of 0.67. Sharpe and Sortino ratios are computed on daily excess returns relative to the risk-free rate.

**Figure A5:**
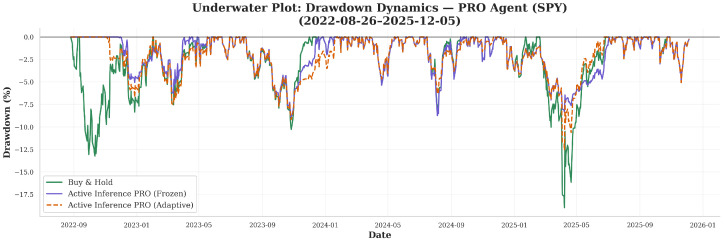
Underwater (drawdown) plot for the Professional Agent on SPY during out-of-sample evaluation (26 August 2022 to 5 December 2025), compared to Buy-and-Hold. Drawdowns are measured relative to each strategy’s own running peak. The Frozen Agent (purple) exhibits dramatically shallower drawdowns (MaxDD −9.0%) compared to Buy-and-Hold (green, MaxDD −18.8%)—a 52% reduction in worst-case losses. The Adaptive Agent (orange) experiences intermediate drawdowns (MaxDD −12.6%). Notable protection occurs during the October 2022 correction and March 2025 market stress, where the Frozen Agent’s uncertainty-triggered position reduction limits losses to approximately half those of Buy-and-Hold.

**Figure A6:**
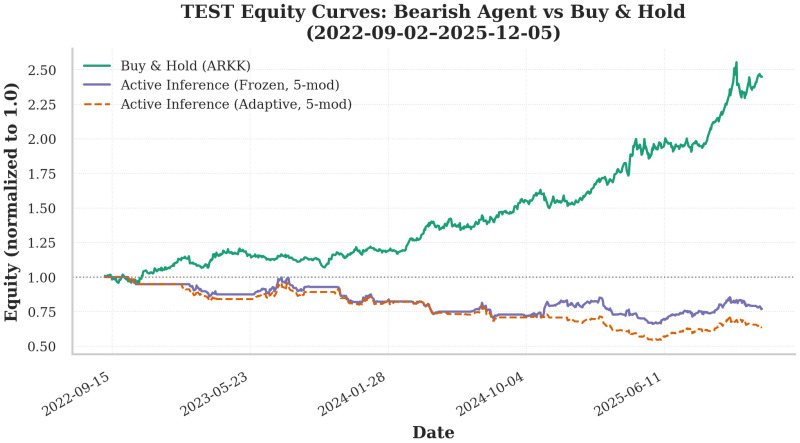
Bearish profile (OOS): Normalized test-period equity curves for Buy-and-Hold ARKK and the Bearish Agent (Frozen vs. Adaptive). Period: 2 September 2022 to 5 December 2025. Curves are normalized to 1.0 at the start of the test window. Performance ratios are risk-free-aware and computed on daily excess returns.

**Figure A7:**
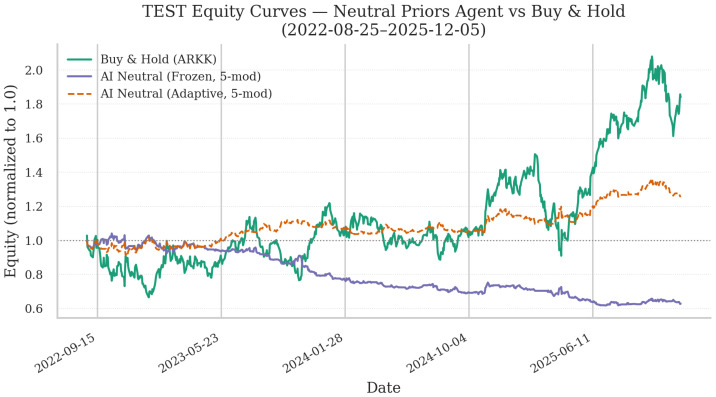
Neutral Agent (OOS): Normalized test-period equity curves for Buy-and-Hold ARKK and the Neutral Adaptive Agent (Frozen vs. Adaptive). Period: 25 August 2022 to 5 December 2025. Curves are normalized to 1.0 at the start of the test window. Performance ratios are risk-free-aware and computed on daily excess returns.

**Figure A8:**
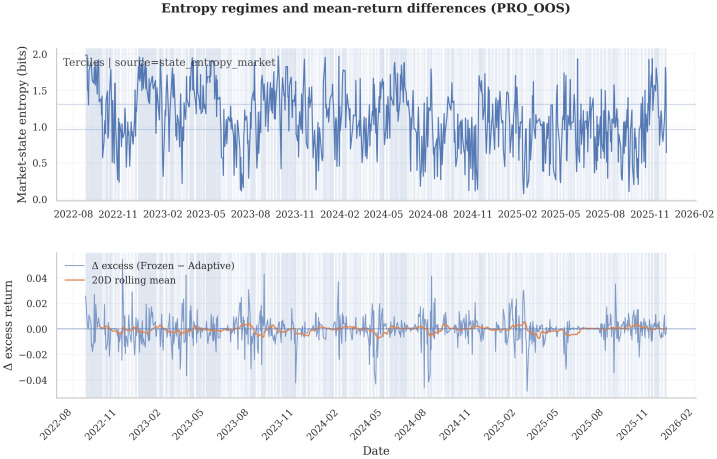
Regime-Conditional Manifestation of the Passivity Paradox (Professional Agent). ***Top** panel*: Market-state entropy $H[Q\left(s_{t}^{m}\right)]$ in bits over the out-of-sample period (26 August 2022 to 5 December 2025, n=823 trading days). Horizontal lines denote tercile thresholds; shaded bands indicate entropy regimes (light: Low, medium: Mid, dark: High). ***Bottom** panel*: Daily excess-return difference $\Delta r_{t}=r_{t}^{\mathrm{Frozen}}-r_{t}^{\mathrm{Adaptive}}$ (blue) with 20-day rolling mean (orange). In low- and mid-entropy regimes, $\Delta r_{t}$ is persistently positive (Frozen dominates), consistent with the Passivity Paradox. In high-entropy regimes, $\Delta r_{t}$ fluctuates around zero with no significant directional bias, explaining why Adaptive achieves lower MSE despite inferior mean returns. This visual evidence reconciles HAC *t*-test results (mean dominance in learnable regimes) with Diebold–Mariano tests (variance efficiency under maximal uncertainty).

**Figure A9:**
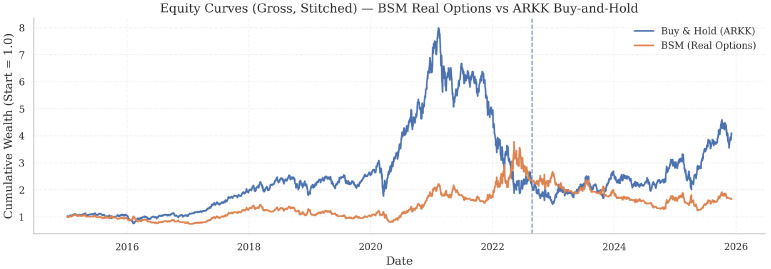
Gross stitched equity curves for the BSM Real Options benchmark and Buy-and-Hold ARKK. The dashed line marks the train–test boundary (train ends 24 August 2022; test begins 26 August 2022). The BSM Real Options benchmark avoids the 2021 peak and mitigates the 2022 drawdown but re-enters slowly during the 2023–2024 recovery, resulting in negative out-of-sample Sharpe ratio.

**Figure A10:**
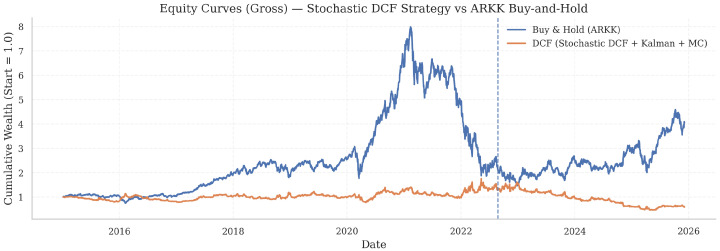
Stochastic DCF execution diagnostics (ARKK, train–test split shown by vertical dashed line). **Top panel:** Net portfolio weight $w_{t}\in \left[-1,+1\right]$ implied by the Stochastic DCF signal. Position size scales with estimated mispricing and is capped to enforce bounded exposure. Decision days correspond to discrete rebalancing events when the valuation signal exceeds execution thresholds. **Bottom panel:** Raw mispricing signal $\left(FV_{t}-P_{t}\right)/P_{t}$ (left axis) together with the Monte Carlo valuation standard error (right axis). The mispricing signal reflects deviations between estimated fundamental value and market price, while the valuation standard error captures dispersion of Monte Carlo draws and therefore model-implied valuation uncertainty. Periods of elevated standard error coincide with reduced position magnitudes, illustrating uncertainty-aware scaling: large valuation dispersion dampens exposure even when point estimates indicate mispricing. Together, the panels show how the strategy converts probabilistic valuation estimates into risk-adjusted position sizing rather than binary trading signals.

**Figure A11:**
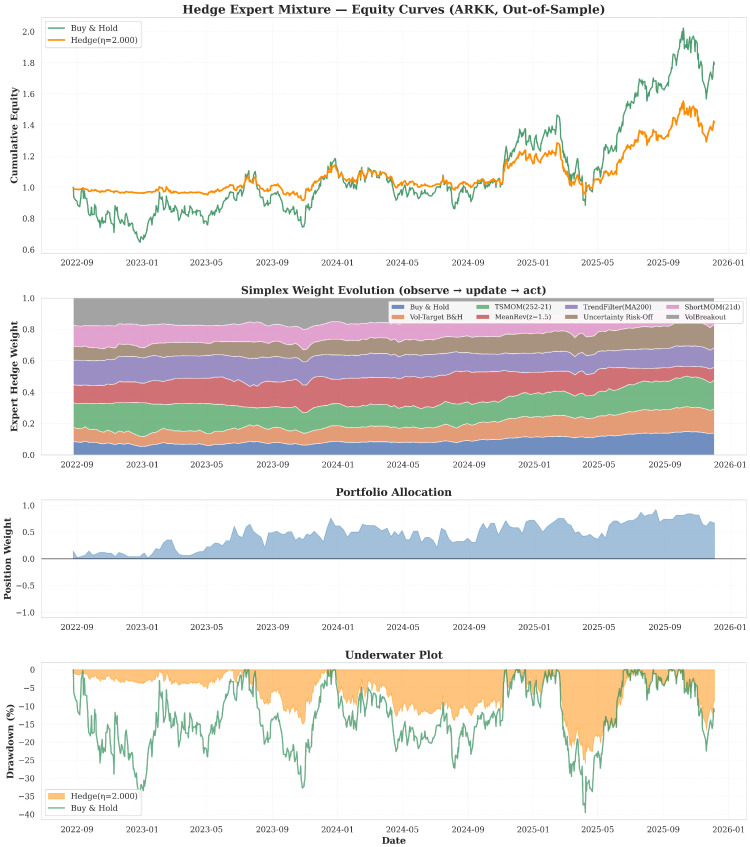
Hedge Expert Mixture—Out-of-sample diagnostics (ARKK, 2022–2025). ***Top** panel:* cumulative equity curves for the Hedge Expert Mixture (orange) and Buy & Hold (green). The mixture moderates both upside and downside relative to full exposure, producing smoother equity growth with reduced drawdown severity. ***Second** panel:* simplex evolution of expert weights (sum to one). The portfolio dynamically reallocates across signal components (trend, momentum, volatility targeting, uncertainty filters, etc.), illustrating adaptive risk rebalancing rather than static exposure. ***Third** panel:* aggregate portfolio allocation (net equity weight). Position size varies continuously between partial exposure and near-full allocation, reflecting regime-sensitive scaling rather than binary long/flat switching. ***Bottom** panel:* underwater plot (drawdowns). The Hedge mixture exhibits shallower and shorter drawdown episodes compared to Buy & Hold, indicating improved downside control despite lower terminal return. Together, the panels show that performance differences arise from systematic risk modulation via expert-weight reallocation and exposure scaling, not from directional timing alone.

## Data Availability

The empirical analysis uses publicly available data from the following sources: (i) daily adjusted close prices and trading volume for the ARK Innovation ETF (ARKK, ticker: ARKK) and the S&P 500 ETF (SPY) were obtained from Yahoo Finance; (ii) daily three-month U.S. Treasury Bill yields (secondary market) were retrieved from Alpha Vantage; (iii) the Equity Risk Premium (ERP) time series used in the Stochastic DCF benchmark was obtained from Aswath Damodaran’s publicly available database and aligned to the trading-day frequency via forward-filling; and (iv) annual capital gains and income distribution data were obtained from official ARK Investment Management LLC fund disclosures. The training period spans 5 January 2015 through 24 August 2022, while the out-of-sample test period spans 25 August 2022 through 5 December 2025. Code and replication materials are not publicly available due to intellectual property and commercialization considerations. To ensure scientific transparency, detailed methodological descriptions are provided in the manuscript and appendices, with code available from the corresponding author upon request for academic verification.

## Appendix A. Parameterization of the Professional Agent

This appendix provides a transparent matrix-level description of the Professional Adaptive Inference Agent. The goal is to make explicit how each component of the underlying Partially Observable Markov Decision Process (POMDP) is specified, why these choices are theoretically grounded, and how they map directly to the implementation used in the experiments. The presentation complements the main methodology by focusing on structure and auditability rather than procedural detail.

### Appendix A.1. State and Observation Spaces

The latent state space factorizes into market and position components

$$s_{t}=\left(s_{t}^{m},s_{t}^{p}\right),\;|s^{m}\left|\;=4,\;\right|s^{p}|\;=3,$$

corresponding to four market regimes (Bull, Moderate, Bear, Crisis) and three portfolio positions (Short, Flat, Long). This factorization is standard in Active Inference, and separates exogenous market dynamics from agent-controlled exposure.

The agent observes five modalities

$$o_{t}=\left(o_{t}^{ret},o_{t}^{pnl},o_{t}^{mom},o_{t}^{vol},o_{t}^{vlm}\right),$$

with cardinalities [4,4,3,3,2] representing discretized returns, realized P&L, momentum, volatility regime, and volume shock, respectively. Only the P&L modality is directly controllable via actions.

### Appendix A.2. Likelihood Model (A-Matrices)

The likelihood factorizes across modalities

$$P\left(o_{t}|s_{t}\right)=\prod_{m}P\left(o_{t}^{\left(m\right)}|s_{t}\right),$$

with the following structure.

Exogenous modalities.

The return, momentum, volatility, and volume likelihoods depend only on the market state $s^{m}$ and are invariant to the position state $s^{p}$. For example, the return likelihood $A^{ret}$ satisfies

$$P\left(o^{ret}|s^{m}=\mathrm{Bull}\right)=\left[0.70,\;0.15,\;0.10,\;0.05\right],\;P\left(o^{ret}|s^{m}=\mathrm{Crisis}\right)=\left[0.02,\;0.05,\;0.10,\;0.60\right],$$

capturing diagonal dominance, fat-tailed downside risk in crisis regimes, and epistemic uncertainty via off-diagonal mass. The momentum, volatility, and volume likelihoods encode empirically established regime correlations [101,102] and are treated as purely informational signals.

Controllable modality.

The P&L likelihood $A^{pnl}$ depends jointly on $\left(s^{m},s^{p}\right)$, encoding the fundamental trading asymmetry that long positions profit in bullish regimes and lose in bearish or crisis regimes, with the reverse holding for short positions. The flat position produces small, near-neutral outcomes across regimes. This construction satisfies the Active Inference controllability principle [48]: only outcomes influenced by actions carry preferences.

### Appendix A.3. Transition Model (***B*** Matrices)

The market transition matrix $B^{m}=P\left(s_{t+1}^{m}|s_{t}^{m}\right)$ is an uncontrolled persistent Markov chain with diagonal dominance (e.g., P(Bull→Bull)=0.85, P(Crisis→Crisis)=0.65), allowing gradual mean reversion and asymmetric crash dynamics consistent with regime-switching models [25].

The position transition matrix $B^{p}=P\left(s_{t+1}^{p}|s_{t}^{p},a_{t}\right)$ is action-conditioned with four actions (Hold, Buy, Sell, Reduce). Transitions are stochastic in order to reflect execution uncertainty and enforce gradual reversals (e.g., sell from Long moves primarily to Flat rather than directly to Short), consistent with the execution constraint requiring de-risking before flipping sign.

### Appendix A.4. Preferences, Priors, and Habits (***C**,**D**,**E***)

Preferences are defined only over P&L outcomes:

$$C^{pnl}=\kappa \cdot \left[+2.0,\;+0.6,\;-0.6,\;-2.0\right]=\left[4.0,\;1.2,\;-1.2,\;-4.0\right]$$

with scaling κ=2.0 and symmetric loss treatment (λ=1.0) reflecting professional institution-like behavior. All other modalities have zero utility.

Initial state priors are uninformative over market regimes and conservative over positions:

$$D^{m}=\mathrm{Uniform},\;D^{p}=\left[0.10,\;0.80,\;0.10\right]$$

biasing the agent towards starting flat. A habit prior over policies penalizes frequent action switches according to

$$E\left(\pi \right)\propto {\left(0.70\right)}^{\#\mathrm{switches}},$$

providing soft regularization against excessive turnover.

### Appendix A.5. Surgical Learning and Auditability

The Professional Agent employs *surgical learning*; parameter adaptation is restricted to the exogenous return likelihood, while all other likelihoods are held fixed. Dirichlet priors encode prior strength separately from learning scope:

$$pA^{ret}\propto 20\cdot A^{ret}\;\left(\eta^{ret}=0.04,\;\omega =0.99\right),\;pA^{pnl}\propto 20\cdot A^{pnl}\;\left(\eta^{pnl}=0\right),\;pA^{aux}\propto 1000\cdot A^{aux}\;\left(\eta^{aux}=0\right).$$

Only $A^{ret}$ is updated online via Bayesian evidence update with forgetting, followed by renormalization. The P&L likelihood is intentionally frozen despite its moderate prior strength to prevent policy-feedback contamination, since P&L observations depend directly on the agent’s own actions. This design ensures Lucas Critique compliance [14]: learning is confined to uncontrollable market signals, while policy-dependent channels remain structurally invariant.

### Appendix A.6. Summary

Together, these choices define a Professional Agent that is epistemically active, risk-aware, and fully auditable at the matrix level. Each component of the POMDP is economically interpretable, theoretically grounded, and maps one-to-one to the implementation used in the empirical analysis.

## Appendix B. Robustness and Sensitivity Analysis

This appendix reports robustness checks for the Professional ARKK Agent only. To avoid mixing estimators across sections, we follow a two-level reporting convention:

- Canonical baseline (paper-wide): Sharpe ratios in the main results are computed on risk-free-aware daily excess returns using the paper’s canonical pipeline. For the standard configuration (COST_BPS =5, MAX_STEP_SIZE =0.40), the canonical Sharpe ratio values are
  $${\mathrm{Sharpe}}_{\mathrm{Frozen}}^{\mathrm{canon}}=0.385533,\;{\mathrm{Sharpe}}_{\mathrm{Adaptive}}^{\mathrm{canon}}=-0.281313.$$
- Sensitivity deltas (within-sweep): Sensitivity tables report ΔSharpe within the sweep’s internal estimator, defined relative to the sweep baseline at MAX_STEP_SIZE =0.40 (and COST_BPS =5 when applicable). This guarantees internal consistency even when the sweep’s estimator differs slightly from the canonical one.

The behavioral parameter sweeps reported in this appendix complement the headline results presented in Section 4.2.5. Robustness is assessed along four dimensions. Three are cognitive dimensions of the agent’s architecture: policy precision (γ, 3/5), epistemic weight (η, 6/6), and loss aversion (λ, 6/7), which together establish that the Passivity Paradox holds across 15 of 18 tested configurations (83.3%). The last *structural* dimension is cross-asset generalizability (Appendix B.6), which confirms that the paradox persists on a fundamentally different return-generating process (SPY, the broad market portfolio). Here, we provide the full metric tables, behavioral decomposition, and mechanistic detail underlying those findings.

The pymdp package implements the expected free energy (EFE) using the canonical formulation in Active Inference, in which the epistemic value enters the policy evaluation with unit weight (η=1 implicitly). As a result, epistemic drive is not independently parameterized in the baseline agent configurations. To assess behavioral sensitivity to epistemic weighting, we introduce a controlled engineering modification in which the epistemic term of the Expected Free Energy is scaled by a coefficient η. This modification is applied only within the sensitivity analysis, and does not affect any baseline results. This design enables meaningful one-at-a-time parameter sweeps while preserving the canonical implementation of pymdp in all primary experiments. This modification is implemented as a multiplicative coefficient on the epistemic component of the expected free energy (EFE) in the policy score G(π), leaving the generative model (A,B,D), preferences C, and execution layer unchanged. Unlike the γ and λ sweeps, which operate through the standard policy-posterior pathway, the η sweep is implemented via an explicit control-layer modification of the EFE objective. Therefore, we interpret this exercise as a controller ablation rather than a strictly isomorphic single-parameter perturbation.

Sensitivity tables report the ΔSharpe ratio within the sweep harness, defined relative to the sweep baseline configuration (MAX_STEP_SIZE =0.40, COST_BPS =5, and baseline cognition $\gamma_{0}=8$, $\eta_{0}=0.30$, $\lambda_{0}=1.0$ unless stated otherwise).

### Appendix B.1. Policy Precision Sensitivity

Table A1 reports the full γ-sweep at fixed epistemic weight ($\eta_{0}=0.30$) and loss aversion ($\lambda_{0}=1.00$), with execution parameters COST_BPS =5 and MAX_STEP_SIZE =0.40. Policy precision controls the sharpness of the softmax over expected free energy (EFE) scores: low γ yields near-uniform policy selection (decisional paralysis), while high γ amplifies small EFE differences into near-deterministic action selection.

**Table A1:** Policy Precision (γ) Sweep (Professional, ARKK OOS 2022–2025). All runs at fixed $\eta_{0}\;=\;0.30$, $\lambda_{0}\;=\;1.00$, COST_BPS =5, MAX_STEP_SIZE =0.40. $\Delta SR\left(\gamma \right)=SR_{\mathrm{frz}}-SR_{\mathrm{adp}}$; positive values indicate the paradox holds. Star (^★^) marks the canonical baseline (γ=8). $m_{\mathrm{act}}$ denotes execution rate (fraction of decision opportunities resulting in a trade).

	Sharpe		Sortino		MaxDD (%)		CAGR (%)		$m_{\mathrm{act}}$ (%)			
γ	Frozen	Adaptive	Frozen	Adaptive	Frozen	Adaptive	Frozen	Adaptive	Frozen	Adaptive	ΔSR	Paradox
3	+0.179	+0.595	+0.279	+0.841	−12.1	−15.8	+6.3	+12.9	3.7	12.2	−0.416	×
5	+0.518	+0.299	+0.739	+0.397	−35.0	−29.0	+17.4	+9.3	19.5	22.6	+0.219	✓
8 ^★^	+0.386	−0.281	+0.532	−0.353	−35.8	−41.3	+12.3	−4.4	27.4	30.5	+0.667	✓
10	−0.055	−0.017	−0.073	−0.022	−44.0	−46.3	+0.1	+0.6	32.9	40.9	−0.038	×
15	−0.275	−1.029	−0.360	−1.234	−43.1	−60.3	−4.9	−20.6	48.2	51.2	+0.754	✓
*Scorecard:*											**3 / 5** (60.0%)	

Interpretation.

Policy precision exhibits a non-monotonic relationship with the Passivity Paradox, revealing three distinct behavioral regimes. At γ=3 (paralysis regime), the softmax over γG is too diffuse to generate confident action selections: the Frozen Agent’s execution rate collapses to 3.7% (6 trades over 824 days), producing a near-passive long-only portfolio that generates only +0.179 Sharpe. The Adaptive Agent outperforms (+0.595) because online $A^{\left(0\right)}$ updates provide enough belief sharpening to partially rescue signal quality at low precision, the only γ level at which adaptive learning adds value.

In the paradox regime (γ∈{5,8,15}), frozen beliefs consistently dominate. At the baseline γ=8, the paradox is achieved ΔSR=+0.667. At γ=15, both agents suffer negative Sharpe ratios, but adaptive learning degrades far more severely (−1.029 vs. −0.275): high precision amplifies noisy $A^{\left(0\right)}$ drift into highly decisive but systematically misaligned position changes, producing −60.3% maximum drawdown and 51.2% execution rate. The peak paradox magnitude ΔSR=+0.754 occurs here precisely because high γ magnifies the damage from adaptive belief corruption.

The notch at γ=10 (ΔSR=−0.038) represents a narrow region in which both agents perform similarly poorly (−0.055 vs. −0.017). This is not a regime where adaptive learning succeeds, but rather one where the Frozen Agent’s performance degrades faster than the Adaptive Agent’s, likely due to overconfident commitment to stale beliefs at this particular precision level.

The execution rate $m_{\mathrm{act}}$ increases monotonically with γ in both modes (Figure A1c), confirming the mechanism of higher policy precision ⇒ sharper σ(γG)⇒ more confident action selections ⇒ higher turnover. At all γ levels, the Adaptive Agent trades more frequently than the Frozen Agent, consistent with online learning introducing posterior volatility that increases the frequency of confidence-gate crossings.

### Appendix B.2. Epistemic Weight Sensitivity

Table A2 reports the η-sweep at fixed policy precision ($\gamma_{0}=8$) and loss aversion ($\lambda_{0}=1.00$). The epistemic weight η scales the information-gain component of expected free energy (EFE): G(π)=utility(π)+η·info_gain(π). At η=0, the agent is purely exploitative; at η=1, the canonical pymdp formulation is recovered.

**Table A2:** Epistemic Weight (η) Sweep (Professional, ARKK OOS 2022–2025). All runs at fixed $\gamma_{0}\;=\;8$, $\lambda_{0}\;=\;1.00$, COST_BPS =5, MAX_STEP_SIZE =0.40. $\Delta SR\left(\eta \right)=SR_{\mathrm{frz}}-SR_{\mathrm{adp}}$; positive values indicate the paradox holds. Dagger (^†^) marks η=1.0, which reproduces the canonical pymdp EFE formulation. All runs use a custom η-weighted EFE at the control layer.

	Sharpe		Sortino		MaxDD (%)		CAGR (%)		$m_{\mathrm{act}}$ (%)			
η	Frozen	Adaptive	Frozen	Adaptive	Frozen	Adaptive	Frozen	Adaptive	Frozen	Adaptive	ΔSR	Paradox
0.00	+0.006	−0.505	+0.007	−0.688	−32.6	−51.3	+1.7	−9.2	40.9	46.3	+0.511	✓
0.10	−0.143	−0.675	−0.184	−0.857	−32.6	−46.8	−2.0	−10.9	38.4	44.5	+0.532	✓
0.25	+0.194	−0.283	+0.259	−0.366	−32.6	−46.2	+6.6	−5.0	36.0	41.5	+0.477	✓
0.50	+0.282	−0.437	+0.380	−0.560	−33.8	−50.0	+9.0	−7.2	31.1	42.1	+0.719	✓
0.75	+0.267	−0.312	+0.364	−0.390	−36.7	−49.9	+8.6	−6.0	27.4	36.6	+0.579	✓
1.00 ^†^	+0.386	−0.281	+0.532	−0.353	−35.8	−41.3	+12.3	−4.4	27.4	30.5	+0.667	✓
*Scorecard:*											**6 / 6** (100%)	

Interpretation.

The epistemic weight sweep yields the most decisive result of the three cognitive dimensions: the Passivity Paradox holds at *every* tested η value, with ΔSR ranging from +0.477 (η=0.25) to +0.719 (η=0.50). This uniformity establishes that the paradox is *not* an artifact of the information-gain term in expected free energy (EFE), as it persists even when epistemic drive is eliminated (η=0).

Two structural patterns emerge. First, the Frozen Agent’s Sharpe ratio improves broadly from +0.006 (η=0) to +0.386 (η=1.0), indicating that epistemic drive acts as a beneficial regularizer for the frozen policy: information-seeking discourages premature commitment to high-confidence positions, reducing turnover from 40.9% (η=0) to 27.4% (η=1.0) and allowing the execution-friction buffer mechanism to operate more effectively.

Second, the Adaptive Agent’s performance is uniformly negative across all η values (worst: −0.675 at η=0.10; best: −0.281 at η=1.0), confirming that surgical $A^{\left(0\right)}$ updates corrupt belief quality regardless of the epistemic–exploitative balance. The peak paradox magnitude at η=0.50 (ΔSR=+0.719) coincides with a substantial execution-rate gap (Frozen 31.1% vs. Adaptive 42.1%), consistent with the mechanism identified in the λ-sweep: the paradox is strongest where trading-frequency divergence between modes is greatest.

The η=0.10 dip in Sharpe (−0.143) for the Frozen Agent without a corresponding improvement for the Adaptive Agent (−0.675) suggests that a small but nonzero epistemic weight can disrupt the purely utilitarian policy gradient without providing sufficient information-seeking regularization, making for a “worst of both worlds” configuration. This dip is localized, and does not affect the universal paradox that holds throughout the sweep.

### Appendix B.3. Loss Aversion Sensitivity

Table A3 reports the full λ-sweep at fixed cognitive baseline ($\gamma_{0}=8$, $\eta_{0}=0.30$, COST_BPS =5, MAX_STEP_SIZE =0.40). Unlike the execution-layer sweeps in Table A4 and Table A5, this sweep modifies the agent’s *preference structure* by rescaling the loss penalties in $C^{\left(\mathrm{PnL}\right)}$ while holding gain rewards fixed. The paradox magnitude $\Delta SR\left(\lambda \right)=SR_{\mathrm{frz}}-SR_{\mathrm{adp}}$ is reported directly, as the comparison of primary interest is cross-mode rather than within-mode.

**Table A3:** Loss Aversion (λ) Sweep (Professional, ARKK OOS 2022–2025). All runs at fixed $\gamma_{0}\;=\;8$, $\eta_{0}\;=\;0.30$, COST_BPS =5, MAX_STEP_SIZE =0.40. $\Delta SR\left(\lambda \right)=SR_{\mathrm{frz}}-SR_{\mathrm{adp}}$; positive values indicate the paradox holds. Star (^★^) marks the canonical baseline (λ=1.00). The single reversal at λ=2.00 marks the preference-destruction boundary where loss penalties exceed twice the gain rewards.

	Sharpe		Sortino		MaxDD (%)		CAGR (%)			
λ	Frozen	Adaptive	Frozen	Adaptive	Frozen	Adaptive	Frozen	Adaptive	ΔSR	Paradox
0.75	+0.464	−0.252	+0.665	−0.322	−32.9	−40.1	+15.3	−4.2	+0.716	✓
1.00 ^★^	+0.386	−0.281	+0.532	−0.353	−35.8	−41.3	+12.3	−4.4	+0.667	✓
1.25	+0.079	−0.446	+0.107	−0.576	−37.8	−45.2	+3.6	−8.9	+0.525	✓
1.50	−0.243	−0.532	−0.312	−0.686	−36.7	−45.8	−3.6	−10.1	+0.290	✓
1.75	−0.322	−0.385	−0.417	−0.515	−36.8	−46.8	−4.7	−6.7	+0.063	✓
2.00	−0.821	−0.280	−1.034	−0.374	−47.6	−41.0	−14.4	−4.1	−0.541	×
2.25	−0.160	−0.280	−0.210	−0.376	−32.9	−37.2	−1.0	−3.6	+0.120	✓
*Scorecard:*									**6 / 7** (85.7%)	

Interpretation.

Loss aversion monotonically degrades both absolute performance and paradox magnitude across the range λ∈[0.75,2.00]. The Frozen Agent’s Sharpe ratio declines from +0.464 (λ=0.75) through the canonical baseline +0.386 (λ=1.00) to −0.821 at the preference-destruction boundary (λ=2.00), while the Adaptive Agent exhibits less sensitivity (range [−0.532,−0.252]), consistent with adaptive learning partially compensating for preference misspecification.

The paradox holds at six of seven tested configurations (85.7%). At low loss aversion (λ≤1.25), the Frozen Agent substantially outperforms (ΔSR>+0.5), driven by decisive long positioning that captures the equity risk premium without the noise amplification introduced by online $A^{\left(0\right)}$ updates. As λ increases, loss penalties progressively suppress long exposure (Long% declines from 73% to 44%; see Figure A2b), eroding the Frozen Agent’s advantage while the Adaptive Agent’s performance remains roughly flat.

The Frozen Sharpe ratio declines strictly monotonically over λ∈[0.75,2.00], indicating that increasing loss aversion progressively reduces effective risk exposure under fixed beliefs. Therefore, the prominent deterioration observed at λ=2.00 represents the terminal point of a continuous contraction rather than an isolated anomaly.

The single reversal at λ=2.00 (ΔSR=−0.541) marks a structurally distinct regime: at this level, loss penalties in $C^{\left(\mathrm{PnL}\right)}$ exceed twice the gain rewards, effectively destroying the preference gradient that enables profitable risk-taking. The Frozen Agent’s drawdown deepens to −47.6% (vs. −41.0% for Adaptive), and CAGR collapses to −14.4%. Recovery at λ=2.25 (ΔSR=+0.120) suggests that this reversal is a localized resonance rather than a monotonic phase transition. Execution rate convergence at high λ (Figure A2c) confirms the mechanistic interpretation: the paradox is strongest where the execution-rate gap between modes is widest and is weakest where both agents converge to similar trading frequencies.

### Appendix B.4. Execution Speed Sensitivity

Table A4 varies MAX_STEP_SIZE while holding all other parameters fixed. The ΔSharpe ratio is computed within the sweep relative to MAX_STEP_SIZE =0.40 for each mode.

**Table A4:** Execution Speed Sensitivity (Professional, ARKK OOS 2022–2025). Canonical baseline Sharpe ratio at (COST_BPS =5, MAX_STEP_SIZE =0.40) is 0.385533 (Frozen) and −0.281313 (Adaptive). This table reports *within-sweep* ΔSharpe relative to MAX_STEP_SIZE =0.40 for each mode, along with associated drawdown and execution diagnostics.

Mode	MAX_STEP_SIZE	ΔSharpe (Sweep)	MaxDD	CAGR	Turnover	Est. Cost Drag
Frozen	0.40	0.0000	−35.8%	+12.3%	14.44	0.72%
Frozen	0.60	−0.4191	−42.2%	+0.2%	16.65	0.83%
Frozen	0.80	−0.5500	−44.6%	−2.7%	19.69	0.98%
Adaptive	0.40	0.0000	−41.3%	−4.4%	17.74	0.89%
Adaptive	0.60	+0.0153	−43.5%	−4.3%	22.37	1.12%
Adaptive	0.80	+0.0531	−37.6%	−3.5%	27.37	1.37%

Interpretation.

Execution speed critically affects the Frozen Agent’s performance through the interaction of turnover and timing. In Frozen mode, MAX_STEP_SIZE =0.40 is optimal: increasing execution speed to 0.60 or 0.80 degrades Sharpe ratio by −0.42 and −0.55, respectively, deepens drawdowns, and increases turnover. This supports the paper’s “execution friction as uncertainty buffer” thesis, demonstrating that slower rebalancing provides robustness against belief–action misalignment during regime transitions.

The Adaptive Agent’s performance is uniformly negative across all step sizes, though a marginal improvement (+0.05 Sharpe at MAX_STEP_SIZE =0.80) does not overturn the fundamental finding that adaptive learning harms performance. Notably, the Adaptive Agent’s turnover increases substantially with execution speed (17.74 to 27.37 units), amplifying cost drag without corresponding improvement in returns.

### Appendix B.5. Transaction Cost Sensitivity

Table A5 varies transaction costs (COST_BPS) at the standard MAX_STEP_SIZE =0.40. ΔSharpe is computed within the sweep relative to COST_BPS =5 for each mode.

**Table A5:** Transaction Cost Sensitivity (Professional, ARKK OOS 2022–2025; MAX_STEP_SIZE =0.40). Canonical baseline Sharpe ratio at (COST_BPS =5, MAX_STEP_SIZE =0.40) is 0.385533 (Frozen Agent) and −0.281313 (Adaptive Agent). This table reports *within-sweep* ΔSharpe ratio relative to COST_BPS =5 for each mode, holding the execution configuration fixed.

Mode	COST_BPS	ΔSharpe (Sweep)	MaxDD	CAGR	Turnover	Est. Cost Drag
Frozen	1	+0.0062	−35.6%	+12.5%	14.44	0.14%
Frozen	5	0.0000	−35.8%	+12.3%	14.44	0.72%
Frozen	10	−0.0078	−35.9%	+12.1%	14.44	1.44%
Adaptive	1	+0.0093	−40.9%	−4.2%	17.74	0.18%
Adaptive	5	0.0000	−41.3%	−4.4%	17.74	0.89%
Adaptive	10	−0.0117	−41.7%	−4.7%	17.74	1.77%

Interpretation.

Both learning modes exhibit linear and modest sensitivity to transaction costs in the tested range. The Frozen Agent’s performance degrades by approximately 0.008 Sharpe per 5 bps increase while that of the Adaptive Agent degrades by approximately 0.011 Sharpe per 5 bps, consistent with Adaptive Agent’s higher turnover (17.74 vs. 14.44 units). Crucially, turnover remains constant across cost levels within each mode, confirming that transaction costs are applied post hoc and do not influence execution decisions.

The Passivity Paradox persists across all cost levels: the Frozen–Adaptive Agents gap is +0.664 at 1 bps, +0.667 at 5 bps, and +0.671 at 10 bps. The slight widening at higher costs reflects Frozen Agent’s turnover advantage compounding with the cost multiplier. Combined with the parameter-space robustness reported in Figure A1 and Figure A2 (15/18 configurations, 83.3%), these results confirm that the paradox is not an artifact of the baseline cost assumption.

### Appendix B.6. Cross-Asset Structural Robustness

The behavioral parameter sweeps reported above establish that the Passivity Paradox is robust against perturbations of the agent’s cognitive architecture *within* a fixed return-generating process (ARKK). A more demanding test is whether the paradox survives when the return-generating process itself changes. ARKK is a concentrated thematic ETF (disruptive innovation) exhibiting high idiosyncratic volatility (∼50% annualized), extreme drawdown (−65% peak-to-trough in 2022), and pronounced regime-switching dynamics. A skeptical reading of the ARKK results might attribute the Frozen Agent’s advantage to passive long exposure during a mean-reversion recovery that the Adaptive Agent overreacts to—an asset-specific timing story rather than a structural property of Active Inference.

To address this, we replicated the full experimental pipeline on SPY, which tracks the S&P 500 and represents the broad market portfolio. This is a protocol replication—the Professional Agent is re-trained on SPY’s own price history using the same training window structure—rather than a cross-asset transfer of ARKK-learned parameters. SPY differs from ARKK across every dimension relevant to the paradox mechanism: lower annualized volatility (16.8% vs. ∼50%), shallower maximum drawdown (−18.8% vs. −65%), diversification across 500+ constituents rather than ∼35 thematic holdings, and a smoother trending profile with less extreme regime transitions. All agent hyperparameters—policy structure, confidence gates, execution cadence (TRADE_STEP =5), transaction costs (COST_BPS =5), step size (MAX_STEP_SIZE =0.40), and risk management (TARGET_VOL =0.40)—are held identical to the ARKK experiments. The full SPY results, including equity curves, position dynamics, and execution diagnostics, are reported in Appendix B.7.

**Table A6:** Cross-Asset Passivity Paradox (Professional Agent, OOS 2022–2025). $\Delta SR=SR_{\mathrm{frz}}-SR_{\mathrm{adp}}$. Both assets use identical agent hyperparameters. ARKK represents a concentrated high-volatility thematic ETF; SPY represents the diversified broad market portfolio. The paradox direction is preserved across both assets, with attenuated magnitude on the lower-volatility instrument.

Asset	Characteristics	${\mathrm{SR}}_{\mathrm{frz}}$	${\mathrm{SR}}_{\mathrm{adp}}$	ΔSR	${\mathrm{MaxDD}}_{\mathrm{frz}}$	Paradox
ARKK	High-vol, concentrated, thematic	+0.386	−0.281	+0.667	−35.8%	✓ Strong
SPY	Low-vol, diversified, broad market	+0.911	+0.668	+0.242	−9.0%	✓ Moderate

Interpretation.

Table A6 confirms that the Passivity Paradox is not an artifact of ARKK’s extreme return dynamics. On SPY, the Frozen Agent outperforms the Adaptive Agent by +0.24 Sharpe units—smaller than the ARKK gap (+0.67), but unambiguously in the same direction. The magnitude attenuation is itself informative: SPY’s lower volatility provides less “noise surface” for surgical $A^{\left(0\right)}$ updates to corrupt beliefs through policy-contingent feedback. Mechanistically, lower volatility reduces the magnitude and frequency of surprise-weighted Dirichlet updates to the return likelihood $A^{\left(0\right)}$, dampening policy-contingent feedback loops that can amplify posterior drift under adaptation. In the limiting case of a perfectly stable return-generating process, adaptive and frozen beliefs would converge and the paradox should attenuate and potentially vanish, consistent with the observed scaling.

The cross-asset comparison also reveals qualitatively different value propositions for frozen inference across volatility regimes. On ARKK, the Frozen Agent underperforms Buy-and-Hold on absolute Sharpe (+0.386 vs. the equity’s raw return profile) but provides substantial tail-risk protection. On SPY, the Frozen Agent achieves comparable risk-adjusted returns to Buy-and-Hold (Sharpe 0.911 vs. 0.889) while reducing maximum drawdown by 52% (from −18.8% to −9.0%). In both cases, the Frozen Agent dominates the Adaptive Agent, confirming that the paradox reflects a structural property of online belief updating under partial observability rather than an asset-specific phenomenon.

Combined with the behavioral parameter sweeps (15/18 configurations on ARKK; 83.3%), the cross-asset validation extends the paradox’s empirical support to a distinct return-generating process and volatility regime. The two assets share a common OOS calendar and macroeconomic environment but differ in sector composition, volatility regime, and return-generating dynamics, precisely the dimensions along which a fragile result would fail to replicate.

### Appendix B.7. Cross-Asset Validation: Full SPY Results

This section reports the full out-of-sample (OOS) results of the Professional Agent trained and evaluated on the SPY ETF. All hyperparameters are identical to the ARKK experiments, including the policy structure, confidence gates, execution cadence, transaction costs, and risk management settings. In particular, both ARKK and SPY use MAX_STEP_SIZE = 0.40, TARGET_VOL = 0.40, and TRADE_STEP = 5, ensuring strict cross-asset comparability.

**Table A7:** Out-of-Sample Performance Summary—SPY (25 August 2022–5 December 2025). The Professional Frozen Agent achieves comparable risk-adjusted returns (Sharpe ratio of 0.91) compared to Buy-and-Hold (Sharpe ratio of 0.89) while reducing maximum drawdown by 52% (from −18.8% to −9.0%). The Passivity Paradox is confirmed: the Frozen Agent outperforms the Adaptive Agent by +0.24 Sharpe ratio units.

Strategy	Sharpe	Sortino	Max Drawdown	CAGR	Ann. Volatility
Buy & Hold (SPY)	0.889	1.325	−18.8%	19.8%	16.8%
Professional (Frozen Agent)	0.991	1.219	−9.0%	15.0%	10.8%
Professional (Adaptive Agent)	0.668	0.888	−12.6%	12.4%	11.5%

Experimental setup.

This section evaluates protocol replication rather than cross-asset transfer: we re-train the Professional Agent on SPY using the same training window structure and evaluate on the same OOS calendar while holding all hyperparameters fixed. The Professional Agent is trained on the SPY ETF series over the period January 2015–August 2022 (1924 trading days) using surgical learning (concentration parameter updates to the return likelihood matrix $A^{\left(0\right)}$), then evaluated out-of-sample from August 2022 to December 2025 (824 trading days, spanning 3.28 years). SPY data are anchored to ARKK’s exact trading calendar to ensure cross-asset comparability. Two variants of OOS are considered: (i) a fully Frozen Agent with no belief updates ($\eta_{A}=0$), and (ii) an Adaptive Agent with continued surgical learning of the return likelihood matrix $A^{\left(0\right)}$ ($\eta_{A}=0.04$). Performance metrics are computed on daily excess returns relative to the three-month Treasury Bill rate (annualized 4.69% during the test period).

Tail-risk management and equity dynamics.

Figure A3 reports the normalized test-period equity curves for Buy-and-Hold SPY and the two Professional Adaptive Inference variants. The central result on SPY is not return enhancement but dramatic *tail-risk reduction*: the Frozen Agent compresses maximum drawdown by 52%, from −18.8% (Buy-and-Hold) to −9.0% while maintaining comparable risk-adjusted returns (Sharpe 0.91 vs. 0.89). This drawdown reduction is the dominant source of value on a low-volatility broad market asset, where return enhancement opportunities are structurally limited by diversification.

The position allocation heatmap (Figure A4) reveals the mechanism: the agent maintains a latent Long state 69.7% of the time (Table A9), while realized exposure remains positive for 92.2% of days due to volatility scaling, confidence gating, and execution friction. During high-entropy periods the agent tactically reduces positions, implementing what we term “uncertainty-aware passive investing”. Because position reductions are triggered by epistemic uncertainty (entropy) rather than predicted negative returns, the strategy functions as an uncertainty-sensitive exposure controller, not a directional forecaster. The underwater dynamics (Figure A5) show that the Frozen Agent avoids the severe March 2025 correction that pushes Buy-and-Hold to nearly −19% underwater. Annualized volatility contracts from 16.8% to 10.8% (a 36% reduction), confirming that inference-driven risk management operates primarily through exposure modulation rather than directional forecasting.

**Table A8:** Execution Metrics Comparison—SPY vs. ARKK. The SPY Professional Frozen Agent executes only 10.7% of opportunities (vs. 26.0% for ARKK), reflecting higher confidence in maintaining positions under stable return dynamics. Lower turnover contributes to superior net performance through reduced transaction costs.

Asset	Mode	Opportunities	Executions	Exec Rate	Turnover
ARKK	Frozen Agent	173	45	26.0%	14.44
ARKK	Adaptive Agent	168	50	29.8%	17.74
SPY	Frozen Agent	197	21	10.7%	8.20
SPY	Adaptive Agent	192	31	16.1%	12.20

Latent beliefs versus realized exposure.

Table A9 reports the distribution of the agent’s latent position state $s_{t}^{\left(p\right)}$, which represents the agent’s internal belief about its intended position category. This three-state representation (Short/Flat/Long) captures the agent’s categorical intention before execution-layer transformations.

Realized portfolio weights differ from latent states due to three mechanisms:

1. Volatility scaling: Target volatility constraints (40% annualized) scale position sizes based on realized market volatility, often resulting in fractional weights even when the latent state indicates full Long (+1) or Short (−1).
2. Confidence gating: Positions are executed only when policy entropy falls below threshold ($H\leq 0.96\cdot H_{\max}$) and maximum policy probability exceeds minimum ($p_{\max}\geq 0.22$). When gates are closed, weights remain unchanged.
3. Execution friction: Maximum step sizes (0.40 out-of-sample), minimum change thresholds ($\delta_{\min}=0.05$), and the through-flat constraint prevent instantaneous position reversals.

For ARKK, the Professional Frozen Agent maintains a Long latent state 65.0% of trading days, but the realized portfolio weight is positive 81.9% of the time (Figure 9). This discrepancy arises because volatility scaling produces fractional positive weights even when the latent state transitions through Flat, and the through-Flat constraint prevents direct Short→Long reversals, keeping the weight non-negative during transitions.

For SPY, the Frozen Agent exhibits even higher realized long exposure (92.2%) despite a latent Long state frequency of 69.7%, reflecting SPY’s lower volatility environment where scaling constraints bind less frequently.

**Table A9:** Latent Position State Distribution—SPY vs. ARKK. Distribution of the agent’s internal position state $s_{t}^{\left(p\right)}\in \left\{\mathrm{Short},\mathrm{Flat},\mathrm{Long}\right\}$ during out-of-sample evaluation. Both assets maintain predominantly long beliefs, but SPY exhibits higher long-state frequency (69.7% vs. 65.0% for ARKK Frozen Agent) and lower short-state frequency (12.3% vs. 16.3%), consistent with SPY’s lower volatility and stronger trending behavior during the test period. Note: These percentages reflect the agent’s intended position beliefs, which differ from realized portfolio weights due to volatility scaling, confidence gating, and execution friction (see Figure 9 and Figure A4 for realized weight dynamics).

Asset	Mode	Short	Flat	Long
ARKK	Frozen Agent	16.3%	18.7%	65.0%
ARKK	Adaptive Agent	12.3%	22.1%	65.6%
SPY	Frozen Agent	12.3%	18.0%	69.7%
SPY	Adaptive Agent	7.9%	18.3%	73.8%

Cross-asset factor co-movement (not leakage).

Because ARKK and SPY ETFs share broad market exposure, positive correlation in strategy P&Ls is expected. We report correlations (Frozen: 0.69; Adaptive: 0.61) to document factor co-movement rather than to claim statistical independence. The robustness claim rests on protocol replication on a distinct instrument: identical code, hyperparameters, and OOS calendar but a different return-generating process. No ARKK-trained parameters are transferred to the SPY agent; each asset is trained and evaluated independently.

Interpretation.

The SPY validation reveals that frozen Adaptive Inference provides qualitatively different value depending on the volatility regime of the underlying asset.

On SPY (low-volatility, diversified), the primary value proposition is *tail-risk management*: the Frozen Agent achieves comparable risk-adjusted returns to Buy-and-Hold while halving maximum drawdown. This profile, consisting of similar upside with dramatically reduced downside, is precisely what institutional allocators seek in risk-overlay strategies: the agent does not attempt to outpredict the broad market but instead withdraws exposure during periods of elevated epistemic uncertainty, providing a form of endogenous portfolio insurance.

On ARKK (high-volatility, concentrated), the value proposition shifts toward *regime navigation*: the Frozen Agent underperforms Buy-and-Hold on absolute returns but achieves positive risk-adjusted performance in a period where the Adaptive Agent generates a negative Sharpe ratio, demonstrating the capacity to maintain profitable positioning through extreme regime transitions without the belief corruption introduced by online learning.

In both regimes, the Passivity Paradox holds: the Frozen Agent dominates the Adaptive Agent by +0.24 Sharpe units on SPY and +0.67 on ARKK. The magnitude attenuation on SPY is consistent with the “noise surface” hypothesis: lower volatility provides fewer opportunities for surgical $A^{\left(0\right)}$ updates to distort beliefs through policy-contingent feedback. The consistent direction across structurally independent assets provides evidence that the paradox reflects a fundamental property of online belief updating under partial observability, not an artifact of any single return-generating process.

## Appendix C. Complete Results by Cognitive Profile

### Appendix C.1. Bearish Profile (OOS Only)

Setup and reporting convention.

This appendix subsection reports the complete out-of-sample (OOS) results for the *Bearish* cognitive profile under two evaluation modes: (i) a fully Frozen Agent (no learning OOS), and (ii) an Adaptive Agent with continued surgical learning of the return-likelihood modality only (i.e., updates restricted to $A^{\left(0\right)}$ while $A^{\left(1\right)},\ldots ,A^{\left(4\right)}$ remain effectively fixed). All reported Sharpe and Sortino ratios are computed on daily excess returns relative to the test-period risk-free rate series (rf_test_daily).

**Table A10:** Bearish Agent: Out-of-Sample Summary (ARKK, 25 August 2022–5 December 2025; risk-free-aware).

Strategy	AnnRet	AnnVol	Sharpe	Sortino	MaxDD	CAGR
Buy & Hold (ARKK)	23.14%	42.98%	0.54	0.80	−39.56%	20.51%
Adaptive Inference (Bearish, Frozen)	−11.38%	16.13%	−0.71	−0.90	−33.90%	−7.68%
Adaptive Inference (Bearish, Adaptive)	−17.27%	15.23%	−1.13	−1.41	−45.79%	−12.84%

Interpretation.

Despite achieving substantially lower realized volatility than Buy-and-Hold ARKK, the Bearish Agent fails to generate positive excess performance out-of-sample. The Frozen Agent attains the smaller drawdown of the two Adaptive Inference runs (MaxDD =−33.9%), while the Adaptive Agent deteriorates further in risk-adjusted terms (Sharpe =−1.13). This pattern is consistent with out-of-sample updates to the return-likelihood matrix $A^{\left(0\right)}$ that do not improve calibration under the prevailing market structure, as reflected by increased turnover and weaker risk-adjusted returns.

Importantly, this negative result is informative rather than anomalous. It indicates that the Adaptive Inference framework does not mechanically produce alpha and that cognitive priors such as loss aversion, epistemic weighting, and policy precision materially shape transfer outcomes. In environments where directional volatility is high but regime persistence is limited, continued epistemic updating may fail to add value and can instead reduce performance relative to a frozen specification.

These findings delineate the operational boundaries of the Bearish Agent and reinforce the role of cognitive configuration in determining out-of-sample robustness.

### Appendix C.2. Neutral Profile (OOS Only)

Setup and reporting convention.

This subsection reports out-of-sample (OOS) results for the *Neutral* cognitive profile under two evaluation modes: (i) a fully frozen agent (no learning OOS), and (ii) an adaptive agent with continued learning in selected observation modalities. In the adaptive configuration, updates are enabled for the return likelihood $A^{\left(0\right)}$ and auxiliary modalities (e.g., momentum/volatility/volume), while the P&L likelihood remains effectively fixed. All reported Sharpe and Sortino ratios are computed on daily excess returns relative to the test-period risk-free rate series (rf_test_daily), with transaction costs applied as weight-change costs in basis points.

**Table A11:** Neutral Agent: Out-of-Sample Summary (ARKK, 25 August 2022–5 December 2025; risk-free-aware).

Strategy	AnnRet	AnnVol	Sharpe	Sortino	MaxDD	CAGR
Buy & Hold (ARKK)	23.14%	42.98%	0.54	0.80	−39.56%	20.51%
Adaptive Inference (Neutral, Frozen)	−18.07%	12.53%	−1.44	−1.76	−40.59%	−13.21%
Adaptive Inference (Neutral, Adaptive)	3.29%	13.23%	0.25	0.31	−8.81%	7.35%

Interpretation.

The Neutral Agent exhibits a stark mode dependence out-of-sample. While the Frozen Agent underperforms materially (Sharpe ratio of =−1.44), the Adaptive Agent recovers to positive risk-adjusted performance (Sharpe ratio of ≈0.25) and, notably, substantially reduces drawdown (MaxDD ≈−8.8%). This pattern is consistent with the intended design of the Neutral Agent specification: precision-weighted inference with continued learning in selected modalities can improve calibration to the post-2022 regime while preserving economic realism (risk-free-aware excess returns, transaction costs, and execution cadence).

## Appendix D. Algorithmic Details and Auditability

This appendix specifies the Active Inference portfolio agent as an *auditable decision system*. All algorithms are presented in execution order and fixed in placement to ensure readability and reproducibility. The implementation follows the discrete-state Active Inference formalism of the Free Energy Principle and is implemented using the open-source pymdp Python library [100], with extensions for portfolio execution, transaction costs, and risk-aware gating.

Implementation note.All probabilistic inference, expected free energy (EFE) evaluation, and Dirichlet learning updates correspond exactly to the reference pymdp.agent.Agent class, with custom wrappers to: (i) enforce action–observation timing consistency, (ii) restrict online learning to selected modalities, and (iii) log sufficient statistics for full post hoc reconstruction. Random seeds are fixed and logged in all experiments.

Conventions and timing.

Time is indexed by t=1,…,T at a daily frequency. The agent observes a multimodal signal

$$o_{t}=\left(o_{t}^{\mathrm{ret}},o_{t}^{\mathrm{pnl}},o_{t}^{\mathrm{mom}},o_{t}^{\mathrm{vol}},o_{t}^{\mathrm{vlm}}\right)$$

constructed exclusively from information available up to day *t*. Hidden states factorize into market regime $s_{t}^{m}$ and position state $s_{t}^{p}$. Decisions are evaluated every $\tau_{\mathrm{exec}}$ trading days; portfolio weights are held constant between decision times except for bounded execution steps. The likelihood factorizes across modalities

$$p\left(o_{t}|s_{t}^{m},s_{t}^{p}\right)=\prod_{m\in M}p\left(o_{t}^{\left(m\right)}|s_{t}^{m},s_{t}^{p}\right).$$

Frozen vs. Adaptive learning.

Frozen mode sets $\eta^{\left(m\right)}=0$ for all modalities, disabling online parameter updates. Adaptive mode enables surgical learning by selecting a strict subset of modalities with $\eta^{\left(m\right)}>0$. In all experiments, the P&L observation channel is treated as policy-contingent and is frozen by design: $\eta^{\mathrm{pnl}}=0$. This prevents mechanical reinforcement of realized returns and eliminates look-ahead bias.

### Appendix D.1. Main Decision Loop

**Algorithm A1:** Adaptive Inference Portfolio Agent (Main Loop)

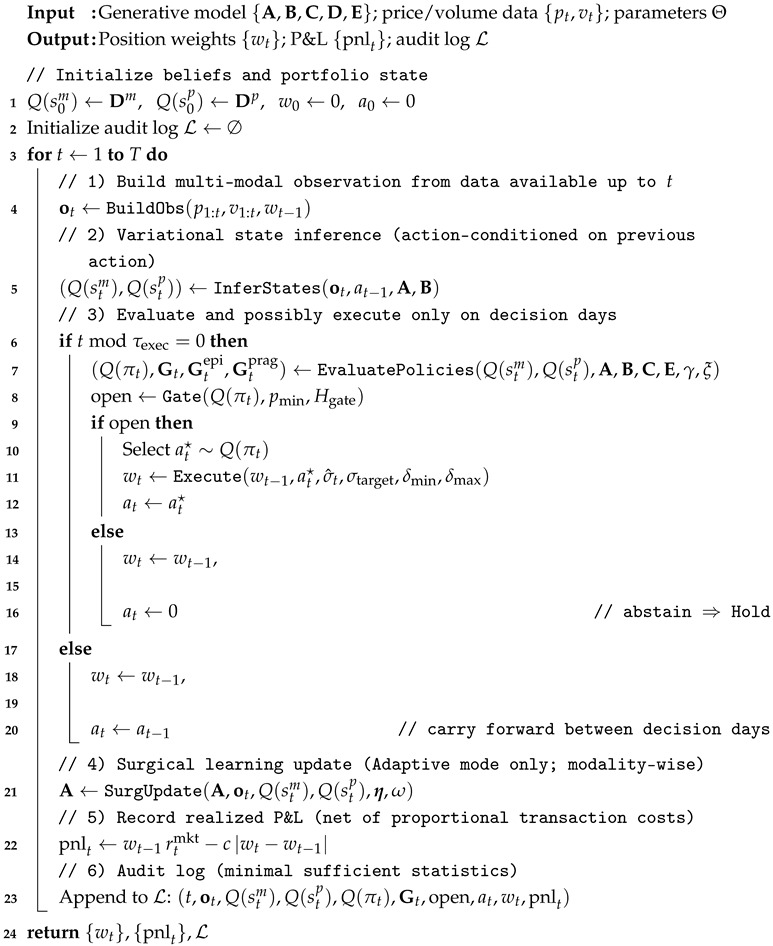

### Appendix D.2. Variational State Inference

**Algorithm A2:** Variational State Inference (Mean-field)

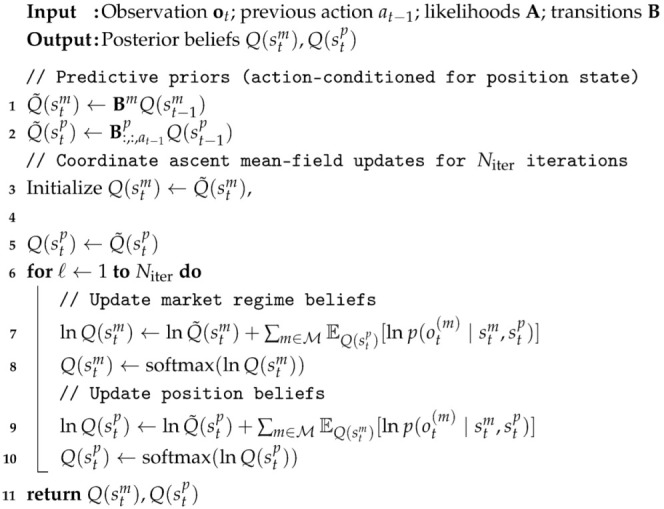

### Appendix D.3. Policy Evaluation via Expected Free Energy

Policy selection follows the standard discrete Active Inference formulation implemented in the pymdp library [100]. This section provides a specification-level description sufficient for auditability while omitting implementation details that are unchanged from the reference framework.

**Algorithm A3:** Policy Evaluation via Expected Free Energy (Specification Level)

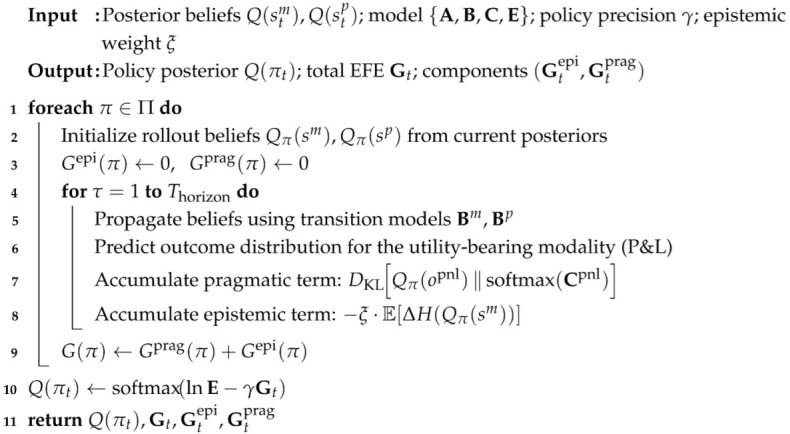

### Appendix D.4. Confidence Gating and Execution

**Algorithm A4:** Confidence Gating and Bounded Execution

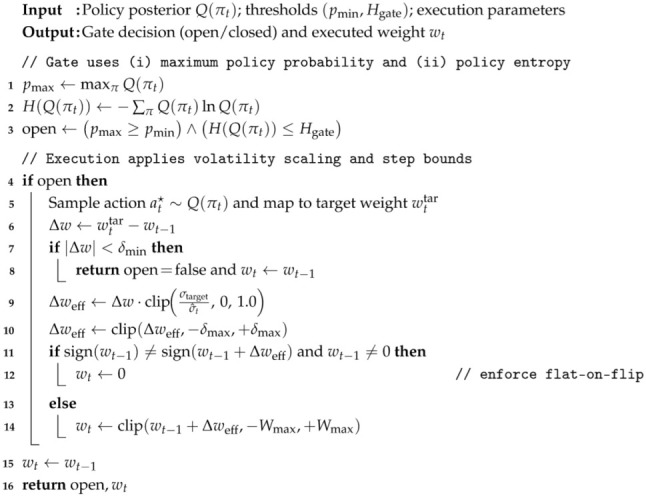

Auditability and reproducibility.

At each decision time *t* (i.e., when $t\;\mathrm{mod}\;\tau_{\mathrm{exec}}=0$), the agent logs (i) the full observation vector $o_{t}$, (ii) posterior beliefs $Q\left(s_{t}^{m}\right),Q\left(s_{t}^{p}\right)$ and their entropies, (iii) the policy posterior $Q(\pi_{t})$, including $p_{\max}$ and $H(Q\left(\pi_{t}\right))$, (iv) the expected free energy (EFE) totals $G_{t}$ and components $\left(G_{t}^{\mathrm{epi}},G_{t}^{\mathrm{prag}}\right)$, (v) the gate decision (open/closed), (vi) the selected action and executed portfolio weight, and (vii) the realized P&L net of proportional transaction costs. Together with the fixed random seed and generative model parameters, these logs are sufficient to reconstruct every portfolio decision as a deterministic function of observed data and model beliefs.

### Appendix D.5. Canonical Parameters for the Professional Agent

**Table A12:** Professional Agent: Global invariant parameters.

Symbol	Description	Value	Notes
$\tau_{\mathrm{exec}}$	Decision cadence	5 days	Weekly execution and inference
FREQ	Trading frequency	252	Annualization constant
TC	Transaction cost	5 bps	Applied to $|w_{t}-w_{t-1}|$
$W_{\max}$	Weight cap	1.0	Long/short exposure bound
γ	Policy precision	8.0	Sharpening of policy posterior
α	Action precision	4.0	Control determinism
η	Epistemic weight	1.0	Information gain term in EFE
λ	Loss aversion	1.0	Symmetric utility (PRO profile)
$c_{\mathrm{scale}}$	Utility scale	2.0	P&L preference scaling
$\sigma_{\mathrm{target}}$	Target volatility	40%	Annualized volatility target
$\delta_{\min}$	Min weight change	0.05	No-op filter
$E_{\mathrm{switch}}$	Habit penalty	0.70	Regime switching regularization

**Table A13:** Professional Agent: Training vs. Frozen test parameters.

Parameter	Description	Train	Test (Frozen)	Notes
$W_{\mathrm{step}}^{\max}$	Max execution step	0.60	0.40	Reduced OOS to limit turnover
$\eta_{\mathrm{fast}}$	Learning rate (return modality $A^{\left(0\right)}$)	0.04	0.00	Surgical learning disabled OOS
$\omega_{\mathrm{fast}}$	Forgetting factor (return modality)	0.99	1.00	No decay in frozen test
$\eta_{\mathrm{slow}}$	Learning rate (other modalities)	0.00	0.00	Always frozen by design
$\omega_{\mathrm{slow}}$	Forgetting factor (other modalities)	1.00	1.00	Structural invariance
$\sigma_{\mathrm{obs}}$	Observation noise scale	0.01	0.01	Identical across regimes
$H_{\mathrm{gate}}/H_{\max}$	Entropy gate ratio	0.96	0.96	Confidence threshold
$p_{\min}$	Minimum policy probability	0.22	0.22	Execution gate

**Table A14:** Professional Agent: Adaptive test parameters.

Parameter	Description	Value	Notes
$\eta_{\mathrm{fast}}$	OOS learning rate	0.04	Surgical learning on $A^{\left(0\right)}$ only
$\omega_{\mathrm{fast}}$	Forgetting factor	0.99	Finite memory (≈60 days)
Learning scope	Modalities updated	$A^{\left(0\right)}$ only	Return likelihood only
Random seed	Determinism control	42	Identical across all runs

### Appendix D.6. Economic Rationale for the Professional Agent Parameterization

The Professional Agent is designed to approximate the behavior of a risk-aware and institutionally-constrained portfolio manager operating under realistic execution frictions, uncertainty, and capital preservation objectives. Its parameterization reflects economic considerations rather than performance-driven tuning.

Decision cadence and execution realism.

The weekly decision cadence ($\tau_{\mathrm{exec}}=5$ trading days) reflects institutional portfolio practice, where allocation decisions are typically made at low-to-moderate frequency to limit transaction costs and market impact. Between decision points, positions are held constant except for bounded execution steps, enforcing temporal consistency and preventing implicit high-frequency trading behavior.

Transaction costs are modeled as proportional costs on weight changes (TC=5 bps), consistent with empirical estimates for liquid equity ETFs. The minimum weight-change threshold ($\delta_{\min}=0.05$) eliminates economically irrelevant trades that would be dominated by noise and costs, while the maximum step size ($W_{\mathrm{step}}^{\max}=0.60$ in training, reduced to 0.40 out-of-sample) enforces gradual rebalancing rather than instantaneous full reallocation.

Risk targeting and volatility scaling.

The Professional Agent targets an annualized volatility of 40%, reflecting the risk profile of an active growth-oriented mandate (e.g., ARKK-like strategies), rather than a volatility-minimizing or market-neutral objective. Volatility scaling adjusts execution size based on realized market volatility, a standard practice in professional risk management that stabilizes risk exposure across regimes without requiring explicit volatility forecasting.

Policy precision and confidence gating.

High policy precision (γ=8.0) and action precision (α=4.0) encode decisive behavior conditional on sufficient evidence, while confidence gating prevents execution when posterior policy beliefs are diffuse. The dual gate—minimum policy probability ($p_{\min}=0.22$) and entropy threshold ($H_{\mathrm{gate}}/H_{\max}=0.96$)—ensures that trades are executed only when the agent’s beliefs are both confident and internally coherent. This mechanism plays the role of a statistical significance filter, analogous to conviction thresholds used by discretionary portfolio managers.

Preferences and utility symmetry.

The Professional Agent uses symmetric utility (λ=1.0) rather than loss-averse preferences, reflecting professional mandates where downside risk is managed through diversification, risk limits, and drawdown controls rather than behavioral asymmetry. Utility scaling ($c_{\mathrm{scale}}=2.0$) ensures that expected returns influence policy selection without overwhelming epistemic considerations.

Epistemic drive and learning discipline.

Epistemic weighting (η) controls the relative contribution of information gain to policy evaluation. In the pymdp implementation, the baseline epistemic weight is η=1.0, which corresponds to the standard expected free energy (EFE) formulation balancing epistemic and pragmatic value without additional amplification. The Professional Agent retains this baseline setting, ensuring that exploration is neither artificially suppressed nor exaggerated relative to the canonical Active Inference specification.

Learning is strictly constrained to the return likelihood modality ($A^{\left(0\right)}$) via surgical Dirichlet updates, while the P&L channel remains frozen by design. This restriction prevents mechanical reinforcement of realized portfolio outcomes and ensures that belief updating reflects changes in exogenous market structure rather than endogenous feedback from the agent’s own actions.

Frozen versus Adaptive regimes.

Frozen test configurations provide a strict transfer benchmark, isolating the economic content of the learned generative model. Adaptive test configurations allow controlled post-training learning, enabling the agent to recalibrate return dynamics under regime shifts while preserving structural integrity. This separation mirrors professional model governance, where live models may adapt within pre-approved boundaries but are otherwise held fixed.

Summary.

Overall, the Professional Agent parameterization encodes a disciplined, risk-aware decision process consistent with institutional portfolio management. Performance differences across regimes arise from the interaction between market structure and belief updating, rather than from implicit leverage, overfitting, or execution artifacts.

Parameter governance.

Table A12, Table A13 and Table A14 jointly define the complete parameterization of the Professional Agent. All parameters not explicitly listed as adaptive are held fixed across training and out-of-sample evaluation. This separation ensures strict transfer integrity, reproducibility, and clear attribution of performance differences to learning dynamics rather than hidden hyperparameter changes.

## Appendix E. Complete Cross-Profile Summary

Purpose.This table provides a consolidated reference-only summary of out-of-sample (OOS) performance and behavioral diagnostics across the three cognitive profiles studied in this work: Bearish, Neutral, and Professional. All metrics are computed over the common test window (25 August 2022–5 December 2025) using daily excess returns relative to the corresponding risk-free rate. Within each profile, Frozen and Adaptive variants differ exclusively in the presence of surgical online learning of the return likelihood $A^{\left(0\right)}$. Cross-profile comparisons are reported for completeness and interpretability, but no claim of economic dominance across cognitive specifications is implied.

**Table A15:** Cross-Profile Out-of-Sample Performance Summary (25 August 2022–5 December 2025). Risk-adjusted and behavioral metrics for Bearish, Neutral, and Professional Adaptive Inference agents evaluated out-of-sample on ARKK. All Sharpe and Sortino ratios are computed on *daily excess returns* relative to the test-period risk-free rate. Adaptive variants differ from Frozen only through continued surgical learning of the return likelihood $A^{\left(0\right)}$. ΔSharpe denotes the within-profile difference (Frozen − Adaptive).

Profile	Mode	Sharpe	Sortino	MaxDD (%)	CAGR (%)	Long (%)	ExecRate (%)	Turnover	ΔSharpe
Bearish	Frozen	−0.71	−0.90	−33.9	−7.7	54.2	29.1	17.9	+0.42
	Adaptive	−1.13	−1.41	−45.8	−12.8	50.4	35.4	21.9	
Neutral	Frozen	−1.44	−1.76	−40.6	−13.2	21.2	67.3	21.4	−1.69
	Adaptive	+0.25	+0.31	−8.8	+7.4	40.0	49.7	15.9	
Professional	Frozen	+0.39	+0.53	−35.8	+12.3	65.0	26.0	14.4	+0.67
	Adaptive	−0.28	−0.35	−41.3	−4.4	65.6	29.8	17.7	
Buy & Hold	—	+0.54	+0.80	−39.6	+20.5	100.0	—	—	—

## Appendix F. Entropy Diagnostics and Statistical Validation

This appendix complements the main out-of-sample Professional Agent (ARKK) results by reporting (i) belief-entropy diagnostics and (ii) the Entropic Sharpe Ratio (ESR) for all profiles, and for SPY as a benchmark control.

Entropy source. Unless otherwise stated, entropy refers to the *policy posterior entropy* $H_{t}\equiv H\;\left(Q(\pi_{t})\right)$, measured in *bits*. Policy entropy captures decisional uncertainty over action sequences and is the entropy diagnostic used in all ESR computations. We summarize its day-to-day variation using $E[|\Delta H_{t}|]$ where $\Delta H_{t}=H_{t}-H_{t-1}$.

**Definition** **A1** (Entropic Sharpe Ratio)**.** *Let $x_{t}$ denote the RF-aware daily excess return series (the same excess-return convention used throughout the paper). Define the annualized excess return as AnnExcess and the mean absolute entropy change as E[|ΔH|] (bits). The Entropic Sharpe Ratio (ESR) is defined as*

(A1) $$\mathrm{ESR}\;\equiv \;\frac{\mathrm{AnnExcess}}{E[|\Delta H|]}\;(\mathrm{units}\;:\;\mathrm{annual}\;\mathrm{excess}\;\mathrm{return}\;\mathrm{per}\;\mathrm{bit}).$$

*where H[·] denotes base-2 Shannon entropy of the policy posterior.*

We report ESR both on All Days and on Decision Days Only (weekly cadence), since policy evaluation and portfolio rebalancing are concentrated at decision times. The decision-only view isolates “performance per bit” on the subset of days when the agent actually evaluates policies and can trade.

Interpretation guidance.

A positive ESR indicates that belief updating is, on average, associated with positive excess performance per bit. Negative ESR indicates that belief updating is associated with negative excess returns per bit. Decision-only ESR can be materially more volatile because it is computed on fewer samples; therefore, we treat it as a diagnostic of *decision-time efficiency* rather than as a standalone performance metric.

### Appendix F.1. ESR Results: Bearish Profile (ARKK)

**Table A16:** Entropic Sharpe Ratio (ESR)—Bearish Profile (ARKK). Entropy source: policy posterior entropy (bits). Excess returns are risk-free-aware. ESR is reported as % annual excess return per bit.

Tag/Mode	Sample	*N*	AnnExcess	E[|ΔH|] (Bits)	ESR (%/Bit)
Train (Bearish)	All days	1922	0.0450	0.2973	15.14
Train (Bearish)	Decision-only	384	0.1781	0.2851	62.46
Test Frozen	All days	822	−0.1139	0.2622	−43.44
Test Frozen	Decision-only	164	−0.2703	0.2380	−113.57
Test Adaptive	All days	822	−0.1729	0.2675	−64.61
Test Adaptive	Decision-only	164	−0.3450	0.2775	−124.33

Reading. The Bearish profile exhibits positive ESR in-sample but strongly negative ESR out-of-sample, consistent with the profile’s OOS underperformance in risk–free–aware terms. The decision-only ESR is more negative, indicating that, conditional on actual decision times, policy belief updating is particularly inefficient in the test regime.

### Appendix F.2. ESR Results: Neutral Agent (ARKK)

**Table A17:** Entropic Sharpe Ratio (ESR): Neutral Agent (ARKK). Entropy source: policy posterior entropy (bits). Excess returns are risk-free-aware. ESR is reported as % annual excess return per bit.

Tag/Mode	Sample	*N*	AnnExcess	E[|ΔH|] (Bits)	ESR (%/Bit)
Test Frozen	All days	823	−0.1808	0.3749	−48.22
Test Frozen	Decision-only	164	−0.1899	0.3491	−54.41
Test Adaptive	All days	823	0.0329	0.0540	60.93
Test Adaptive	Decision-only	164	−0.0050	0.2712	−1.86

Reading. The Neutral Adaptive Agent has a strongly positive ESR on all days, driven by positive excess return and very low average entropy change. However, decision-only ESR is near zero, suggesting that the gain in “return per bit” is realized largely outside the sparse weekly decision times (e.g., through held exposures rather than decision-time updates). This aligns with the broader result that the Neutral Adaptive Agent variant improves OOS risk-adjusted performance primarily through stable positioning and low drawdown.

### Appendix F.3. ESR Results: Professional Profile (ARKK)

**Table A18:** Entropic Sharpe Ratio (ESR): Professional Agent (ARKK). Entropy source: policy posterior entropy (bits). Excess returns are risk-free-aware. ESR is reported as % annual excess return per bit.

Tag/Mode	Sample	*N*	AnnExcess	E[|ΔH|] (Bits)	ESR (%/Bit)
Train (PRO)	All days	1922	0.1541	0.5220	29.52
Train (PRO)	Decision-only	384	0.2878	0.5464	52.67
Test Frozen	All days	822	0.1095	0.4720	23.19
Test Frozen	Decision-only	164	−0.0960	0.4613	−20.81
Test Adaptive	All days	822	−0.0654	0.5204	−12.57
Test Adaptive	Decision-only	164	−0.2604	0.5762	−45.19

Sample size *N* reflects valid $|\Delta H_{t}|$ observations. The first day of each evaluation window is excluded due to the entropy differencing operation ($\Delta H_{t}=H_{t}-H_{t-1}$), yielding N=822 for test samples versus T=823 in level-based tests (e.g., Diebold–Mariano).

Reading. In the Professional Agent profile, the Frozen Agent remains positive in ESR on all days out-of-sample, while the Adaptive Agent is negative. Notably, decision-only ESR turns negative even for the Frozen Agent, indicating that the weekly decision points are not the dominant driver of positive performance; rather, performance is primarily accrued via exposure held between decision times (consistent with bounded execution and low execution rate in the Professional Agent configuration).

### Appendix F.4. ESR Results: SPY Benchmark Universe (Professional Configuration)

**Table A19:** Entropic Sharpe Ratio (ESR): SPY (Professional Agent configuration). Entropy source: policy posterior entropy (bits). Excess returns are risk-free-aware. ESR is reported as annual excess return per bit (dimensionless). The Frozen Agent achieves substantially higher ESR than the adaptive variant, confirming the Passivity Paradox from an information-theoretic perspective.

Tag/Mode	Sample	*N*	AnnExcess	E[|ΔH|] (Bits)	ESR (per Bit)
Train (SPY)	All days	1922	0.0148	0.3923	0.0377
Train (SPY)	Decision-only	384	0.0548	0.4094	0.1338
Test Frozen (SPY)	All days	822	0.0985	0.4358	**0.2261**
Test Frozen (SPY)	Decision-only	164	0.1417	0.4463	**0.3175**
Test Adaptive (SPY)	All days	822	0.0769	0.5021	0.1532
Test Adaptive (SPY)	Decision-only	164	0.0751	0.4801	0.1564

Reading. The SPY results confirm the Passivity Paradox from an information-theoretic perspective. The Frozen Agent achieves ESR of 0.226 (all days) versus 0.153 for the Adaptive Agent—a 48% improvement in return per bit of belief change. On decision days, the gap widens further: Frozen Agent achieves ESR 0.317 versus Adaptive’s 0.156, indicating that frozen beliefs extract *twice* the economic value per unit of informational effort.

Notably, the Frozen Agent exhibits decision-day ESR amplification: ESR increases from 0.226 (all days) to 0.317 (decision days), a 40% improvement. The Adaptive Agent shows no such amplification (0.153→0.156, essentially flat). This asymmetry indicates that frozen beliefs concentrate the information value into executed trades, while adaptive learning dissipates the information effort across noise-driven belief updates that do not translate into excess returns.

The lower entropy expenditure of the Frozen Agent (E[|ΔH|]=0.436 bits) compared to adaptive (0.502 bits) further confirms that frozen beliefs achieve superior returns with less informational “work”—the hallmark of efficient inference under the Passivity Paradox.

### Appendix F.5. Entropy-Regime Conditional Return Differences (Professional Agent OOS)

Entropy definition for regime analysis.

In this subsection only, entropy refers to the posterior entropy of the market-state belief $H[Q\left(s_{t}^{m}\right)]$, which is used to define uncertainty regimes and is distinct from the policy entropy employed in ESR calculations. Figure A8 reports (top) the market-state entropy time series and (bottom) the daily excess return difference $\Delta x_{t}\equiv x_{t}^{\mathrm{Frozen}}-x_{t}^{\mathrm{Adaptive}}$ with a 20-day rolling mean, with background shading indicating tercile-based entropy regimes.

Opportunity Capture Mechanism.

The Frozen Agent’s advantage is *largest* in low-entropy periods (Δμ=+43.4%, p=0.030) where regime structure is clear, diminishes in mid-entropy (+22.6%), and *disappears* in high-entropy (−13.5%, p=0.132). This pattern confirms that:

*Frozen Agent’s beliefs outperform adaptive learning when regime information is present but imperfectly observed.*

The Passivity Paradox operates through opportunity capture, calibrated beliefs enable decisive positioning when regimes are identifiable, while adaptive learning introduces drift that degrades signal extraction even when market structure exists.

To formally test whether mean excess return differences are regime-dependent, we report HAC-robust *t*-tests (Newey–West with the paper’s standard lag choice) for $\Delta r_{t}$ unconditionally and within each entropy tercile. In the Professional Agent OOS sample, the Frozen-minus-Adaptive excess-return difference is positive and statistically significant unconditionally and in Low/Mid entropy terciles, while the High-entropy tercile does not reject a zero mean difference (*p*-value >0.10). The rolling mean is included to highlight persistent regime-level return differentials rather than short-lived noise.

These results support the interpretation that the Frozen Agent configuration is more reliable in low-to-moderate uncertainty regimes, whereas relative performance converges (or reverses) in the highest uncertainty regime.

### Appendix F.6. Statistical Validation and Regime-Conditional Tests

Regime-conditional variance-efficiency tests.

Table A20 reports Diebold–Mariano (DM) tests on squared excess returns for the Professional Agent on ARKK, computed unconditionally and conditional on regime uncertainty measured by the posterior entropy of the market-state belief. Entropy terciles partition the sample into low-, mid-, and high-uncertainty regimes using the same entropy series employed in the main text.

The DM test evaluates differences in mean squared loss between strategies. A positive DM statistic indicates that the first strategy listed has *higher* squared loss (worse variance efficiency) than the comparator, while a negative statistic indicates lower squared loss (better variance efficiency). Heteroskedasticity- and Autocorrelation-Consistent (HAC) standard errors are used throughout with a fixed lag length of 20 trading days.

**Table A20:** Regime-Conditional Diebold–Mariano Tests (Squared Excess Returns).

Entropy Regime	DM Statistic	*p*-Value	Interpretation
Unconditional	+3.99	<0.001 ***	Adaptive variance-efficient
Low Entropy	+3.39	<0.001 ***	Adaptive variance-efficient
Mid Entropy	+3.22	0.001 **	Adaptive variance-efficient
High Entropy	+2.11	0.035 *	Adaptive variance-efficient

*Notes*: Diebold–Mariano (DM) tests are computed on squared daily risk-free-adjusted excess returns using the loss differential $d_{t}=r_{t,\mathrm{Frozen}}^{2}-r_{t,\mathrm{Adaptive}}^{2}$. Positive DM statistics therefore indicate higher mean squared loss (lower variance efficiency) for the Frozen Agent relative to the Adaptive Agent. Entropy regimes are defined by terciles of the market-state posterior entropy (*T* = 823 aligned out-of-sample observations; regime counts: Low = 274, Mid = 274, High = 275). HAC standard errors use Newey–West correction with 20 lags. *** p<0.001, ** p<0.01, * p<0.05.

## Appendix G. Benchmark Models

### Appendix G.1. BSM Real Options Benchmark: Empirical Validation and Limiting Behavior

This section documents the BSM Real Options benchmark used in the empirical comparison with the Professional Agent on the ARKK ETF. The BSM construction is used as a classical probabilistic *signal-generation device* coupled with an execution layer that is explicitly matched to the Professional Agent. We report both formal properties (signal meaning, no-lookahead timing) and empirical validation on the benchmark-safe evaluation calendar.

**Proposition** **A1** (Probability score used as a directional signal)**.** *Fix a trading day t and define the underlying and strike anchor as*

$$S_{t}\equiv P_{t},\;K_{t}\equiv {\mathrm{EMA}}_{60}\left(P_{t}\right),$$

*with volatility $\sigma_{t}$ estimated from the most recent 20 daily returns available at time t and horizon T=21/252 years. When enabled, the daily risk-free return $r_{f,t}^{\left(d\right)}$ is converted to a continuously-compounded annual rate*

$$r_{t}\equiv 252\log\left(1+r_{f,t}^{\left(d\right)}\right).$$

*Under the log-normal score model*

$$\log\;\left(\frac{S_{t+T}}{S_{t}}\right)∼N\;\left(\left(r_{t}-\frac{1}{2}\sigma_{t}^{2}\right)T,\;\sigma_{t}^{2}T\right),$$

*the strategy’s score*

$$p_{\mathrm{long},t}\equiv \Phi \left(d_{2,t}\right)$$

*equals the conditional probability that $S_{t+T}\geq K_{t}$ given information at time t.*

**Proof.** Standardization of the log-normal terminal-price distribution yields $\mathrm{Pr}\left[S_{t+T}\geq K_{t}|F_{t}\right]=\Phi \left(d_{2,t}\right)$.    □

**Remark** **A1** (Interpretation used in the benchmark)**.** *In this study, $\Phi (d_{2})$ is interpreted as a calibrated probability score for regime conviction, not as a claim that ARKK is priced under a risk-neutral measure. This preserves the operational meaning of $\Phi (d_{2})$ while avoiding conflation with a structural asset-pricing hypothesis.*

**Proposition** **A2** (Threshold policy and Professional execution parity)**.** *Define fixed confidence thresholds $\underline{p}=0.48$ and $\bar{p}=0.52$ and target exposure*

$$w_{t}^{★}=\left\{\begin{array}{cc} +1, & p_{\mathrm{long},t}>\bar{p},\\ -1, & p_{\mathrm{long},t}<\underline{p},\\ 0, & \underline{p}\leq p_{\mathrm{long},t}\leq \bar{p}. \end{array}\right.$$

*The realized portfolio weight $w_{t}$ is obtained by applying the following execution layer, matched to the Professional Agent:*

1. *Weekly decision cadence: rebalancing is attempted every τ=5 trading days.*
2. *Volatility scaling on weight changes: define an annualized realized volatility estimate ${\hat{\sigma }}_{t}$ over a 20-day window and a volatility floor $\sigma_{\mathrm{floor}}=0.05$. The scalar applied to weight changes is*
  $$\nu_{t}\;=\;\min\;\left(1,\;\frac{\sigma_{\mathrm{target}}}{\max({\hat{\sigma }}_{t},\;\sigma_{\mathrm{floor}})}\right),\;\sigma_{\mathrm{target}}=0.40.$$
3. *Step-size limiter: the scaled change $\nu_{t}\left(w_{t}^{★}-w_{t-1}\right)$ is clipped to ±0.60 in train and ±0.40 in test.*
4. *Minimum-change filter: trades execute only when $|w_{t}-w_{t-1}|\;\geq \;0.05$.*
5. *Through-flat constraint: sign flips are forced to pass through 0 (no instantaneous long↔short).*

*Transaction costs of 5 bps are charged only on executed trades.*

**Proof.** Each element above corresponds directly to the implementation: (i) TRADE_STEP = 5, (ii) scalar $\nu_{t}$ computed from a 20-day annualized volatility estimate with floor 0.05 and target 0.40, (iii) MAX_STEP_TRAIN = 0.60 and MAX_STEP_TEST = 0.40, (iv) MIN_CHANGE = 0.05, and (v) REQUIRE_FLAT_ON_FLIP = True.    □

**Proposition** **A3** (No-lookahead and Professional excess-return accounting)**.** *The BSM Real Options benchmark is free of look-ahead bias and uses the Professional Agent convention for excess returns:*

1. *The EMA strike anchor and volatility estimate use only information available at or before time t.*
2. *Daily P&L is computed as $w_{t-1}r_{t}$.*
3. *Excess returns are computed as*
  $$r_{t}^{\mathrm{excess}}=w_{t-1}r_{t}-w_{t-1}r_{f,t}^{\left(d\right)},$$
  *so flat periods ($w_{t-1}=0$) incur no artificial risk-free penalty.*

**Proof.** The volatility estimator uses returns up to t−1 by construction, P&L uses the lagged held weight $w_{t-1}$, and the risk-free adjustment is multiplied by the same lagged weight, matching the Professional Agent exactly.    □

**Proposition** **A4** (Empirical behavior on ARKK under benchmark-safe alignment)**.** *Evaluated on the benchmark-safe ARKK calendar aligned to the Professional Agent, the BSM Real Options benchmark exhibits:*

1. *Positive in-sample risk-adjusted performance (train Sharpe =0.498, MaxDD =−44.4%, CAGR ≈11.8%),*
2. *Negative out-of-sample performance (test Sharpe =−0.162, MaxDD =−53.7%, CAGR ≈−10.0%),*
3. *Drawdown mitigation during the 2022 selloff, but delayed re-engagement during the 2023–2024 recovery due to the narrow confidence band around $p_{\mathrm{long}}=0.5$.*

**Proof.** Figure A9 reports stitched gross equity curves on the same evaluation calendar and execution rules. The benchmark’s static probability thresholding yields extended flat/low-exposure intervals, which reduces drawdowns but creates opportunity cost in post-crash regime transitions.    □

**Table A21:** BSM Real Options performance under Professional Agent configuration and risk-free-excess evaluation.

Period	Sharpe	Sortino	MaxDD	CAGR	Decisions	Trades
Train	+0.498	+0.635	−44.4%	+11.8%	385	127
Test	−0.162	−0.196	−53.7%	−10.0%	165	84

**Table A22:** Out-of-sample performance (test period) under identical execution and risk-free conventions.

Model	Sharpe	Sortino	MaxDD	CAGR
BSM Real Options	−0.162	−0.196	−53.7%	−10.0%
Professional Frozen Agent	+0.39	+0.53	−35.8%	+12.3%

**Corollary** **A1** (Limiting interpretation: thresholding $\Phi (d_{2})$ is a band test on $d_{2}$). *Because Φ is strictly increasing, thresholding $\Phi (d_{2,t})$ around 0.5 is equivalent to thresholding the normalized log-moneyness score*

$$d_{2,t}=\frac{\ln\left(S_{t}/K_{t}\right)+\left(r_{t}-\frac{1}{2}\sigma_{t}^{2}\right)T}{\sigma_{t}\sqrt{T}}$$

*in an upper/lower band around 0. Empirically, when $p_{\mathrm{long},t}\approx 0.5$ (equivalently $d_{2,t}\approx 0$), the strategy remains flat, consistent with the diagnostics evidence.*

**Proof.** Immediate from monotonicity of Φ and the definition of the thresholds $\underline{p}=0.5-\varepsilon$, $\bar{p}=0.5+\varepsilon$.    □

### Appendix G.2. Stochastic Discounted Cash Flow Benchmark

This appendix documents the Stochastic discounted cash flow (DCF) benchmark used in the empirical comparison. The model combines a Bayesian state-space model for cash-flow growth estimation with Monte Carlo valuation and a Professional Agent style execution layer identical to that used for the BSM Real Options and Adaptive Inference benchmarks.

### Appendix G.3. Data Alignment and Evaluation Windows

All inputs are aligned to the canonical Professional Agent evaluation calendar to ensure benchmark safety and eliminate look-ahead bias.

- Train period: 2015-01-06 to 2022-08-24 (N=1923 trading days),
- Test period: 2022-08-26 to 2025-12-05 (M=823 trading days),
- Gap: A two-day gap separates train and test windows.

Prices, returns, and risk-free rates are strictly reindexed to this calendar, with prices forward-filled to returns and never vice versa.

### Appendix G.4. Discount Rate Construction

The annual discount rate combines a geometrically annualized daily risk-free rate with the [103] implied equity risk premium (ERP):

(A2) $$r_{t}^{\left(\mathrm{ann}\right)}={\left(1+r_{f,t}^{\left(d\right)}\right)}^{252}-1+{\mathrm{ERP}}_{t},$$

where $r_{f,t}^{\left(d\right)}$ is the daily risk-free return and ${\mathrm{ERP}}_{t}$ is the smoothed implied equity risk premium, forward-filled at the daily frequency.

### Appendix G.5. Bayesian Kalman Dynamic Linear Model

Log cash flows are modeled using a two-dimensional Dynamic Linear Model (DLM):

$$x_{t}=\left[\begin{array}{c} x_{t}\\ g_{t} \end{array}\right],$$

where $x_{t}$ is the log cash-flow level and $g_{t}$ is the daily growth increment.

The state transition equation is

(A3) $$x_{t}=A\left(\rho \right)\;x_{t-1}+\varepsilon_{t},\;A\left(\rho \right)=\left[\begin{array}{cc} 1 & 1\\ 0 & \rho \end{array}\right],$$

with Gaussian process noise $\varepsilon_{t}∼N\left(0,Q\right)$.

Observations follow

(A4) $$y_{t}=C\;x_{t}+\eta_{t},\;C=\left[\begin{array}{cc} 1 & 0 \end{array}\right],$$

where $\eta_{t}∼N\left(0,R\right)$.

Parameters (ρ,Q,R) are estimated on the training sample via EM with RTS smoothing. The estimated values are

$$\hat{\rho }=0.990,\;\mathrm{diag}\left(\hat{Q}\right)=\left[10^{-6},\;10^{-6}\right],\;\hat{R}=7.203\times 10^{-4}.$$

### Appendix G.6. Monte Carlo Valuation

Daily growth estimates are annualized as

(A5) $$g_{t}^{\left(\mathrm{ann}\right)}=252\;g_{t}^{\left(\mathrm{daily}\right)},\;\sigma_{g,t}^{\left(\mathrm{ann}\right)}=\sqrt{252}\;\sigma_{g,t}^{\left(\mathrm{daily}\right)}.$$

Cash flows are treated as an annual run-rate proxy

$$C_{0,t}=\alpha \;P_{t},\;\alpha =0.003$$

and projected over a 10-year horizon using monthly steps. The present value is computed as the discounted sum of projected cash flows plus a terminal value

(A6) $${\mathrm{TV}}_{t}=\frac{C_{N,t}\left(1+g_{\infty }\right)}{r_{t}^{\left(\mathrm{ann}\right)}-g_{\infty }},\;g_{\infty }=\min\left(0.025,\;0.95\;r_{t}^{\left(\mathrm{ann}\right)}\right),$$

where $C_{N,t}$ is the terminal-period annual cash-flow run-rate.

Monte Carlo valuation uses 3000 fixed paths per day.

### Appendix G.7. Execution Parity with the Professional Agent and BSM Real Options Model

Trading execution is identical to the BSM Real Options and Professional Agent benchmarks:

- Weekly decision cadence (TRADE_STEP=5),
- Volatility scaling toward target $\sigma_{\mathrm{target}}=40\%$ with floor $\sigma_{\min}=5\%$,
- Maximum position change per decision: 0.60 (train), 0.40 (test),
- Minimum change filter |Δw|≥0.05,
- Through-flat constraint on sign reversals,
- Transaction cost of 5 bps per unit change in position.

Daily P&L follows the Professional Agent convention $w_{t-1}r_{t}$, with risk-free excess returns used for Sharpe and Sortino ratios.

### Appendix G.8. Valuation Robustness and Signal Clamping

Raw fair values can become extreme under persistent growth and long horizons. To ensure economically interpretable trading signals, valuation outputs are transformed into bounded signals:

(A7) $$\begin{array}{cc} FV_{t}^{\left(\mathrm{sig}\right)}\; & =\min\;\left(\max\left(FV_{t}^{\left(\mathrm{raw}\right)},0\right),\;\kappa P_{t}\right), \end{array}$$

(A8) $$\begin{array}{cc} m_{t}^{\left(\mathrm{sig}\right)}\; & =\mathrm{clip}\;\left(\frac{FV_{t}^{\left(\mathrm{sig}\right)}-P_{t}}{P_{t}},\;m_{\min},\;m_{\max}\right), \end{array}$$

with $\left(m_{\min},m_{\max}\right)=\left(-0.95,\;5.0\right)$ and κ=10.

**Remark** **A2** (Valuation instability on growth assets)**.** *Astronomical raw valuations ($FV_{t}^{\left(\mathrm{raw}\right)}∼10^{21}$ on worst days) arise from the interaction of near-unit-root persistence ($\hat{\rho }=0.99$), multi-year compound growth, and terminal-value dominance when g≈r. This instability is a structural feature of applying DCF methods to assets with negligible current cash flows, not a numerical error. Signal-side clamps ensure bounded trading behavior while preserving raw outputs for diagnostic transparency.*

### Appendix G.9. Performance Results

Table A23 reports Professional Agent-style risk-free excess performance.

**Table A23:** Stochastic DCF performance under Professional Agent-style risk-free-excess evaluation.

Period	Sharpe	Sortino	MaxDD	CAGR	Decisions	Trades
Train	0.264	0.328	−36.8%	3.6%	385	238
Test	−0.518	−0.621	−70.7%	−21.5%	165	119

**Table A24:** Out-of-sample performance (test period) under identical execution and risk-free conventions.

Model	Sharpe	Sortino	MaxDD	CAGR
Stochastic DCF (Kalman + MC)	−0.52	−0.62	−70.7%	−21.5%
Professional Frozen	+0.39	+0.53	−35.8%	+12.3%

Interpretation.

The Stochastic DCF benchmark performs poorly out of sample, reflecting the sensitivity of discounted cash flow valuation to persistent growth assumptions and terminal value dominance in assets with negligible current cash flows. While Bayesian filtering captures trending dynamics during the train period, the model does not adapt effectively to post-2022 regime changes under fixed structural assumptions.

## Appendix H. Hedge (Multiplicative Weights Update) Benchmark

### Appendix H.1. Construction

The Hedge benchmark implements a full-information multiplicative-weights expert mixture [23] over eight classical trading strategies, each executed through the same friction and risk layer as the Professional Agent (5 bps costs, 5-day cadence, volatility scaling, [−1,1] position bounds, flat-on-flip). The expert panel comprises: Buy & Hold, Vol-Targeted B&H, TSMOM(252-21), Mean Reversion (z=1.5), Trend Filter (MA200), Uncertainty Risk-Off, Short Momentum (21d), and Vol Breakout. At each decision *t*, Hedge observes expert net payoffs from the completed window $\left(t_{\mathrm{prev}},t\right]$, updates the simplex via exponential payoff reweighting, and executes the blended target under the end-of-day convention. The learning rate follows the minimax formula $\eta^{*}=\sqrt{8\ln K/(c^{2}T_{\mathrm{dec}})}=2.00$ (K=8, c=0.05, $T_{\mathrm{dec}}\;=\;164$ effective updates), with payoffs clipped to ±5% per 5-day window.

### Appendix H.2. Post Hoc Validity Audit

Table A25 summarizes the three-part invariance audit on the out-of-sample period (25 August 2022–5 December 2025; 824 d, 165 decisions).

**Table A25:** Hedge Benchmark Validity Audit (ARKK, OOS). End-of-day convention: observe close → update → execute.

Test	Statistic	Threshold	Perm. *p*	Result
1. No look-ahead (Δw vs. $r_{t}$)	$|\rho_{P}\left|\;=\;0.138,\;\right|\rho_{S}|\;=\;0.147$	|ρ|<0.20	0.072	**PASS**
2. Friction-identical payoffs	max|Δ|=0.0	<$10^{-8}$	—	**PASS**
3a. Lagged vol identity	max|Δσ|=0.0	<$10^{-12}$	—	**PASS**
3b. Execution determinism	165/165 exact	<$10^{-12}$	—	**PASS**

Cadence: 5 d. Costs: 5 bps. Clip: ±5%/window. Permutation: 5-day block-shuffle, 5000 iter.

Test 1 (No same-day look-ahead). Weight changes $\Delta w_{t}$ on the full decision grid show $|\rho_{P}|\;=\;0.138$ and $|\rho_{S}|\;=\;0.147$, both below the economic threshold of 0.20. A block-shuffle permutation test (5-day blocks, 5000 iterations) yields p=0.072; the observed |ρ| falls below the 95th null percentile (0.151). The mild positive correlation is consistent with adaptive reweighting under serially correlated returns and does not imply contemporaneous information leakage.

Test 2 (Friction-identical payoffs). Expert payoff vectors are recomputed via the canonical daily-return function; all entries match bitwise (max|Δ|=0), confirming that the learning signal is provably identical to the evaluation accounting.

Test 3 (Lagged vol & execution determinism). Instrumented audit data confirms ${\hat{\sigma }}_{\mathrm{used}}\equiv {\hat{\sigma }}_{t-1}$ (bitwise exact) and replaying execute_trade reproduces all 165 weights with zero error.

Clipping disclosure. Payoff clipping affects 49.1% of evaluation windows (at least one expert clipped) and 19.2% of (expert, window) pairs. Window-level clipping is high (49.1%) due to ARKK’s volatility regime, while pair-level clipping is moderate (19.2%). The theoretical regret bound is therefore approximate; this does not affect the invariance tests.

### Appendix H.4. Sensitivity

**Table A26:** Learning-rate and transaction-cost sensitivity (ARKK, OOS).

*Panel A: Learning Rate* (Cost =5 bps)						*Panel B: Transaction Cost* (η=2.0)					
η	Sharpe	Sort.	MaxDD	CAGR	Turn.	bps	Sharpe	Sort.	MaxDD	CAGR	Turn.
0.01	0.51	0.78	−25%	14.2	11.0	0	0.40	0.56	−26%	11.4	11.8
0.10	0.50	0.76	−25%	14.0	11.0	5 *	0.40	0.55	−26%	11.2	11.7
0.50	0.48	0.72	−25%	13.5	11.1	10	0.39	0.54	−26%	11.1	11.7
1.00	0.45	0.66	−26%	12.7	11.3	25	0.37	0.51	−26%	10.6	11.6
2.00 *	0.40	0.55	−26%	11.2	11.7	50	0.33	0.46	−27%	9.9	11.5

* Primary config (minimax $\eta^{*}$, matched to the Professional Agent). Sharpe ratio σ across η: 0.044; range: 0.112.

Sharpe ratios vary within a narrow band across η∈[0.01,2.0] (range =0.112, σ=0.044), indicating low sensitivity to learning-rate choice. Cost sensitivity is monotonic: Sharpe ratio declines from +0.40 (0 bps) to +0.33 (50 bps), indicating economically reasonable friction robustness.

### Appendix H.5. Interpretation

Out-of-sample (2022–2025), Hedge achieves a Sharpe ratio of +0.40 with MaxDD −26.0%, comparable to the Professional Frozen Agent (Sharpe ratio +0.39, MaxDD −35.8%) but with substantially better drawdown control, confirming it as a credible adversarial benchmark.

However, unlike the Professional Agent, Hedge lacks an explicit generative model and cannot decompose decisions into belief states, epistemic uncertainty, or expected information gain. The comparable risk-adjusted return therefore isolates the structural advantage of the Adaptive Inference framework: explicit belief states, epistemic value, and ante hoc decision auditability—properties unattainable by any convex combination of fixed classical strategies.

## References

[1] Shiller R.J. (1981). Do stock prices move too much to be justified by subsequent changes in dividends?. Am. Econ. Rev..

[2] Shiller R.J. (2024). U.S. Stock Markets 1871–Present and CAPE Ratio. Online Dataset. https://www.econ.yale.edu/~shiller/data.htm.

[3] Bordalo P., Gennaioli N., Shleifer A. (2021). Corrigendum: Diagnostic expectations and credit cycles. J. Financ..

[4] Bordalo P., Gennaioli N., Shleifer A. (2022). Overreaction and diagnostic expectations in macroeconomics. J. Econ. Perspect..

[5] Bordalo P., Gennaioli N., Shleifer A. (2020). Diagnostic Expectations and Credit Cycles. J. Financ..

[6] Bianchi F., Ilut C.L., Saijo H. (2024). Smooth Diagnostic Expectations.

[7] L’Huillier J.P., Singh S.R., Yoo D. (2023). Incorporating diagnostic expectations into the New Keynesian framework. Rev. Econ. Stud..

[8] Füllbrunn S., Huber C., Eckel C., Weitzel U. (2024). Heterogeneity of beliefs and trading behavior: A reexamination. J. Financ. Quant. Anal..

[9] Kahneman D., Tversky A. (1979). Prospect theory: An analysis of decision under risk. Econometrica.

[10] Sims C.A. (2003). Implications of Rational Inattention. J. Monet. Econ..

[11] Gabaix X. (2014). A sparsity-based model of bounded rationality. Q. J. Econ..

[12] Lo A.W. (2005). Reconciling efficient markets with behavioral finance: The adaptive markets hypothesis. J. Invest. Consult..

[13] Lo A.W. (2004). The adaptive markets hypothesis: Market efficiency from an evolutionary perspective. J. Portf. Manag..

[14] Lucas R.E. (1976). Econometric Policy Evaluation: A Critique. Carnegie-Rochester Conf. Ser. Public Policy.

[15] Friston K.J., Kilner J., Harrison L. (2006). A free energy principle for the brain. J. Physiol.-Paris.

[16] Friston K. (2009). The free-energy principle: A rough guide to the brain?. Trends Cogn. Sci..

[17] Friston K.J., Rigoli F., Ognibene D., Mathys C., Fitzgerald T., Pezzulo G. (2015). Active inference and epistemic value. Cogn. Neurosci..

[18] Da Costa L., Sajid N., Parr T., Friston K., Smith R. (2023). Reward Maximization Through Discrete Active Inference. Neural Comput..

[19] Fields C., Goldstein A., Sandved-Smith L. (2024). Making the Thermodynamic Cost of Active Inference Explicit. Entropy.

[20] Ortega P.A., Braun D.A. (2013). Thermodynamics as a Theory of Decision-Making with Information-Processing Costs. Proc. R. Soc. A.

[21] Ramstead M.J.D., Kirchhoff M.D., Friston K.J. (2020). A Tale of Two Densities: Active Inference Is Enactive Inference. Adapt. Behav..

[22] Friston K. (2010). The free-energy principle: A unified brain theory?. Nat. Rev. Neurosci..

[23] Freund Y., Schapire R.E. (1997). A decision-theoretic generalization of on-line learning and an application to boosting. J. Comput. Syst. Sci..

[24] Arora S., Hazan E., Kale S. (2012). The multiplicative weights update method: A meta-algorithm and applications. Theory Comput..

[25] Hamilton J.D. (1989). A New Approach to the Economic Analysis of Nonstationary Time Series and the Business Cycle. Econometrica.

[26] Merton R.C. (1971). Optimum Consumption and Portfolio Rules in a Continuous-Time Model. J. Econ. Theory.

[27] Sharpe W.F. (1964). Capital asset prices: A theory of market equilibrium under conditions of risk. J. Financ..

[28] Stokey N.L., Lucas R.E., Prescott E.C. (1989). Recursive Methods in Economic Dynamics.

[29] Friston K.J., FitzGerald T., Rigoli F., Schwartenbeck P., Pezzulo G. (2017). Active Inference: A Process Theory. Neural Comput..

[30] Smith R., Friston K.J., Whyte C.J. (2022). A Step-by-Step Tutorial on Active Inference and Its Application to Empirical Data. J. Math. Psychol..

[31] Parr T., Pezzulo G., Friston K.J. (2022). Active Inference: The Free Energy Principle in Mind, Brain, and Behavior.

[32] Friston K., Da Costa L., Sajid N., Heins C., Ueltzhöffer K., Pavliotis G.A., Parr T. (2023). The free energy principle made simpler but not too simple. Phys. Rep..

[33] Landauer R. (1961). Irreversibility and heat generation in the computing process. IBM J. Res. Dev..

[34] Still S., Sivak D.A., Bell A.J., Crooks G.E. (2012). Thermodynamics of prediction. Phys. Rev. Lett..

[35] Simon H.A. (1955). A behavioral model of rational choice. Q. J. Econ..

[36] Simon H.A. (1957). Models of Man: Social and Rational.

[37] Montañez S., Quezada-Téllez L.A., Moya-Albor E. (2025). Bayesian mechanics of economic choice: Computational foundations of economic behavior. J. Res. Comput. Sci..

[38] Markowitz H. (1952). Portfolio Selection. J. Financ..

[39] Pearl J. (2009). Causality: Models, Reasoning, and Inference.

[40] Peters J., Janzing D., Schölkopf B. (2017). Elements of Causal Inference: Foundations and Learning Algorithms.

[41] DeMiguel V., Garlappi L., Uppal R. (2009). Optimal Versus Naive Diversification: How Inefficient is the 1/N Portfolio Strategy?. Rev. Financ. Stud..

[42] Black F., Scholes M. (1973). The Pricing of Options and Corporate Liabilities. J. Political Econ..

[43] Merton R.C. (1973). Theory of Rational Option Pricing. Bell J. Econ. Manag. Sci..

[44] Damodaran A. (2012). Investment Valuation: Tools and Techniques for Determining the Value of Any Asset.

[45] Lucas R.E. (1972). Expectations and the neutrality of money. J. Econ. Theory.

[46] Kaelbling L.P., Littman M.L., Cassandra A.R. (1998). Planning and Acting in Partially Observable Stochastic Domains. Artif. Intell..

[47] Smallwood R.D., Sondik E.J. (1973). The Optimal Control of Partially Observable Markov Processes Over a Finite Horizon. Oper. Res..

[48] Da Costa L., Parr T., Sajid N., Veselic S., Neacsu V., Friston K. (2020). Active Inference on Discrete State-Spaces: A Synthesis. J. Math. Psychol..

[49] Friston K. (2013). Life as We Know It. J. R. Soc. Interface.

[50] Dauwels J. (2007). On Variational Message Passing on Factor Graphs. Proceedings of the IEEE International Symposium on Information Theory.

[51] Bishop C.M. (2006). Pattern Recognition and Machine Learning.

[52] Friston K., FitzGerald T., Rigoli F., Schwartenbeck P., O’Doherty J., Pezzulo G. (2016). Active inference and learning. Neurosci. Biobehav. Rev..

[53] Kappen H.J. (2005). Path integrals and symmetry breaking for optimal control theory. J. Stat. Mech. Theory Exp..

[54] Haarnoja T., Zhou A., Abbeel P., Levine S. Soft actor-critic: Off-policy maximum entropy deep reinforcement learning with a stochastic actor. Proceedings of the 35th International Conference on Machine Learning.

[55] Grossman S.J., Stiglitz J.E. (1980). On the impossibility of informationally efficient markets. Am. Econ. Rev..

[56] Van Nieuwerburgh S., Veldkamp L. (2010). Information acquisition and under-diversification. Rev. Econ. Stud..

[57] Parr T., Benrimoh D.A., Vincent P., Friston K.J. (2018). Precision and false perceptual inference. Front. Integr. Neurosci..

[58] Lo A.W., Mamaysky H., Wang J. (2000). Foundations of technical analysis: Computational algorithms, statistical inference, and empirical implementation. J. Financ..

[59] Parr T., Pezzulo G. (2021). Understanding, explanation, and active inference. Front. Syst. Neurosci..

[60] Todorov E. Linearly solvable Markov decision problems. Proceedings of the Advances in Neural Information Processing Systems.

[61] Engle R.F. (2001). GARCH 101: The Use of ARCH/GARCH Models in Applied Econometrics. J. Econ. Perspect..

[62] Bernardi M., Bianchi D., Bianco N. (2022). Smoothing volatility targeting. arXiv.

[63] Moreira A., Muir T. (2017). Volatility-managed portfolios. J. Financ..

[64] Aldridge I. (2013). High-Frequency Trading: A Practical Guide to Algorithmic Strategies and Trading Systems.

[65] Acemoglu D., Ozdaglar A.E., Tahbaz-Salehi A. (2015). Systemic Risk in Endogenous Financial Networks. Columbia Business School Research Paper No. 15-17. https://www.semanticscholar.org/paper/Systemic-Risk-in-Endogenous-Financial-Networks-Acemoglu-Ozdaglar/29a598cc0195a481b2e10444422b9bb0a5c03c3d.

[66] Brunnermeier M.K., Pedersen L.H. (2009). Market liquidity and funding liquidity. Rev. Financ. Stud..

[67] Frazzini A., Israel R., Moskowitz T.J. Trading Costs of Asset Pricing Anomalies. Fama-Miller Working Paper; Chicago Booth Research Paper No. 14-05. https://papers.ssrn.com/sol3/papers.cfm?abstract_id=2294498.

[68] Frazzini A., Israel R., Moskowitz T.J. (2018). Trading Costs. SSRN Working Paper. https://papers.ssrn.com/sol3/papers.cfm?abstract_id=3229719.

[69] Bailey D., Borwein J., López de Prado M. (2014). The Probability of Backtest Overfitting.

[70] Chan L.K.C., Lakonishok J. (1997). Institutional equity trading costs: NYSE versus Nasdaq. J. Financ..

[71] Gârleanu N., Pedersen L.H. (2013). Dynamic trading with predictable returns and transaction costs. J. Financ..

[72] Inghelbrecht K., Tedde M. (2024). Overconfidence, financial literacy and excessive trading. J. Econ. Behav. Organ..

[73] ARK Investment Management LLC ARK Innovation ETF (ARKK) Annual Distribution Disclosures. Fund Distribution Disclosures, Rule 19a-1 Compliance Documents, 2020–2025. Annual Capital Gains and Income Distribution Reports. https://www.ark-funds.com/download-fund-materials#docsListing.

[74] Yahoo Finance (2025). ARK Innovation ETF (ARKK) Historical Price Data. https://finance.yahoo.com/quote/ARKK.

[75] Alpha Vantage Inc. (2025). 3-Month Treasury Bill Rate. https://www.alphavantage.co/documentation/.

[76] Campbell J.Y., Thompson S.B. (2008). Predicting Excess Stock Returns Out of Sample: Can Anything Beat the Historical Average?. Rev. Financ. Stud..

[77] Efron B., Tibshirani R. (1987). Better Bootstrap Confidence Intervals. J. Am. Stat. Assoc..

[78] Ledoit O., Wolf M. (2008). Robust Performance Hypothesis Testing with the Sharpe Ratio. J. Empir. Financ..

[79] Dixit A.K., Pindyck R.S. (1994). Investment Under Uncertainty.

[80] Trigeorgis L., Rueter J.J. (2017). Real options theory in strategic management. Strateg. Manag. J..

[81] Williams J.B. (1938). The Theory of Investment Value.

[82] Kalman R.E. (1960). A new approach to linear filtering and prediction problems. J. Basic Eng..

[83] West M., Harrison J. (1997). Multi-process models. Bayesian Forecasting and Dynamic Models.

[84] Gordon M.J. (1962). The Investment, Financing, and Valuation of the Corporation. Rev. Econ. Stat..

[85] Cesa-Bianchi N., Lugosi G. (2006). Prediction, Learning, and Games.

[86] Li B., Hoi S.C.H. (2014). Online portfolio selection: A survey. ACM Comput. Surv..

[87] Helmbold D.P., Schapire R.E., Singer Y., Warmuth M.K. (1998). On-line portfolio selection using multiplicative updates. Math. Financ..

[88] Cover T.M. (1991). Universal portfolios. Math. Financ..

[89] Sun S., Wang R., An B. (2023). Reinforcement learning for quantitative trading. ACM Trans. Intell. Syst. Technol..

[90] Friston K.J., Frith C.D. (2015). A duet for one. Conscious. Cogn..

[91] Soros G. (2003). The Alchemy of Finance.

[92] Arthur W.B., Holland J.H., LeBaron B., Palmer R., Tayler P. (1997). Asset pricing under endogenous expectations in an artificial stock market. Econ. Notes.

[93] Hommes C.H., Tesfatsion L., Judd K.L. (2006). Heterogeneous agent models in economics and finance. Handbook of Computational Economics.

[94] Farmer J.D., Foley D. (2009). The economy needs agent-based modelling. Nature.

[95] Ghafarollahi A., Buehler M.J. (2025). SciAgents: Automating scientific discovery through bioinspired multi-agent intelligent graph reasoning. Adv. Mater..

[96] Buehler M.J. (2025). Agentic deep graph reasoning yields self-organizing knowledge networks. J. Mater. Res..

[97] Geman S., Bienenstock E., Doursat R. (1992). Neural Networks and the Bias/Variance Dilemma. Neural Comput..

[98] Hastie T., Friedman J., Tibshirani R. (2001). The Elements of Statistical Learning: Data Mining, Inference, and Prediction.

[99] Schreiber T. (2000). Measuring Information Transfer. Phys. Rev. Lett..

[100] Heins C., Millidge B., Da Costa L., Mann S., Friston K., Sajid N. (2022). pymdp: A Python Library for Active Inference in Discrete State Spaces. J. Open Source Softw..

[101] Moskowitz T.J., Ooi Y.H., Pedersen L.H. (2012). Time series momentum. J. Financ. Econ..

[102] Poterba J.M., Summers L.H. (1986). The persistence of volatility and stock market fluctuations. Am. Econ. Rev..

[103] Damodaran A. (2023). Equity Risk Premiums (ERP): Determinants, Estimation and Implications—The 2023 Edition. SSRN Electron. J..

